# The effect of early treatment with ivermectin on viral load, symptoms and humoral response in patients with non-severe COVID-19: A pilot, double-blind, placebo-controlled, randomized clinical trial

**DOI:** 10.1016/j.eclinm.2020.100720

**Published:** 2021-01-19

**Authors:** Carlos Chaccour, Aina Casellas, Andrés Blanco-Di Matteo, Iñigo Pineda, Alejandro Fernandez-Montero, Paula Ruiz-Castillo, Mary-Ann Richardson, Mariano Rodríguez-Mateos, Carlota Jordán-Iborra, Joe Brew, Francisco Carmona-Torre, Miriam Giráldez, Ester Laso, Juan C. Gabaldón-Figueira, Carlota Dobaño, Gemma Moncunill, José R. Yuste, Jose L. Del Pozo, N.Regina Rabinovich, Verena Schöning, Felix Hammann, Gabriel Reina, Belen Sadaba, Mirian Fernández-Alonso

**Affiliations:** a ISGlobal, Hospital Clínic - Universitat de Barcelona, Barcelona, Spain; b Clínica Universidad de Navarra, Pamplona, Spain; c Ifakara Health Institute, Ifakara, Tanzania; d Facultat de Medicina, Universitat de Barcelona, Barcelona, Spain; e Databrew, Gainesville, FL, United States; f IdiSNA, Navarra Institute for Health Research, Pamplona, Spain; g Harvard T.H. Chan School of Public Health, Boston, MA, United States; h Clinical Pharmacology and Toxicology, Department of General Internal Medicine, Inselspital, Bern University Hospital, University of Bern, Switzerland

**Keywords:** Ivermectin, SARS-CoV-2, COVID-19, Anosmia, Hyposmia

## Abstract

**Background:**

Ivermectin inhibits the replication of SARS-CoV-2 in vitro at concentrations not readily achievable with currently approved doses. There is limited evidence to support its clinical use in COVID-19 patients. We conducted a Pilot, randomized, double-blind, placebo-controlled trial to evaluate the efficacy of a single dose of ivermectin reduce the transmission of SARS-CoV-2 when administered early after disease onset.

**Methods:**

Consecutive patients with non-severe COVID-19 and no risk factors for complicated disease attending the emergency room of the Clínica Universidad de Navarra between July 31, 2020 and September 11, 2020 were enrolled. All enrollments occurred within 72 h of onset of fever or cough. Patients were randomized 1:1 to receive ivermectin, 400 mcg/kg, single dose (*n* = 12) or placebo (*n* = 12). The primary outcome measure was the proportion of patients with detectable SARS-CoV-2 RNA by PCR from nasopharyngeal swab at day 7 post-treatment. The primary outcome was supported by determination of the viral load and infectivity of each sample. The differences between ivermectin and placebo were calculated using Fisher's exact test and presented as a relative risk ratio. This study is registered at ClinicalTrials.gov: NCT04390022.

**Findings:**

All patients recruited completed the trial (median age, 26 [IQR 19–36 in the ivermectin and 21–44 in the controls] years; 12 [50%] women; 100% had symptoms at recruitment, 70% reported headache, 62% reported fever, 50% reported general malaise and 25% reported cough). At day 7, there was no difference in the proportion of PCR positive patients (RR 0·92, 95% CI: 0·77–1·09, *p* = 1·0). The ivermectin group had non-statistically significant lower viral loads at day 4 (*p* = 0·24 for gene E; *p* = 0·18 for gene N) and day 7 (*p* = 0·16 for gene E; *p* = 0·18 for gene N) post treatment as well as lower IgG titers at day 21 post treatment (*p* = 0·24). Patients in the ivermectin group recovered earlier from hyposmia/anosmia (76 vs 158 patient-days; *p* < 0.001).

**Interpretation:**

Among patients with non-severe COVID-19 and no risk factors for severe disease receiving a single 400 mcg/kg dose of ivermectin within 72 h of fever or cough onset there was no difference in the proportion of PCR positives. There was however a marked reduction of self-reported anosmia/hyposmia, a reduction of cough and a tendency to lower viral loads and lower IgG titers which warrants assessment in larger trials.

**Funding:**

ISGlobal, Barcelona Institute for Global Health and Clínica Universidad de Navarra.

**Research in context**

**Evidence before this study**

Ivermectin is a broad spectrum antiparasitic drug with known antiviral properties. On April 3, 2020, Caly et al. published evidence from in vitro experiments showing that ivermectin can inhibit the replication of SARS-CoV-2 at micromolar concentrations. This has torn the scientific community into two opposing views, one group calling for avoiding investment and effort in a drug likely to fail clinical trials, and another group calling for rapid scale-up even in the absence of proven safety and efficacy for the potential COVID-19 indication. Since April 2020, there has been an abundance of observational trials, case series and ecological analyses suggesting a potential efficacy of ivermectin against COVID-19. Yet very few reports of rigorously conducted randomized controlled clinical trials. Emerging evidence from studies conducted in Bangladesh and Argentina points towards a potential clinical use.

**Added value of this study**

This pilot, randomized, placebo-controlled, double blind trial failed to show a reduction in the proportion of PCR-positive patients seven days after ivermectin treatment; yet it shows a reduction in the self-reported anosmia/hyposmia and a (non-statistically significant) tendency to lower viral loads and lower IgG titers which presumably reflect milder disease.

**Implications of all the available evidence**

The positive signal found in this pilot together with emerging evidence from animal models and other clinical trials warrants the conduction of larger trials using ivermectin for the early treatment of COVID-19.

Alt-text: Unlabelled box

## 1 Introduction

As of December 21, 2020, there have been over 70 million cases and 1·6 million COVID-19 deaths worldwide [1]. Although the threshold is difficult to predict accurately [2], the spread of SARS-CoV-2 is unlikely to stop before at least 50% of the population has gained immunity, either by vaccination or recovering from a naturally-acquired infection [3]. There are now promising vaccines candidates advancing to emergency regulatory approval [4], but there is a projected delay in global access to the level required for population impact on the trajectory of the pandemic. While efforts are ongoing to develop treatment options, relatively less attention has been devoted to evaluating drug-based transmission blocking or transmission reduction strategies. These strategies would consist in administering a drug with the aim of reducing onward transmission by those infected and could serve to reduce the burden on health system and gain time until vaccines are fully tested and scaled-up.

Ivermectin is a widely used antiparasitic drug with known partial efficacy against several single-strain RNA viruses [5], [6], [7]. Caly et al. reported in vitro inhibition of SARS-CoV-2 replication using micromolar concentrations of ivermectin [8]. These findings, together with early observational evidence and ecological evidence, prompted several Latin-American countries to include ivermectin as part of the national policy for COVID-19 treatment [9].

As of December 21, 2020, there are 45 studies evaluating the efficacy of ivermectin to treat or prevent COVID-19 registered in clinicaltrials.gov, and 74 trials registered in WHO´s International Clinical Trials Registry Platform (https://apps.who.int/trialsearch/) of which at least 14 are already completed. Although some observational and case control studies as well as emerging small randomized clinical trials suggest a potential utility [10], [11], [12], [13]. Yet, there is still a dearth of robust, randomized controlled trials to appropriately inform policy decisions.

This trial was designed as a pilot to evaluate whether the maximum approved dose of ivermectin in Europe could have an impact on the transmission of SARS-CoV-2 when administered early after disease onset.

## 2 Methods

This was a pilot, double-blind, placebo-controlled, single-center, parallel-arm, superiority, randomized clinical trial that compared a single dose of ivermectin with placebo in patients with non-severe COVID-19 and no risk factors. The trial protocol was published [14], the last version of the protocol and statistical analysis plan are available as supplementary files. The protocol was approved by the Spanish national ethics committee for drug research (Hospital Puerta de Hierro Majadahonda) and by the Spanish Agency of Medicines and Medical Devices. All procedures were conducted in compliance with the latest revision of the Helsinki Declaration and Good Clinical Practice. All patients provided verbal informed consent at enrollment followed by written consent once their isolation was lifted in accordance to the EMA recommendations: “Guidance on the Management of Clinical Trials during the COVID-19 (Coronavirus) pandemic Version 2 (27/03/2020)” [15]. This study is registered at ClinicalTrials.gov: NCT04390022. This study was funded by ISGlobal and the Clínica Universidad de Navarra. The funding sources had no role on the design, analysis or decision to publish the results of this study.

### 2.1 Patients

Consecutive outpatients attending the Emergency Room of the Clínica Universidad de Navarra (Pamplona, Spain) with symptoms compatible with COVID-19, no more than 72 h of fever or cough and a positive PCR for SARS-CoV-2 were enrolled. Patients with positive IgG against SARS-CoV-2, comorbidities considered risk factors for severe disease or COVID-19 pneumonia at baseline were excluded (detailed eligibility criteria are provided in the protocol -Supplementary file-).

### 2.2 Study design and oversight

The trial was conducted in the Pamplona metropolitan area (Navarra, Spain). Patients were enrolled between July 31, 2020 and September 11, 2020 and randomized in a 1:1 ratio to ivermectin (400 mcg/kg) single oral dose or placebo. The randomization sequence was computer-generated by the trial statistician using blocks of four to ensure balance. Allocation was made by the attending investigator using opaque envelopes. The placebo tablets did not match ivermectin in appearance, therefore, in order for the clinical trial team to remain blinded, treatment was administered under direct supervision by a non-participant nurse that picked up the opaque bottles directly from the pharmacy and administered the content behind closed doors. The clinical trial team had no contact with the investigational products. There was slow recruitment due to a sharp reduction in local transmission for 10 weeks after the lockdown of March-April 2020, the protocol was amended on September 2nd to extend the inclusion criteria from 48 to a maximum of 72 h of cough or fever.

The main objective was to determine the efficacy of a single dose of ivermectin, administered to low risk, non-severe COVID-19 patients in the first 72 h after fever or cough onset to reduce onward transmission.

### 2.3 Clinical, laboratory and virological monitoring

Assessments on enrollment and at days 4, 7, 14, 21 and 28 post treatment included: general symptoms report, physical examination (including respiratory rate, blood oxygen saturation and chest auscultation) and adverse events. All patients were asked to complete a daily online diary of symptoms from day 1 to 28 post treatment. On enrollment, as well as on days 7 and 14 blood samples were obtained to assess full blood count, C reactive protein, procalcitonin, ferritin, creatinine phosphokinase, lactic dehydrogenase, troponin T, D dimer, IL-6, and renal function.

A nasopharyngeal swab for SARS-CoV-2 PCR was taken at enrollment and on days 4, 7, 14 and 21 post treatment. For consistency, these samples were collected by three clinicians using the same technique. All samples were processed by PCR for genes N and E of SARS-CoV-2 (Real Time PCR SARS-CoV-2, Vircell SLU, Granada, Spain). For every sample, the viral load was calculated using standard reference curve (EDX Sars-Cov-2, Exact Diagnostics LLC, Fort Worth Texas). Additionally, all samples from day 4 post treatment were cultured in Vero cells for 7 days, after which the cytopathic effect was assessed and PCR conducted on the harvested cell-free supernatant. If the PCR from the supernatant was positive at day 4, the procedure was repeated on the samples of that patient for day 7. A semi-quantitative serology for IgG against SARS-CoV-2 (COVID-19 VIRCLIA IgG monotest, Vircell SLU, Granada, Spain) was done on samples from all patients on day 21 post-treatment.

### 2.4 Outcome measures

The primary outcome measure was the proportion of patients with detectable SARS-CoV-2 RNA by PCR from nasopharyngeal swab at day 7 post-treatment.

Relevant pre-specified secondary outcomes included viral load at days 4, 7, 14 and 21 post treatment; proportion of patients with symptoms (particularly fever and cough) at days 4, 7, 14 and 21 post-treatment as well as proportion of patients progressing to severe disease or death during the trial; proportion of patients with seroconversion at day 21 post-treatment and proportion of drug-related adverse events.

### 2.5 Sample size justification

In COVID-19, viral load peaks right before or at symptom onset [16,17] and most secondary cases occur prior to day five after symptoms [18]. This pilot was designed to assess the use of ivermectin to reduce transmission. With the objective to reduce onward transmission, a robust effect size in the proportion of PCR positives at day seven after treatment would be needed to have a public health impact. A reduction of at least 50% in the proportion of positives was considered of potential value.

The sample size was based in the comparison of two proportions and calculated to have 80% power at a 5% significance level to detect a 50% reduction (100 vs 50%) in the proportion of participants with positive PCR at day 7 post-treatment. The 100% PCR positivity figure at day 7 is based on the experience with COVID-19 outpatients at the Clínica Universidad de Navarra during the first wave of March-May 2020. The infectivity outcome was supported by assessing changes in viral load and infectivity in cell cultures.

### 2.6 Statistical analysis

Descriptive analyses used frequency and percentage (based on the non-missing sample size) for qualitative variables and median, interquartile range and n (non-missing sample size) for quantitative variables.

For the primary objective, the proportion of participants with positive PCR at day seven post treatment was calculated. Proportions were compared between study arms using Fisher's exact test and presented as a relative risk ratio (RR) with their corresponding 95% confidence interval (CI). In the analysis of the symptoms reported by patients (symptom diary), missing data was carried over from the last data available. Significance was set at 0.05. The analysis was carried out using Stata (StataCorp. 2019. Stata Statistical Software: Release 16. College Station, TX: StataCorp LLC).

Boxplots and bar plots were produced for the description of quantitative and qualitative variables, respectively. For figure readability, viral load values were log-transformed. Graphs were produced in R version 4.0.2 (R Core Team, R: A Language and Environment for Statistical Computing, Vienna, Austria: R Foundation for Statistical Computing, 2020) with the package ggplot2 (H. Wickham, ggplot2: Elegant Graphics for Data Analysis, Springer-Verlag New York, 2016.).

Viral load data were synchronized prior to analysis by accounting for days since onset of any symptoms and, since the day of infection was not known, an average incubation time of 5 days was assumed [19]. Peak viral load (Cmax) and time to peak viral load (Tmax) were determined directly from the profiles. Area under the viral load curve was calculated using the trapezoidal rule from assumed time of infection to last sample (AUCobs). Duration of time above a cycle threshold (Ct) of 35 was derived directly from profiles or linearly extrapolated profiles if the last recorded Ct value was not below the threshold.

### 2.7 Post hoc analyses

The median viral load at all sampling times and median IgG titers between study groups were compared using a Wilcoxon rank-sum test.

The effect of study arm on the presence of symptoms was estimated using mixed effect logistic regression models with subject as a random intercept. These models are adjusted by day of follow up (as symptoms are expected to disappear over time) and duration of symptoms before enrolment (as a proxy of disease onset). To assess the potential effect of study arm on symptom progression, the interaction between study arm and day of follow up was also included in the models. Three models were studied for those outcomes for which differences between treatments were observed: any symptom, anosmia or hyposmia, and cough. Additionally, the observed effect of ivermectin on anosmia/hyposmia was assessed in a sub-analysis by sex.

The adipose weight of participants in the ivermectin group was calculated with the method described by Gomez-Ambrosi et al. [20]. This information was used to estimate the ivermectin dose per adipose-weight received and plotted against the last day of reported anosmia/hyposmia.

## 3 Results

### 3.1 Patient characteristics

**Fig. 1:**
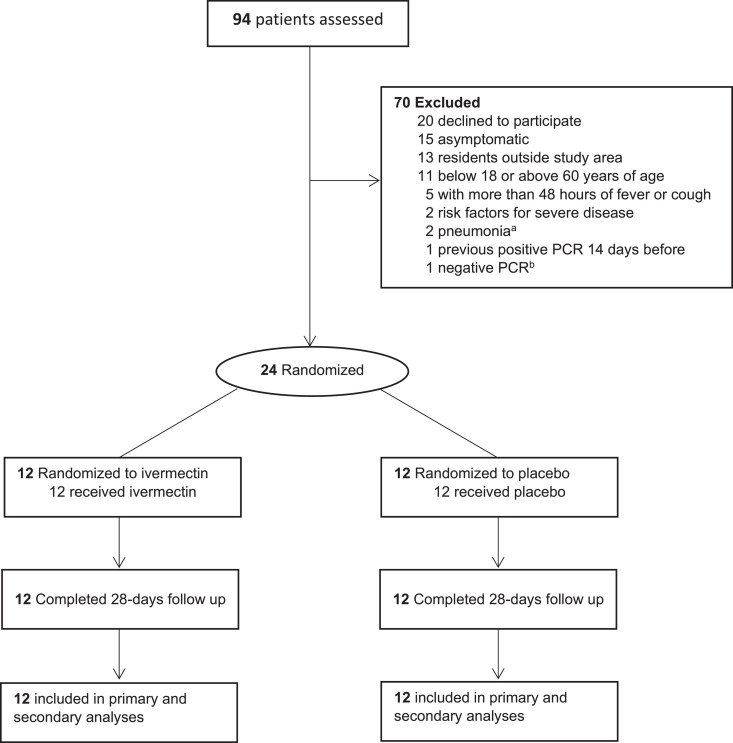
Enrollment and patient flow. ^a^ One presented with pneumonia in the ER and one had a compatible X-ray during screening. ^b^ Formally screened based on epidemiological and clinical suspicion but had a negative PCR.

Of 94 patients assessed, 50 did not meet eligibility criteria, 20 declined to participate and 24 were randomized. All randomized patients received the corresponding study product and completed 28 days of follow-up (Fig. 1). The baseline characteristics of patients in both groups are presented in Table 1.

**Table 1:** Baseline characteristics of patients by group.

	Ivermectin (*n* = 12)	Placebo (*n* = 12)
**Age, median (IQR)[range] (years)**	26 (19–36) [18–54]	26 (21–44) [18–54]
**Sex, No. (%)**		
Female	5 (42%)	7 (58%)
Male	7 (58%)	5 (42%)
Body mass index, median (IQR) [range] kg/m^2^	23·5 (19·6–27·8) [18·6–29· 9]	22·9 (21·0–24·8) [19·3–29·9]
**Symptoms**		
Any, No. (%)	12 (100%)	12 (100%)
Fever, No. (%)	7 (58%)	9 (75%)
Cough, No. (%)	4 (33%)	2 (17%)
Headache, No. (%)	7 (58%)	10 (83%)
Myalgia/general malaise, No. (%)	8 (67%)	6 (50%)
Earliest start of any symptom^a^, median, (IQR) [range]	24 (24–48) [18–120]	48 (36–48) [24–72]
Earliest start of fever^a^*, No, median. (IQR) [range]	24 (12–24) [12–24], *n* = 7	24 (24–48) [4–48], *n* = 9
Earliest start of cough^a^, No, median. (IQR) [range]	24 (16–36) [8–48], *n* = 4	10 (8–12) [8–12], *n* = 2
**Vital signs**		
Systolic Blood pressure, median. (IQR), mmHg	114 (113–117)	129 (116–134)^b^
Diastolic blood pressure, median. (IQR), mmHg	76 (72–80)	79 (77–85)
Heart rate, median (IQR), bpm	83 (77–99)	90 (81–100)
Respiratory rate, median (IQR), bpm	14 (12–17)	14 (12–15)
Temperature, median (IQR), °C	36·8 (36·4–37·0)	36·9 (36·5–37·0)
Oxygen saturation, median (IQR),%	97 (96–98)	98 (97–100)
**Viral load**		
Gene E, No. (IQR), copies/ml	1·7·10^7^ (5·9·10^6^- 3·9·10^8^)	2·7·10^7^ (8·3·10^5^- 4·2·10^8^)
Gene N, No. (IQR), copies/ml	3·7·10^8^ (1·8·10^7^- 9·3·10^9^)	3·3·10^8^ (5·8·10^7^- 6·7·10^9^)
**Inflammatory markers**		
CRP, median (IQR), mg/dL [normal value]	0·3 (0·2–0·8) [<0·5]	0·3 (0·2–0·6) [<0·5]
Ferritin, median (IQR), mg/dL [normal value]	165·0 (95·8 - 241·3) [30–400]	156·1 (103·1–223·0) [30–400]
IL-6, median (IQR), pg/mL [normal value]	6·5 (5·1 - 9·6) [<7]	4·5 (3·0–6·5) [<7]
D-Dimer, median (IQR), ng/mL [normal value]	295 (270–420) [150–500]	280 (270–315) [150–500]
Full blood count		
Red blood cells, median (IQR), 10^12^/L	5·05 (4·62–5·55)	5·07 (4·67– 5·45)
Hemoglobin, median (IQR), g/dL	15·3 (13·8–16·0)	15·2 (13·7–15·8)
Platelets, median (IQR), 10^9^/L	194 (167–216)	205 (179–247)
White blood cells, median (IQR), 10^9^/L	4·7 (4·3–6·3)	4·4 (3·7–5·9)
Neutrophils, median (IQR),%	52·4 (45·6–65·1)	53·4 (43·9–62·2)
Lymphocytes, median (IQR),%	29·5 (18·5- 7·9)	28·7 (20·8–39·9)

a Hours before dosing

b The slightly higher median systolic blood pressure in the placebo group at baseline was not seen in subsequent study visits and was judged as non-clinically significant, see table S3 for the evolution of all vital signs throughout the study, *Reported or measured fever. IQR: interquartile range

There was a higher proportion of females in the placebo group (58 vs 42%). Demographics and baseline disease characteristics of participants in both groups are presented in Table 1. Overall, 66% of the patients presented with perceived or objective fever, 25% presented with cough, 70% presented with headache and 58% presented with myalgia or general malaise with no remarkable differences between groups. The median earliest start of any symptom before treatment was 24 h for the ivermectin group (interquartile range, 24–48 h) and 48 h for the placebo group (interquartile range, 36–48 h). At baseline, there were no differences in vital signs, inflammatory markers or full blood count between the groups (Table 1).

### 3.2 Primary endpoint

There was no difference in the proportion of PCR positive patients at day 7 post treatment, 12/12 (100%) patients had a positive PCR for gene N in both groups. For gene E, 11/12 (91%) in the ivermectin and 12/12 (100%) in the placebo group had a positive PCR (RR 0·92, 95% CI: 0·77–1 0·09, *p* = 1·0).

### 3.3 Viral load

**Fig. 2:**
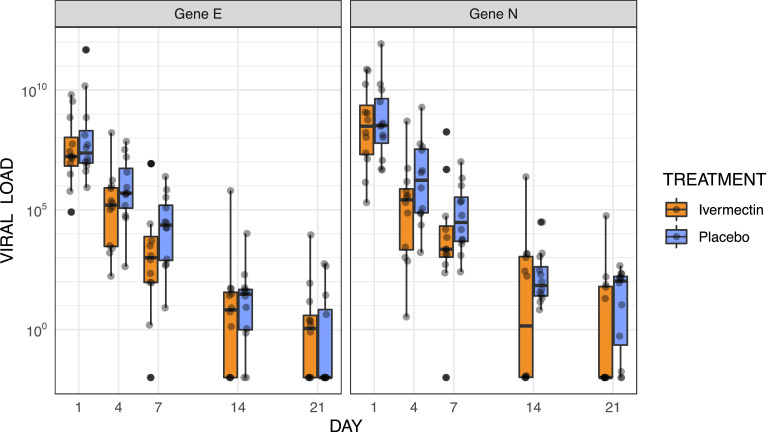
Viral load evolution by study arm. Viral load values were log-transformed. The boxes show the interquartile range. Dots represent each individual value.

Genes E and N had comparable results at all time points. Patients in both study groups had similar viral load before treatment with median and interquartile range for genes E and N in the same orders of magnitude (Fig. 2 and Table S1). Although there was a consistent overlap in interquartile ranges and full ranges at all points, the median viral load for both genes was lower at days 4 and 7 post treatment in the ivermectin group with differences increasing from 3-fold lower at day 4 (*p* = 0·24 for gene E; *p* = 0·18 for gene N) to around 18-fold lower at day 7 (*p* = 0·16 for gene E; *p* = 0·18 for gene N) (Fig. 2 and Table S1). A similar tendency remained for the viral load at days 14 and 21, with values from patients in the ivermectin group consistently lower for at least one of the genes, the difference was not statistically significant at any single point (Fig. 2 and Table S1). The values of cycle thresholds had a very similar behavior (Figure S1). Summary statistics for viral kinetics are provided in Table S2.

### 3.4 Viral culture

At day 4 post-treatment, 7/12 samples in the ivermectin and 5/12 samples in the placebo group effectively replicated Vero cell culture; the median Ct values of replicating samples were 23·3 and 23·8 for genes N and E respectively, while the median Ct values of non-replicating samples were 27·6 and 27·9 for genes N and E respectively. By day 7 post treatment only 1/6 in the ivermectin (one previously positive sample was lost) and 1/5 in the placebo group replicated in the cell culture; the median Ct values of replicating samples were 25·1 and 26·0 for genes N and E respectively, while the median Ct values of non-replicating samples were 30·8 and 32·0 for genes N and E respectively.

### 3.5 Symptoms

There was good compliance with the daily online questionnaire with 282 patient-days reports (84%) and 295 patient-days reports (88%) in the ivermectin and placebo group respectively (Fig. S2).

**Fig. 3:**
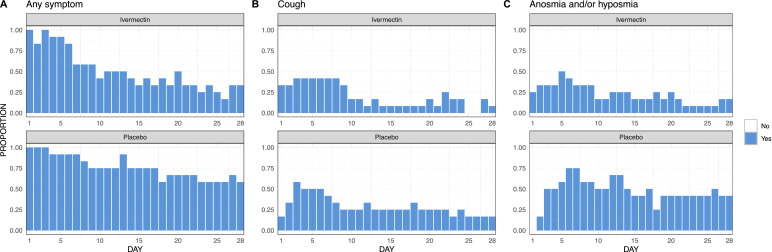
Daily proportion of any self-reported symptoms, self-reported cough and self-reported anosmia/hyposmia by study arm. Each graph represents the daily proportion of individuals (n/N) who suffered from each symptom in the corresponding study arm for a 28 day follow up. Missing answers were replaced by the value in the immediately preceding day.

Patients in the ivermectin group reported fewer patient-days of any symptoms than those in the placebo group (171 vs 255 patient-days). This difference is mostly driven by two symptoms, anosmia/hyposmia and cough. Patients in the ivermectin group reported 50% less anosmia/hyposmia than those in the placebo group (76 vs 158 patient-days of anosmia/hyposmia). The ivermectin group also reported 30% less cough (68 vs 97 patient-days of cough) (Fig. 3).

There were no major differences between ivermectin and placebo in the reported patient-days of fever (12 vs 12), general malaise (51 vs 61), headache (34 vs 38), or nasal congestion (91 vs 97). With much lower magnitudes, the ivermectin group reported 3.5-fold more patient-days of gastrointestinal symptoms (21 vs 6) and 5-fold less shortness of breath (3 vs 15) (Fig. S3).

No patient from either group progressed to severe disease.

### 3.6 Serology

**Fig. 4:**
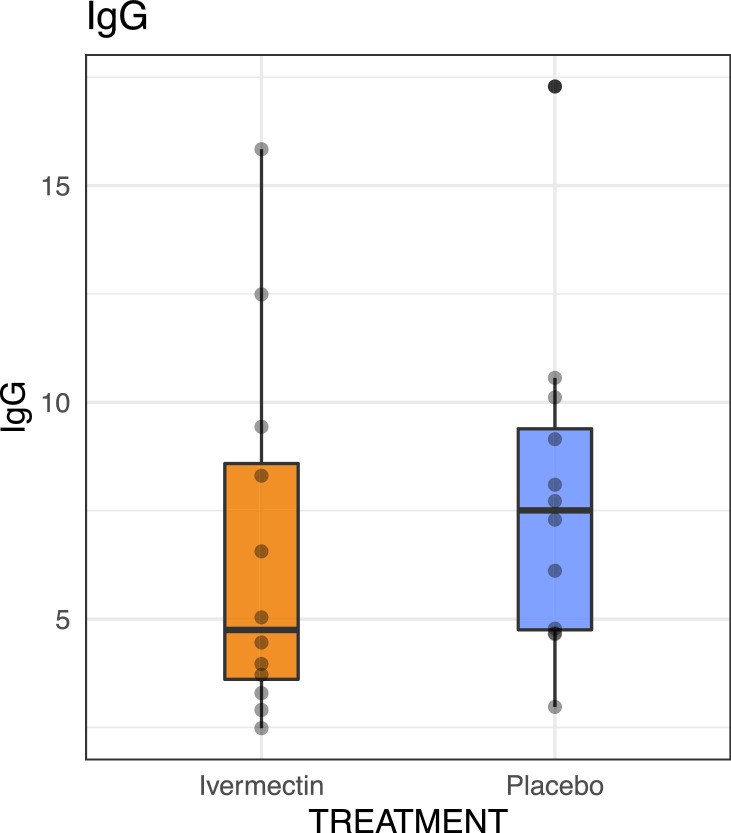
IgG titers by study arm. The boxes show the interquartile range. Dots represent each individual value (*p* = 0·24, Wilcoxon rank-sum test).

All patients in both groups seroconverted by day 21 post treatment. Patients in the ivermectin group had a lower median of IgG titers (Index 4·7, interquartile range [3·5–8·9]) than those in the placebo group (Index 7·5, interquartile range [4·2–9·3]) (*p* = 0·24 by Wilcoxon rank-sum test) (Fig. 4).

### 3.7 Safety

All patients completed the follow up period of 28 days. There were 15 adverse events (7 in the ivermectin and 8 in the placebo group) experienced by 10 patients (5 in the ivermectin and 5 in the placebo group). There were no severe adverse events.

The online diary of symptoms included questions about ivermectin-specific adverse events. There were no differences in the reported patient-days between the ivermectin and the placebo group for confusion (1 vs 0), drowsiness (0 vs 0), or pruritus (0 vs 3). Patients in the ivermectin group reported more patient-days of dizziness (7 vs 1) and blurred vision (24 vs 1), with this last value driven by a single patient in the ivermectin group reporting blurred vision on days 2—28, further evaluation suggested previously undiagnosed presbyopia (Fig. S4).

There were no major differences in the evolution of vital signs (Table S3), inflammatory markers (C reactive protein, procalcitonin, ferritin and IL-6) and rest of laboratory parameters of patients in each group (Table S4).

### 3.8 Post hoc analyses

**Fig. 5:**
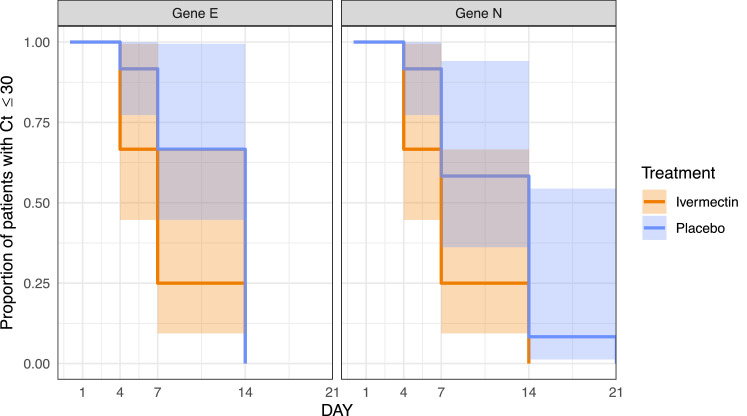
Kaplan-Meier curves for viral load. A survival threshold of Ct ≥ 30 was used. Log-Rank test yielded significance for the difference in gene E (*p* = 0·0358) and borderline significance for the difference in gene N (*p* = 0·0550).

Given that the main objective of the trial was to explore a reduction in onward transmission of the virus and that the viral cultures of samples from day 7 showed replicative virus only in samples with Ct values below 30, Kaplan-Meier curves were drawn and survival analysis conducted with log-rank test using a survival threshold of Ct ≥ 30. This analysis shows a statistically-significant difference for gene E (*p* = 0·035, Log-rank test) and borderline significance for gene N (*p* = 0·055, Log-rank test). The curves are presented in Fig. 5.

In the logistic regression model, a lower chance of presenting any symptoms was observed in the ivermectin arm (OR: 0·04 [95% CI: 0·00, 0·75] *p* = 0·032). In both arms, presence of any symptoms tended to diminish over time (OR (95% CI): 0·80 (0·74, 0·86) and 0·81 (0·77, 0·85), for placebo and ivermectin respectively). This downward trend was similar in both study groups (*p* = 0·687). With regards to the presence of cough, we did not find differences between study groups (*p* = 0·575) nor in the existing trend to diminish over time (*p* = 0·373). However, differences were observed for the presence of anosmia/hyposmia: for patients in the placebo group, there was no trend in time in the presence of anosmia (OR (95%CI): 0·99 (0·95, 1·02) *p* = 0·459). Conversely, patients in the ivermectin group showed a significant decrease (OR (95%CI): 0·90 (0·85, 0 0·94) *p* < 0·001) (Fig. S5).

The overall effect of ivermectin on anosmia/hyposmia was mainly driven by male patients (20 vs 76 patient-days of anosmia/hyposmia in the ivermectin and placebo groups respectively) in sharp contrast with female patients (56 vs 81 patient-days of anosmia/hyposmia in the ivermectin and placebo groups respectively) (Fig. S6). A sensitivity analysis to assess if sex had any impact on the logistic regression models was performed. Adding this variable to the models did not change the coefficients presented above.

The participants had an adipose weight ranging from 14% to 39% of their body weight. This resulted in ivermectin doses per adipose kilo ranging from 1028 mcg/adipose-kg to 2963 mcg/adipose-kg, even if the actual doses per full body weight were achieved in the relative narrow range of 399–427 mcg/kg (Table 2). The ivermectin doses per adipose-kg were plotted against the duration of anosmia/hyposmia and the last day of reported anosmia/hyposmia with no evident pattern, a regression was not attempted given the scarcity of data (Fig. S7).

**Table 2:** Body composition and dose of ivermectin-treated participants.

Participant ID	Age	Sex	Weight	Height	BMI	Adipose weigh	Adipose weight*	3-mg tablets received	Total dose	Dose/kg	Dose adipose/kg
	years	M/F	kg	m	kg/m^2^	%	kg	tablets	mg	mcg/kg	mcg/kg
SAINT-5	24	Male	65·1	1·77	20·8	0·14	9·11	9	27	415	2963
SAINT-17	22	Male	72·7	1·79	22·7	0·17	12·43	10	30	413	2413
SAINT-18	18	Female	57·0	1·75	18·6	0·21	11·97	8	24	421	2005
SAINT-19	18	Female	45·1	1·55	18·8	0·21	9·61	6	18	399	1874
SAINT-3	33	Male	71·0	1·70	24·6	0·21	15·48	10	30	423	1938
SAINT-21	18	Female	50·1	1·60	19·6	0·22	11·47	7	21	419	1830
SAINT-16	20	Female	49·2	1·59	19·5	0·23	11·37	7	21	427	1848
SAINT-9	28	Male	89·9	1·79	28·1	0·26	23·82	12	36	400	1511
SAINT-11	47	Male	85·8	1·77	27·4	0·27	23·77	12	36	420	1515
SAINT-20	29	Male	92·2	1·79	28·8	0·27	25·54	13	39	423	1527
SAINT-8	39	Male	93·6	1·77	29·9	0·30	28·27	13	39	417	1380
SAINT-13	57	Female	66·0	1·57	26·6	0·39	26·27	9	27	409	1028

## 4 Discussion

In spite of its partial antiviral properties, ivermectin received limited early attention in Europe or the US as a potential drug to be repurposed against COVID-19. This was largely based on one pharmacokinetic model stressing the inability of currently approved oral doses to reach lung tissue levels at the antiviral concentrations described by Caly et al., [21] even if other, peer-reviewed models predict up to 10-fold accumulation of ivermectin in target tissue [22]. There are additional reasons to avoid direct inferences from the results of in vitro experiments or pharmacokinetic models, these include the potential role of ivermectin metabolites, the potential immunomodulatory role of the drug, and questions about the virus/cell ratios and appropriateness of the Vero cellular lines used in the cultures [23].

This pilot study was designed to assess the question of whether further investments in the potential repurposing of ivermectin were warranted. As such, we aimed at generating evidence on viral kinetics, antibody response and clinical efficacy in a cohort of patients at low risk of severe disease. Without a clearly defined mechanism of action, a sole signal in any of said parameters would not suffice to justify further efforts. This pilot shows a tendency to lower viral loads in the ivermectin group, a tendency to lower IgG titers that may reflect milder disease and clinical benefit in cardinal symptoms of COVID-19 associated with tissue damage: anosmia/hyposmia and cough. These results are in line with emerging evidence from trials in Bangladesh [10,11] and Argentina [12] showing a faster viral clearance in treated participants, as well as with recent data from a SARS-CoV-2 hamster model from Institute Pasteur which also showed a marked sex dichotomy in the effect of ivermectin on anosmia/hyposmia [24].

Pending confirmation of these results, this pilot sheds some light on the potential mechanism of action of ivermectin against COVID-19. Note the trial was not powered to detect modest differences in viral load, yet a small effect is suggested when viral load was ascertained directly by PCR and indirectly using IgG titers as markers of disease severity [25,26]. Also, in this pilot ivermectin has not shortened the duration of symptoms associated with systemic inflammation such as fever or malaise, nor has it had a measurable impact on systemic inflammatory markers.

Given these findings, consideration could be given to alternative mechanisms of action different from a direct antiviral effect. One alternative explanation might be a positive allosteric modulation of the nicotinic acetylcholine receptor caused by ivermectin and leading to a downregulation of the ACE-2 receptor and viral entry into the cells of the respiratory epithelium and olfactory bulb [27].

Another mechanism through which ivermectin might influence the reversal of anosmia is by inhibiting the activation of pro-inflammatory pathways in the olfactory epithelium. Inflammation of the olfactory mucosa is thought to play a key role in the development of anosmia in SARS-CoV-2 infection [28].

Ivermectin is known to downregulate the expression of several pro-inflammatory genes, including those of IL-8, TNF-α, and cathelicidin LL-37 [29]. This effect is thought to partially explain the efficacy of ivermectin in the treatment of rosacea [29,30]. The effect on LL-37 might be particularly important, as this molecule directly influences several pro and anti-inflammatory pathways, including the stimulation of IL-18 and IL-1β production, and has a chemotactic effect for neutrophils and eosinophils [31]. This effect might be mediated by inhibiting the entrance of the vitamin D receptor (VDR) into the nucleus [32]. Ivermectin inhibits importins of the α/β family, which play a key role in the ligand-independent transportation of the VDR, a crucial step in the vitamin d-mediated expression of the hCAMP18 gene, which encodes the LL-37 precursor [33], [34], [35]. It is possible that the inhibition of importins may thus contribute to the immune regulatory effect of ivermectin, and its influence on other vitamin d-mediated pathways, supporting further studies in this area.

Albeit requiring confirmation, these results raise several important questions. If the mechanism of action of ivermectin against COVID-19 is related to a nicotinic effect, then inhibitory concentrations for this receptor (which are in the nanomolar range) could be achievable in the lung tissue for a short period of time with oral dosing and for considerably longer periods with nebulized therapy [36]. If the mechanism is immunomodulatory, then the appropriate dose and regimen should be tailored accordingly. Before considering higher or multiple dose schemes, there is also need to better understand the potential role of ivermectin´s metabolites in any observed effect. Finally, given the tendency to lower IgG titers in the ivermectin group, there is need to evaluate the potential relationship between ivermectin treatment, disease severity, inflammation, viral dynamics and antibody titers; [37,38] particular attention should be paid to the long-term humoral and cellular immune responses against SARS-CoV-2 in ivermectin treated patients.

This pilot points towards a potential use of ivermectin in COVID-19 which warrants further exploration under larger trials, with clinical outcomes in patients with risk factors or more severe disease. This is of particular importance for settings with limited resources given ivermectin´s low price, broad availability and scalability of manufacturing processes.

This pilot has several key limitations that warrant careful interpretation of the results. Firstly, it was designed to explore a potential signal for the use of ivermectin in COVID-19, not to provide definitive evidence on the subject, hence its small sample size. Second this pilot was restricted to subjects with non-severe disease and no risk factors in whom the treatment was provided in the first 48 h of fever or cough, this should be taken into consideration for the design of any confirmatory studies to be conducted. Additionally, the quantification of the viral load presented is intrinsically limited by heterogeneity in the samples, even if all were obtained by the same clinicians, standardization against a human epithelial cell gene would be required to ensure the viral loads are truly comparable [39].

The positive signal found in this pilot warrants the conduction of larger trials using ivermectin for the early treatment of COVID-19. Such trials should include patients with risk factors for severe disease as well as patients with pneumonia. The potential for a mechanism of action different to direct antiviral effect also opens the door for pre-exposure prophylaxis in high-risk groups.

## Author contributions

Carlos Chaccour and Aina Casellas had full access to all of the data in the study and take responsibility for the integrity of the data and the accuracy of the data analysis.

Conceptualization: CCh, NRR, MFA

Data curation: CCh, AC, JB

Formal analysis: AC, CCh, FH, VS

Funding acquisition: CCh, NRR

Investigation: CCh, AB, IP, AFM, PRC, MAR, MRM, CJI, FC, MG, EL, JCG, JRY, JLD, GR, BS, MFA

Methodology: CCh, AC, FC, CD, GM, FH, GR, BS, MFA

Supervision: CCh, BS, GR, MFA

Writing - original draft: CCh, AC

Writing - review & editing: all authors contributed, reviewed and approved the last draft.

## Funding

Idipharma SL (Noain, Spain) contributed with in kind placebo tablets. This study was supported by ISGlobal and the University of Navarra. CCh, PRC, MAR, FH and NRR received salary support from Unitaid through the BOHEMIA grant to ISGlobal. ISGlobal acknowledges support from the Spanish Ministry of Science and Innovation through the “Centro de Excelencia Severo Ochoa 2019–2023”; Program (CEX2018–000,806-S), and support from the Generalitat de Catalunya through the CERCA Program.

## Data availability

Upon publication, all data supporting the results will be archived in a public repository accessible at http://diposit.ub.edu/dspace/handle/2445/101776

## Declaration of Competing Interest

JLDP reports speaker fees from Pfizer and MSD as well as research grants from Novartis, outside the scope of the submitted work. No other competing interests were disclosed

## Appendix Supplementary materials

CONSORT Checklist

Supplementary tables and figures

Study protocol

Statistical analysis plan
