# Supplementary material for: The effect of early treatment with ivermectin on viral load, symptoms and humoral response in patients with non-severe COVID-19: A pilot, double-blind, placebo-controlled, randomized clinical trial
Source: eClinicalMedicine. 2021 Jan 19;32:100720. doi: 10.1016/j.eclinm.2020.100720 (PMC7816625; doi:10.1016/j.eclinm.2020.100720)
Supplement: Supplementary file 2 [file mmc2.pdf]

The effect of early treatment with ivermectin on viral load, symptoms and humoral response in patients with mild COVID-19: a pilot, double-blind, placebo-controlled, randomized clinical trial.

Table S1. Viral loads during follow up by study arm 2
Figure S1: Cycle threshold evolution 3
Figure S2: Compliance with symptoms diary 4
Table S2: Summary statistics of viral load profiles 5
Figure S3: Reported fever, headache, general malaise, nasal congestion, gastrointestinal, shortness of breath . 6
Figure S4: Reports of symptoms potentially associated with ivermectin 7
Table S3. Vital signs during visits by study arm 8
Table S4. Laboratory parameters during follow up by study arm 9
Figure S5: Symptoms' predicted probability by study arm. 10
Figure S6: Anosmia/hyposmia by sex and treatment group 11
Figure S7: Ivermectin dose per adipose weight and anosmia/hyposmia 12

Table S1. Viral loads during follow up by study arm

	Day				
	**1**	**4**	**7**	**14**	**21**
**Viral load (gene E)**					
Ivermectin	$1.7 \cdot 10^7$ ($5.9 \cdot 10^6$- $3.9 \cdot 10^8$)	$1.6 \cdot 10^5$ (2820-$8.8 \cdot 10^5$)	1018 (92-15445)	7 (0- 42)	1 (0- 9)
Placebo	$2.7 \cdot 10^7$ ($8.3 \cdot 10^5$- $4.2 \cdot 10^8$)	$4.9 \cdot 10^5$ ($1.0 \cdot 10^5$-$9.9 \cdot 10^6$)	23550 (709-$2.3 \cdot 10^5$)	30 (1- 50)	0 (0- 16)
p-values*	0.6442	0.2482	0.1659	0.4154	0.4888
**Cycle threshold (gene E)**					
Ivermectin	20 (17- 22)	27 (25- 31)	33 (30- 35)	36 (35- 41)	38 (36- 41)
Placebo	21 (18- 23)	26 (22- 28)	30 (28- 32)	35 (35- 37)	41 (37- 41)
p-values*	0.6861	0.2040	0.1659	0.2218	0.2846
**Viral load (gene N)**					
Ivermectin	$3.7 \cdot 10^8$ ($1.8 \cdot 10^7$- $9.3 \cdot 10^9$)	$2.7 \cdot 10^5$ (1885-$1.0 \cdot 10^6$)	2255 (938-34650)	86 (0- 1235)	0 (0- 67)
Placebo	$3.3 \cdot 10^8$ ($5.8 \cdot 10^7$- $6.7 \cdot 10^9$)	$2.2 \cdot 10^6$ (73150-$3.7 \cdot 10^7$)	36800 (4510-$6.3 \cdot 10^5$)	75 (24- 710)	107 (0- 183)
p-values*	1.0000	0.1842	0.1842	0.3552	0.0855
**Cycle threshold (gene N)**					
Ivermectin	20 (16- 22)	27 (24- 31)	32 (30- 34)	36 (33- 41)	41 (35- 41)
Placebo	21 (17- 22)	25 (22- 27)	29 (27- 31)	35 (33- 35)	35 (35- 39)
p-values*	0.6442	0.1333	0.1060	0.2982	0.2597

Number of subjects per day and study arm is 12. IQR: interquartile range. *Wilcoxon rank-sum test

Figure S1: Cycle threshold evolution

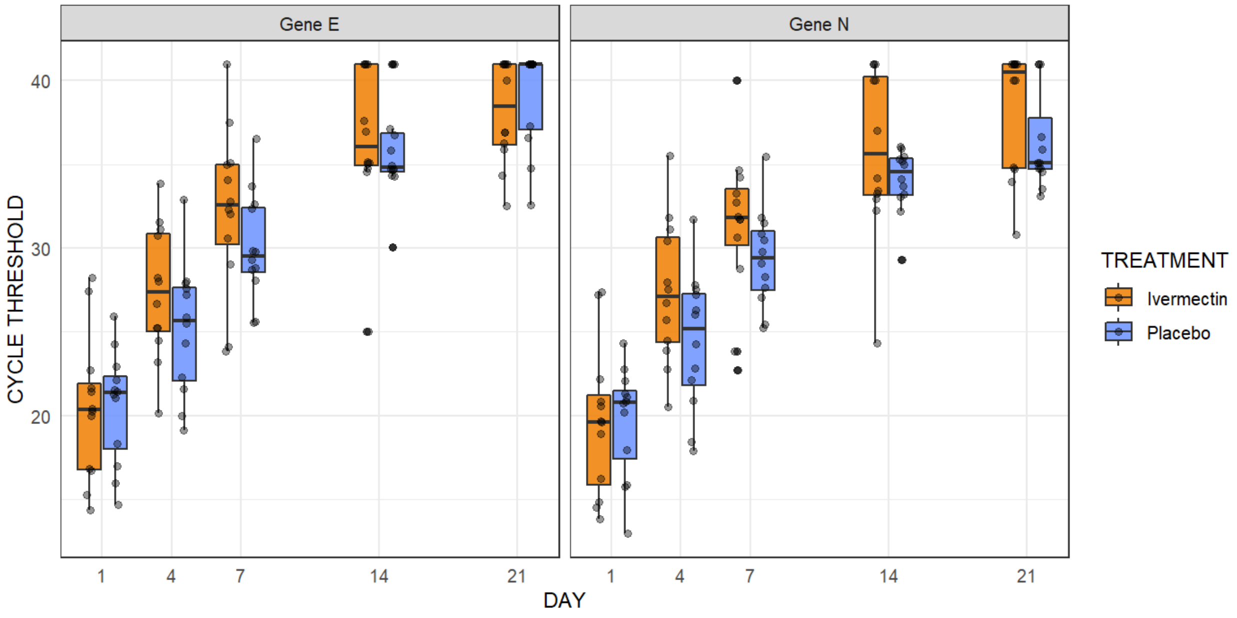

The boxes show the median and interquartile range (IQR). The whiskers extend +/-1.5 times the IQR. Dots represent each individual value.

Figure S2: Compliance with symptoms diary

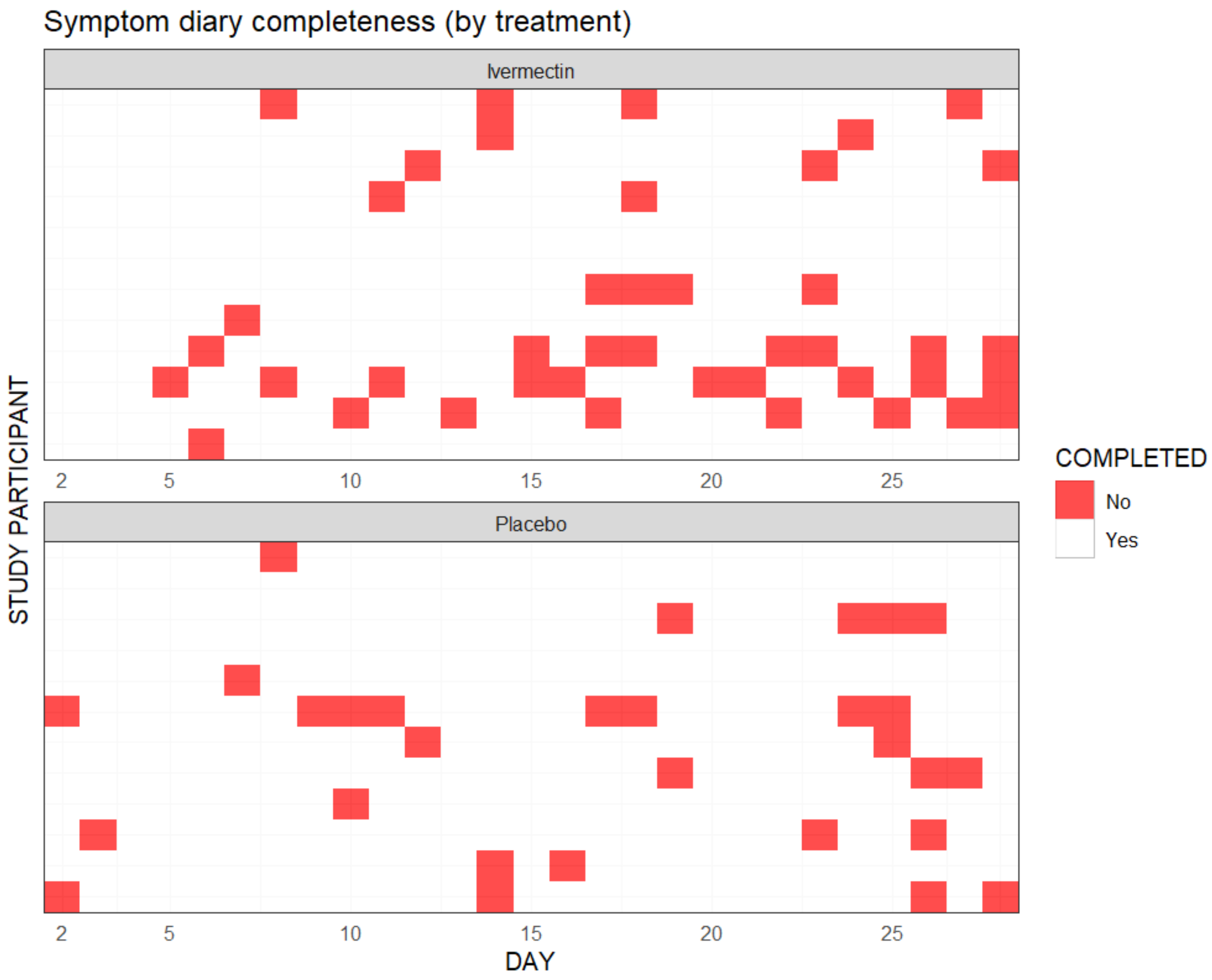

The symptoms diary had to be completed each day from days two to 28. Each row represents a study participant. Highlighted in red are those cases where the diary was not completed.

Table S2: Summary statistics of viral load profiles

	Ivermectin	Placebo	p value
**Peak viral load (Cmax), median (IQR) [range], cycles**	19.63 (15.87 - 21.19) [13.82 - 27.22]	20.48 (17.36 - 21.14) [12.94 - 22.77]	0.5846
**Time to peak viral load (Cmax), median (IQR) [range], days**	6.00 (6.00 - 7.00) [5.75-10.00]	7.00 (6.75 - 7.00) [6 - 11]	0.2637
**Time from start to end of positivity threshold of 35 cycles (duration), median (IQR) [range], days**	18.00 (12.80 - 21.05) [11 – 27.7]	20.10 (18.53 - 25.52) [11.8 - 28.9]	0.1017
**AUCobs, median (IQR) [range], cp/mL*d**	$3.36*10^{8}$ ($3.74*10^{7}$ - $1.9*10^{10}$) [$4.19*10^{5}$-$1.45*10^{11}$]	$8.24*10^{8}$ ($2.51*10^{8}$ - $1.02*10^{10}$) [$2.08*10^{7}$ - $1.70*10^{12}$]	0.4764

IQR: interquartile range; AUCobs: area under the viral load curve. Statistical significance was assessed using a two-tailed unpaired t test with (AUCobs) or without (Cmax duration) logarithmic transformation and the Wilcoxon rank sum test (Tmax).

Figure S3: Reported fever, headache, general malaise, nasal congestion, gastrointestinal, shortness of breath

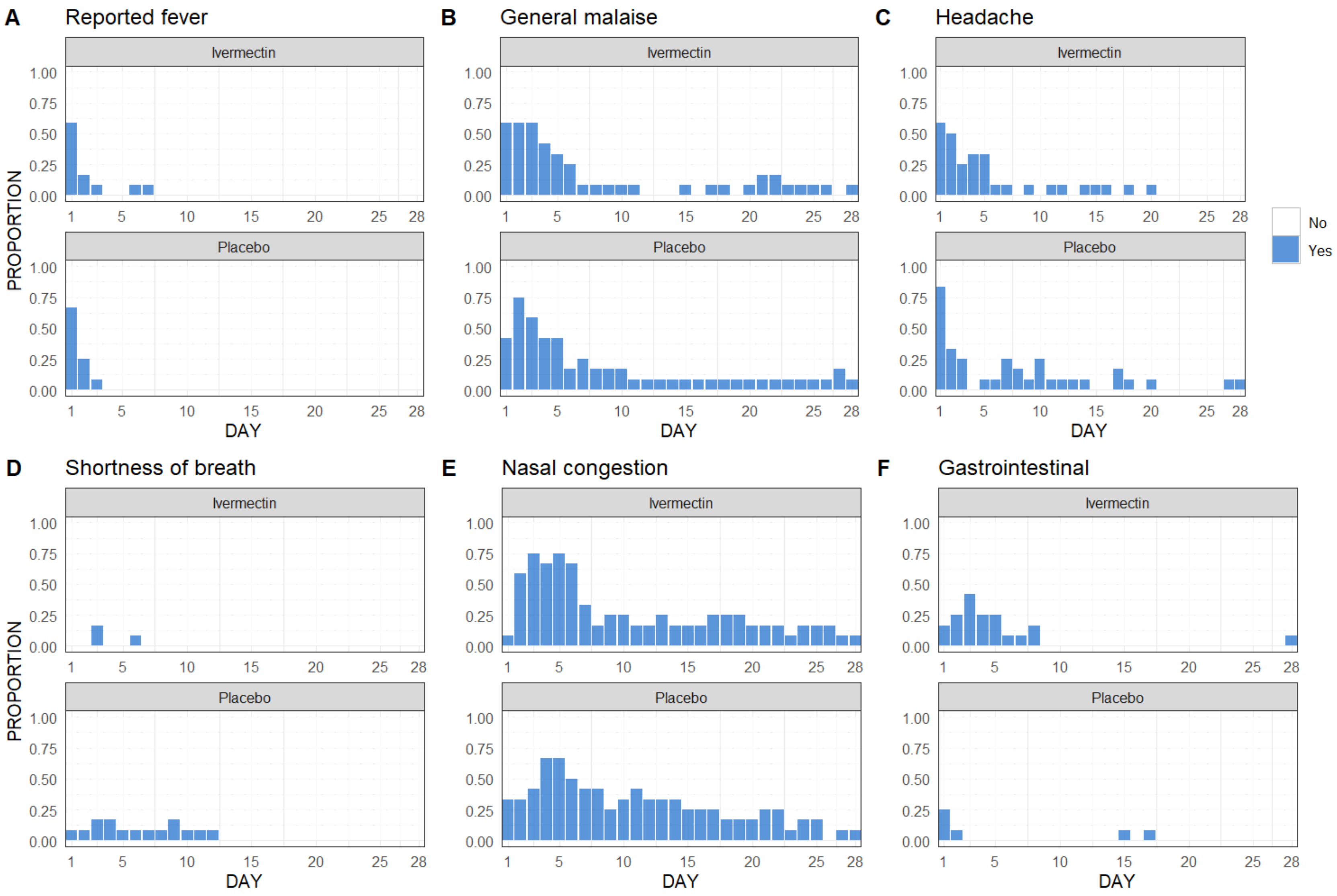

Each individual graph represents the daily proportion of individuals (n/N) who suffered from each symptom in the corresponding study arm for a 28 day follow up.

Figure S4: Reports of symptoms potentially associated with ivermectin

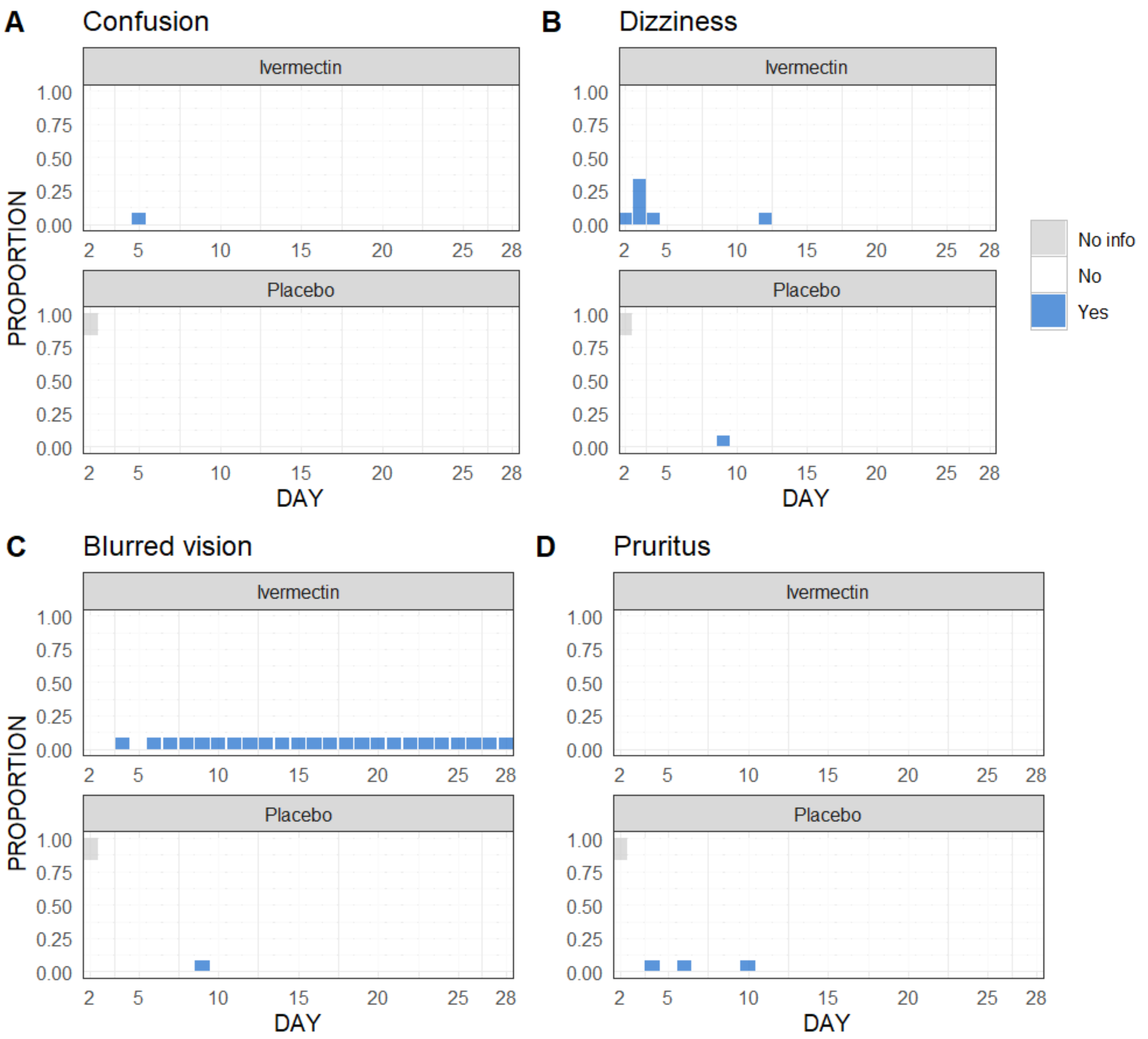

Each individual graph represents the daily proportion of individuals (n/N) who suffered from each potential side effect in the corresponding study arm for a 28 day follow up. Note that these data come from the symptom diary, which was completed from day two to day 28. Missing information was carried over from the last data available. There is some gray area on day two because there were two participants who did not complete the symptom diary on that day and no previous information existed.

Table S3. Vital signs during visits by study arm

	Day				
	**1**	**7**	**14**	**21**	**28**
**Systolic blood pressure, median (IQR), mmHg**					
Ivermectin	114 (113- 117)	108 (99- 116)	106 (102- 112)	112 (103- 122)	113 (108- 122)
Placebo	129 (116- 134)	119 (110- 123)	114 (108- 126)	114 (104- 121)	122 (117- 131)
**Diastolic blood pressure, median (IQR), mmHg**					
Ivermectin	76 (72- 80)	71 (66- 76)	69 (68- 72)	73 (70- 78)	75 (69- 78)
Placebo	79 (77- 85)	76 (72- 80)	73 (68- 78)	73 (70- 77)	79 (75- 85)
**Heart rate, median (IQR), bpm**					
Ivermectin	83 (77- 99)	75 (68- 77)	74 (67- 84)	84 (74- 91)	75 (65- 87)
Placebo	90 (81- 100)	78 (75- 94)	80 (72- 89)	83 (74- 93)	92 (76- 97)
**Respiratory rate, median (IQR), breaths per minute**					
Ivermectin	14 (12- 17) [11]	12 (12- 14)	14 (12- 15)	14 (12- 14)	12 (12- 14)
Placebo	14 (12- 15)	14 (12- 14)	14 (13- 15)	12 (12- 14)	14 (12- 14)
**Temperature, median (IQR), ºC**					
Ivermectin	36.8 (36.4-37.0)	36.4 (36.0-36.5)	36.4 (36.0-36.7)	36.5 (36.0-36.6)	36.5 (36.4-36.6)
Placebo	36.9 (36.5-37.0)	36.5 (36.2-36.7)	36.5 (36.3-36.6)	36.4 (36.2-36.8)	36.5 (36.1-36.9)
**Oxygen saturation, median (IQR), %**					
Ivermectin	97 (96- 98)	97 (96- 99)	97 (96- 97)	96 (96- 98)	97 (96- 98)
Placebo	98 (97- 100)	98 (97- 98)	98 (97- 98)	97 (96- 98)	97 (96- 98)

Number of subjects per day and study arm is 12, otherwise indicated in brackets.
IQR: interquartile range; bpm: beats per minute.

Table S4. Laboratory parameters during follow up by study arm

Variable	Day 1	Day 7	Day 14	Normal range
**Blood creatinine, median (IQR), mg/dL**				
Ivermectin	0·9 (0·8- 1·1)	0·8 (0·8- 0·9)	0·8 (0·8- 1·0)	0·7-1·2
Placebo	0·9 (0·6- 1·1)	0·8 (0·7- 1·0)	0·8 (0·7- 1·0) [11]	
**Urea blood, median (IQR), mg/dL**				
Ivermectin	28 (22- 34)	29 (27- 36)	30 (26- 35)	16·6-48·5
Placebo	27 (26- 35)	33 (27- 41)	30 (27- 38) [11]	
**LDH, median (IQR), Ul/L**				
Ivermectin	231 (192- 251) [11]	223 (202- 264)	235 (217- 250)	135-225
Placebo	217 (207- 255)	217 (191- 227)	224 (204- 232) [11]	
**CRP, median (IQR), mg/dL**				
Ivermectin	0·35 (0·16-0·81)	0·10 (0·03-0·16)	0·08 (0·06-0·10)	<0·5
Placebo	0·30 (0·19-0·60)	0·11 (0·06-0·21)	0·06 (0·04-0·12) [11]	
**Procalcitonin, median (IQR), ng/mL**				
Ivermectin	0·07 (0·04-0·10)	0·03 (0·02-0·03)	0·03 (0·02-0·03)	<0·5
Placebo	0·05 (0·04-0·06)	0·03 (0·02-0·03)	0·02 (0·02-0·04) [11]	
**Sodium, median (IQR), mEq/L**				
Ivermectin	139 (136- 142)	141 (140- 142)	141 (140- 141)	136-145
Placebo	139 (139- 141)	141 (140- 141)	141 (139- 143) [11]	
**Potassium, median (IQR), mEq/L**				
Ivermectin	4·1 (3·8- 4·1)	3·9 (3·8- 4·0)	4·0 (4·0- 4·1)	3·5-5·0
Placebo	4·1 (3·9- 4·3)	4·0 (3·8- 4·3)	3·9 (3·8- 4·2) [11]	
**Chlorine, median (IQR), mEq/L**				
Ivermectin	97 (95- 100)	100 (98- 102)	99 (98- 102)	98-107
Placebo	99 (98- 101)	100 (99- 101)	101 (98- 103) [11]	
**Bicarbonate, median (IQR), mEq/L**				
Ivermectin	24·2 (22·6-26·1)	24·8 (23·6-26·1) [11]	24·3 (22·6-25·4)	22-29
Placebo	24·5 (22·4-26·1)	24·9 (23·0-26·4)	24·5 (22·2-26·1) [11]	
**Remaining anion, median (IQR), mEq/L**				
Ivermectin	16·7 (15·2-19·3)	15·1 (14·7-19·8) [11]	15·8 (15·1-17·5)	8-16
Placebo	16·6 (12·7-18·1)	15·4 (14·7-17·4)	16·5 (13·5-19·2) [11]	
**Ferritin, median (IQR), ng/mL**				
Ivermectin	165·0 (95·8-241·3)	125·0 (79·5-343·4)	152·0 (69·4-305·3)	30-400
Placebo	156·1 (103·1-223·0)	198·7 (109·5-248·2)	145·2 (132·4-196·1) [11]	
**CPK, median (IQR), Ul/L**				
Ivermectin	98 (79- 131)	64 (47- 100)	106 (63- 160)	<190
Placebo	66 (59- 113)	47 (41- 76)	55 (52- 114) [11]	
**IL-6, median (IQR), pg/mL**				
Ivermectin	6·5 (5·1- 9·6)	4·3 (3·8- 5·2) [11]	2·7 (1·5- 4·4)	<7
Placebo	4·5 (3·0- 6·5) [11]	4·2 (3·5- 4·9) [11]	2·1 (1·5- 4·7) [11]	
**D-Dimer, median (IQR), ng/mL**				
Ivermectin	295 (270- 420)	295 (270- 435)	270 (270- 340)	150-500
Placebo	280 (270- 315)	270 (270- 310)	270 (270- 330) [11]	

Number of subjects per day and study arm is 12, otherwise indicated in brackets.
IQR: interquartile range.

Figure S5: Symptoms' predicted probability by study arm.

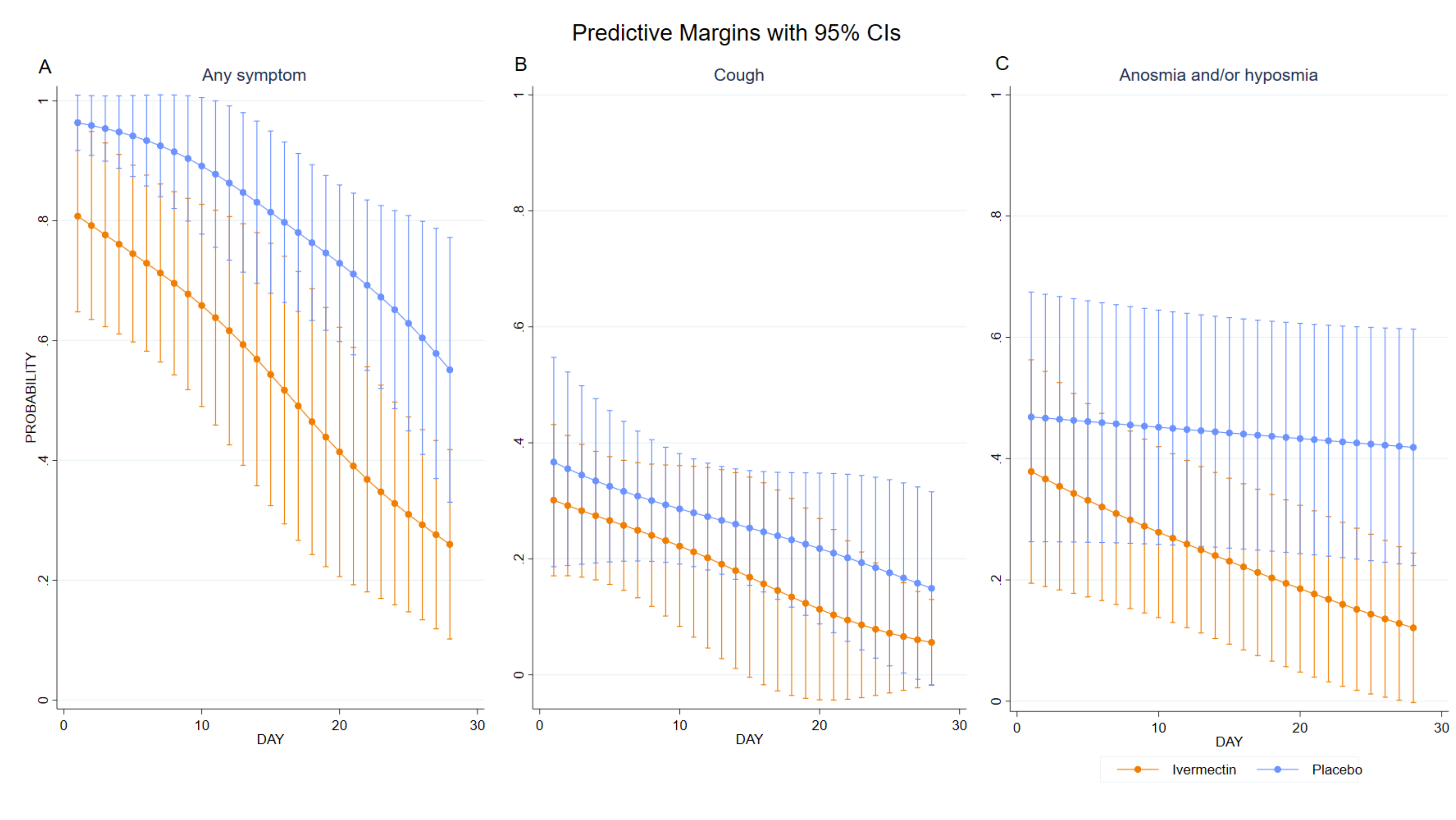

For each symptom, the graph shows the evolution by study arm of its probability during follow up. It was estimated based on mixed effects logistic regression models with subject as a random intercept. These models were adjusted by day of follow up (as symptoms are expected to disappear over time), duration of symptoms before enrolment (as a proxy of disease onset) and the interaction between study arm and day of follow up.

Figure S6: Anosmia/hyposmia by sex and treatment group

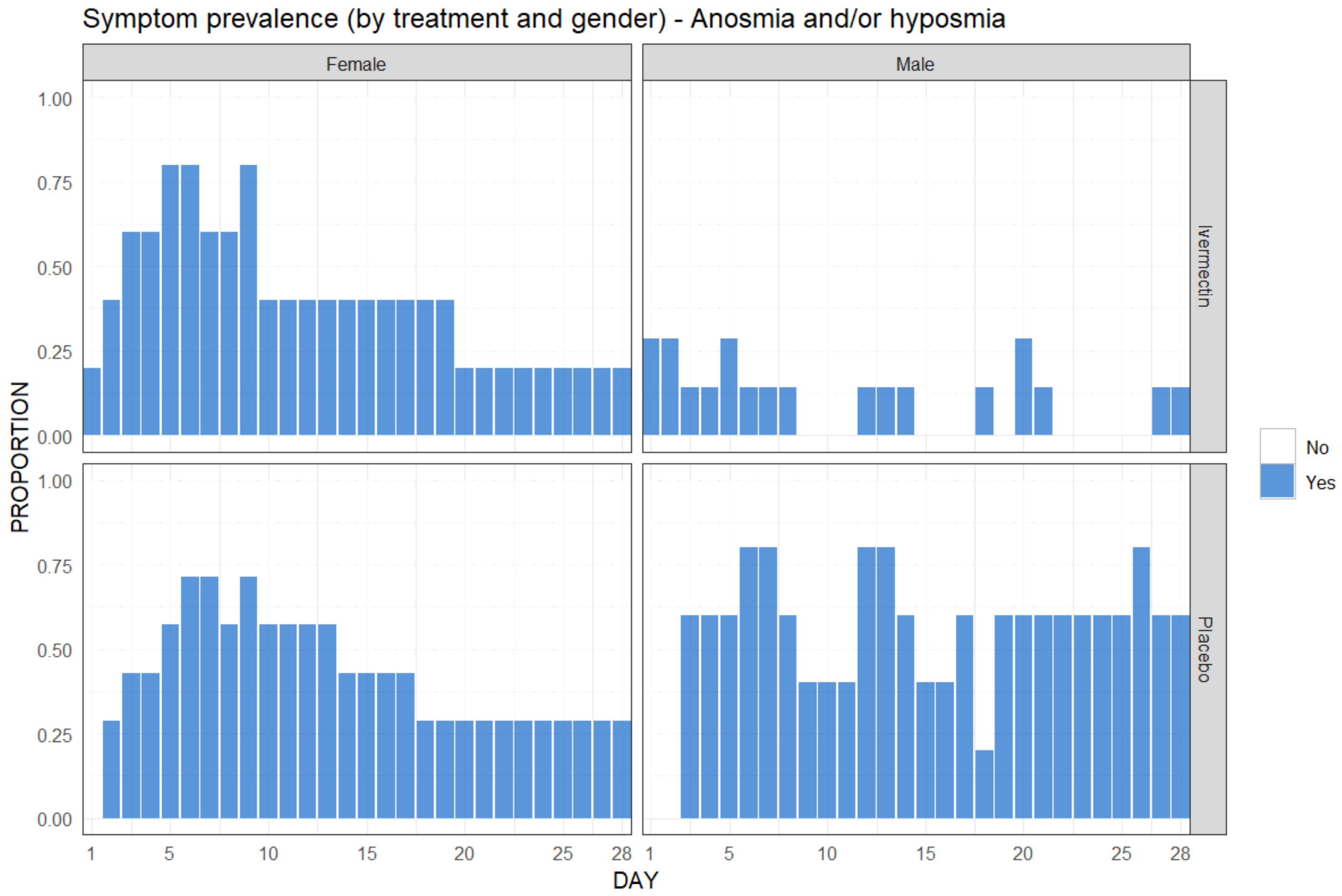

Each individual graph represents the daily proportion of individuals (n/N) who suffered from anosmia/hyposmia in the corresponding study arm for a 28 day follow up.

Figure S7: Ivermectin dose per adipose weight and anosmia/hyposmia

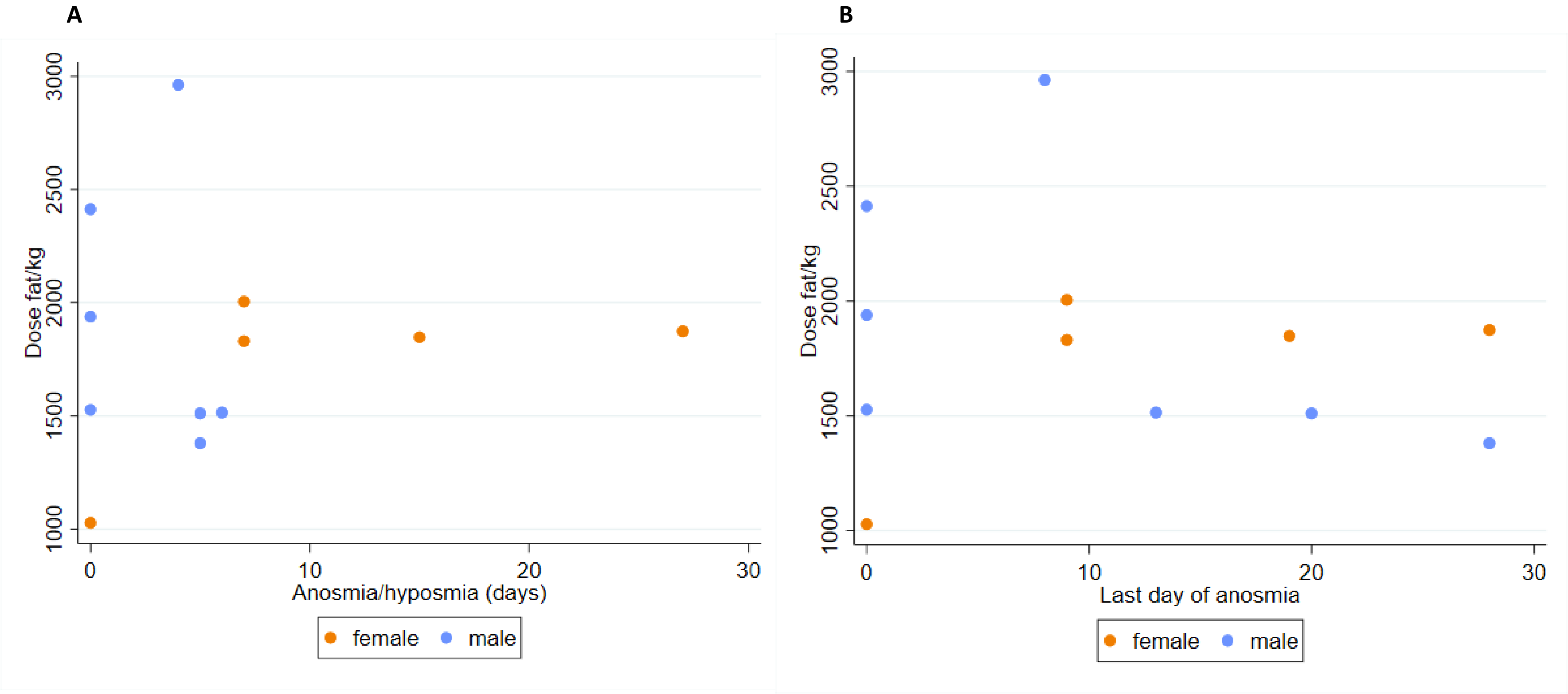

Panel A: duration of anosmia/hyposmia in days, Panel B: last day of reported anosmia
