# Supplementary material for: The effect of early treatment with ivermectin on viral load, symptoms and humoral response in patients with non-severe COVID-19: A pilot, double-blind, placebo-controlled, randomized clinical trial
Source: eClinicalMedicine. 2021 Jan 19;32:100720. doi: 10.1016/j.eclinm.2020.100720 (PMC7816625; doi:10.1016/j.eclinm.2020.100720)
Supplement: Supplementary file 3 [file mmc3.pdf]

CLINICAL TRIAL PROTOCOL

Code: SAINT

Title of trial: Pilot study to evaluate the potential of ivermectin to reduce COVID-19 transmission

Phase: IIa

Date of protocol: 12 of August 2020 - Version 2.0

EudraCT number: 2020-001474-29

Sponsor: Clínica Universidad de Navarra

Written by: Carlos Chaccour MD PhD

Principal Investigator: Dr. Carlos Chaccour

Department: División of Infectious Diseases

Clínica Universidad de Navarra

CONFIDENTIAL MAY NOT BE USED, DIVULGED OR PUBLISHED WITHOUT TRIAL SPONSOR'S CONSENT.

PROTOCOL SIGNATURES SHEET

Protocol: Pilot study to evaluate the potential of ivermectin to reduce COVID-19 transmission

I have read this protocol and accept the obligation to direct this trial in accordance with all the stipulations of the protocol and with the Helsinki Declaration.

Dr. Carlos Chaccour		12 agosto 2020
Principal investigator/<br>trial coordinator	Signature	Date

Dr. Enrique Aubá Guedea		12 agosto 2020
Deputy Chief Medical Officer<br>Clínica Universidad de Navarra	Sponsor's Signature	Date

CONTENTS

1. GENERAL INFORMATION ... 7
1.1. Identification of trial ... 7
1.2. Details of sponsor ... 7
1.3. Person responsible for monitoring ... 7
1.4. Coordinating investigator ... 7
1.5. Details of trial investigators ... 7
1.6. Centers where study takes place ... 8
1.7. Expected duration of trial ... 8
1.8. Number of patients ... 8
2. BACKGROUND AND JUSTIFICATION OF THE STUDY ... 9
2.1. Investigational drug ... 11
2.2. Disease under study ... 16
2.3. Evaluation of benefit/risk ratio for the participants in the clinical trial ... 16
2.4. References from literature ... 16
3. OBJECTIVES OF TRIAL ... 16
3.1 Main objective ... 16
3.2 Secondary objectives ... 17
4. DESIGN OF TRIAL ... 17
4.1. Global design ... 17
5. SELECTION OF STUDY SUBJECTS ... 19
5.1. Inclusion criteria ... 19
5.2. Exclusion criteria ... 19
5.3. Criteria for withdrawal ... 20
6. DESCRIPTION OF TRIAL TREATMENT ... 21
6.1. Method of treatment assignment ... 21
6.2 Pharmaceutical information ... 21
6.3. Drug Identity ... 22
6.4. Potential Risks ... 23
6.5. Administration ... 23
6.6. Packaging and Labeling ... 23
6.7. Handling and Storage ... 24
6.8. Dispensing ... 24
6.9. Accountability ... 25
6.10. Study Intervention Compliance ... 25
6.11. Disposal and Destruction ... 25
6.12. Concomitant Therapy/Devices ... 25
6.13. Dose Escalation ... 26
6.14. Dose Modification and Delay ... 26
6.15. Dose-Limiting Toxicity ... 26
6.16. Overdose ... 27

6.17. Treatment Discontinuation 27
6.18. Maintenance of the randomization codes and the procedures for opening 27
7. EVALUATION OF RESPONSE 27
7.1. Study plan and procedures 27
7.2. Efficacy variables 30
8. SAFETY APPRAISAL 31
8.1. Definitions 31
8.2. Recording and communicating adverse events 32
8.3. SAEs that do not require communication to the person responsible for pharmacovigilance 33
8.4. Procedure for reporting SAEs 33
8.5. Expedited reports of an USAR to the medical authorities 34
8.6. Expedited report of other relevant safety information 34
8.7. Annual safety reports 35
8.8. Reports to investigators 35
8.9. Discontinuation of investigational drugs due to adverse events 35
9. STATISTICS 35
9.1. Description of the statistical methods to be used 35
9.2. Planned number of subjects to be included and study power 36
9.3. The level of significance to be used 36
9.4. Criteria for terminating the trial 36
9.5. Procedure used to account for lost, unused and incorrect data 37
10. ETHICAL ASPECTS 38
10.1. Good Clinical Practice 38
10.2. Ethical considerations 38
10.3. Information for the patient and informed consent 38
10.4. Confidentiality of patients 39
10.5. Compensation for the patient 39
10.6. Insurance policy 39
10.7. Reports to medical authorities 39
10.8. Compliance with protocol 39
10.9. Premature termination of study 40
11. PRACTICAL CONSIDERATIONS 40
11.1. Direct access to source data/documents 40
11.2. Responsibilities of all the participants in the trial 40
11.3. Quality control and assurance 42
11.4. Audit/Inspections 42
12. DATA MANAGEMENT 42
12.1. Case Report Forms (CRF) 42
12.2. Documents to be kept by the investigator 43
12.3. Study archive 43

12.4. Monitoring..43
12.5. Final report..43
13. PUBLICATION OF RESULTS OF TRIAL AND USE OF INFORMATION 43
14. REFERENCES 44
ANNEX 1: SCHEDULE OF VISITS AND EVALUATIONS.. 47
ANNEX 2: DISCRETE DOSING TABLE 48

LIST OF ABBREVIATIONS

AE: adverse event

AEMPS: Agencia Española de Medicamentos y Productos Sanitarios

AR: adverse reaction

CEIm: Comités de Ética de la Investigación con Medicamentos

CIOMS: Council for International Organizations of Medical Sciences

CRF: case report form

Ct: cycle threshold

CUN: Clínica Universidad de Navarra

DENV: dengue virus

DOT: direct observed therapy

EMA: European Medicines Agency

ER: emergency room

FDA: Federal Drug Administration

GCP: good clinical practice

ICF: informed consent form

IP: investigational product

LFU: lost to follow-up

MDA: mass drug administration

NTDs: neglected tropical diseases

PI: principal investigator

PIN: participant identification number

PK: pharmacokinetics

RRP: records reported by patients

SAE: serious adverse event

SUSAR: suspected unexpected serious adverse reaction

USAR: unexpected and serious adverse reaction

1. GENERAL INFORMATION

1.1. Identification of trial

Protocol code: SAINT (SARS-CoV-2 Ivermectin Navarra-ISGlobal Trial)

EUDRACT: 2020-001474-29

Version and date: v2.0 dated: 12 August 2020

Title of trial: Pilot study to evaluate the potential of ivermectin to reduce COVID-19 transmission

1.2. Details of sponsor

CLÍNICA UNIVERSIDAD DE NAVARRA / UNIVERSIDAD DE NAVARRA
Avenida de Pío XII, 36
31008 Pamplona
Email: ucicec@unav.es

1.3. Person responsible for monitoring

Jose Manuel Enciso Gadea

1.4. Coordinating investigator

Dr. Alejandro Fernández Montero
Clínica Universidad de Navarra
Avda. Pío XII 36
31008 Pamplona
Tel.: 948 255 400

1.5. Details of trial investigators

Clínica Universidad de Navarra

PI: Carlos Chaccour

Study coordinator: Alejandro Fernández Montero

José Luis Del Pozo

Francisco Carmona

José Ramón Yuste Ara

Belen Sadaba

José Ramón Azanza

Miriam Fernández

Gabriel Reina

Miriam Giráldez

Felipe Prosper

Susana Inoges

Ascensión López

ISGlobal

Carlota Dobaño

Gemma Moncunill

Regina Rabinovich

Mary Ann Richardson

Paula Ruiz Castillo

Aina Casellas

External collaborators

Felix Hammann, University of Bern.

Joe Brew, Databrew

1.6. Centers where study takes place

Clínica Universidad de Navarra (CUN) and ISGlobal, Barcelona Institute for Global Health

1.7. Expected duration of trial

It is estimated that patients shall be recruited over 1 month, after the initial visit, which shall take place once authorization is obtained from the Spanish Agency of Medicines and Health Care Products (AEMPS).

Each patient shall participate in the clinical for a maximum of 28 days, including the selection phase (1 day), the treatment phase (single dose) and the monitoring phase (28 days).

1.8. Number of patients

24

2. BACKGROUND AND JUSTIFICATION OF THE STUDY

As of April 11, 2020, there were a more than 1.6 million cases of COVID-19 and more than 100,000 deaths worldwide.

Drug-based strategies are widely used in the control of infectious diseases at the population level. Notably, mass drug administration (MDA) for malaria has been used to reduce the impact and transmission of the disease. Mass distribution has also been carried out for other tropical diseases [1].

In the case of COVID-19, three different drug strategies could be considered:

a) **Early treatment**. Assuming that an effective drug is available, this approach requires the identification of patients and targets people with suspected or early confirmed disease. It involves the use of antivirals or immunomodulators that reduce the progression to severe disease and deaths from COVID-19. This strategy would relieve pressure on the health system and decrease indirect deaths due to other diseases requiring intensive care.

(b) **Reducing or blocking transmission.** Assuming that an effective drug is available, this approach involves treating confirmed or suspected cases with a drug that reduces viral excretion in the airway or in the stool. The use of such a drug at Community level could reduce transmissibility and "flatten the curve" of cases, including those that would progress to severe disease, thus allowing a better functioning of the health system.

(c) **Prophylaxis**. This approach consists of providing a drug capable of preventing infection to vulnerable non-immune populations, following the same strategy as currently used to protect travelers from malaria.

A combination drug strategy may also be feasible, for example, a drug that provides early treatment may also reduce the excretion of the virus and decrease transmission.

Coronaviruses, including SARS-CoV-2, are positive, single-stranded RNA viruses that encode a variable number of structural and non-structural proteins. The virus responsible for the former SARS epidemic (SARS-CoV-1) uses transport proteins on the surface of the endoplasmic reticulum to enter the nucleus [2]. In SARS-CoV-2, it has four structural and several nonstructural proteins [3].

The nonstructural helicase plays a central role in the replication of the virus RNA by unwinding the target nucleic acids [4], in the same way as SARS-CoV-1 [5].

Because SARS-CoV-2 is a new pathogen for our species, infections with this virus are associated with a hyperinflammatory state and hypercytokinemia that can cause lung damage and multiple organ failure [6]. This inflammatory profile is well described and includes increased interleukin (IL)-2, IL-7, granulocyte colony-stimulating factor, interferon-inducible protein-10, monocyte chemotractant protein-1, macrophage inflammatory protein-1-9, and tumor necrosis factor-α (TNFa) [7].

Ivermectin is a widely used drug for the treatment and control of certain neglected tropical diseases (NTDs). The drug has an excellent safety profile, with more than 2.5 billion doses distributed in the last 30 years [8]. There are two possible mechanisms by which ivermectin could be useful against COVID-19.

Firstly, ivermectin inhibits the in vitro replication of several similar positive, single-stranded RNA viruses that cause disease in humans and animals, such as dengue (DENV) [9-11], Zika [10, 12] and yellow fever [13, 14], West Nile [10], chikungunya [13], Venezuelan equine encephalitis [15], Semliki Forest virus [13], Sindbis virus [13], porcine reproductive and respiratory syndrome virus [16] and recently SARS-CoV-2 [17].

This inhibition has been observed in an in vitro system with Vero cells at micromolar concentrations of ivermectin (2.5 uM in SARS-CoV-2 and 17-25 uM in DENV). Mastrangelo et al showed that inhibition of DENV NS-3 helicase, an enzyme responsible for unwinding flavivirus dsRNA, occurs at concentrations of 0.5 uM in DENV [14]. No such data yet exists for SARS-CoV-2. While micromolar concentrations are not achievable using approved doses, ivermectin may still be druggable if NS-3 helicase data are any indication.

Strikingly, a clinical trial in Thai Dengue patients showed a reduction in circulating molecular markers following three consecutive daily administrations of ivermectin 400 ug/kg [18]. This cannot be explained by the concentrations obtained from the Vero cell in vitro system. If Vero cells offer any quantitative guidance on ivermectin effect, it would actually give reason to expect a stronger effect in SARS-CoV-2 than in DENV. It is therefore conceivable that a microbiological or clinical effect in vivo may be seen with a single dose of 400 mcg/kg (dose included in the European Union, EU, Drug Factsheet), also given the three-fold levels achieved in pulmonary tissue respective to plasma one week after oral administration [19, 20], the adjuvant role of the immune response and the potential impact of active ivermectin metabolites [20].

However, this trial did not demonstrate a clinical effect, probably because of the long 15-day incubation period of dengue fever, which allows much of the viral replication to occur before the onset of symptoms and treatment with ivermectin.

Second, ivermectin has been shown to have immunomodulatory effects by decreasing TNFa production in vitro [21, 22] and in vivo [21], IL-1 production in vitro [21, 22] and in vivo [21], and IL-10 production in vitro [22]. In addition, ivermectin may also act on T cells by increasing surface receptors in vivo [23].

For this trial we propose to test the use of ivermectin, a safe anti-parasitic drug with broad-spectrum antiviral activity and immunomodulatory properties, against a disease classified as a global public health emergency and for which there is no proven treatment.

We propose a first pilot trial at the Clínica Universidad de Navarra. The trial will use a dose of ivermectin included in its EU drug specification in a population of patients with mild disease and no risk factors for progression to severe disease. The results of this trial could be available as soon as one month after completion of recruitment. In case the trial results are positive and viral

transport is reduced at day 7 post-treatment, it could be used in the short term to reduce transmission at community level.

2.1. Investigational drug

The product under investigation is ivermectin in 3mg tablets manufactured by Merck under the name Stromectol®.

The human ivermectin regimen proposed for SAINT is a single dose of 400 mcg/kg at the enrolment visit. Note that this dose is included in the European Medicines Agency (EMA) approved ivermectin label for the Merck product (Stromectol®) in the Netherlands and France, and for the InfectoPharm product (Scabioral®) in Germany.

The dose will be administered using scales at the site for tailored administration. Given that dosing is limited by the size of the tablet (3mg) the participants will receive a discrete number of tablets according to their weight band as shown in **Table 1.** The individual dose will range from 400 mcg/kg to a maximum of 457 mcg/kg. See annex 2 for a full color-coded table.

Weight in kg	Number of 3 mg tablets	Total dose in mg	Dose range
45	6	18	400
46-52	7	21	404-457
53-60	8	24	400-453
61-67	9	27	403-443
68-75	10	30	400-441
76-82	11	33	402-434
83-90	12	36	400-434
91-97	13	39	402-429
98-100	14	42	420-429

Table 1. Discrete doses based on table size and weight.

The ivermectin dose chosen for the SAINT trial is included in the EU approved label of Stromectol [24] and Scabioral [25]. The only difference in the ivermectin administration for this trial is the evaluation of the drug's activity against SARS-CoV-2, an unknown indication, rather that the direct treatment of other infections for which ivermectin is active.

The elimination of ivermectin is biliary and via metabolic processes, presumably by Cytochrome P_{450} 3A4 (CYP3A4). In healthy volunteers, the reported half-life after oral administration ranged between 12 and 36 hours. This may increase for up to 54 hours in onchocerciasis patients [26]. In addition, it is conceivable that elimination is prolonged when the relevant metabolic pathways are inhibited, such as with co-administration of CYP3A4 inhibitors such as ritonavir (this is incorporated into exclusion criteria).

The ivermectin label has been modified extensively over 30 years of use for treatment and for public health campaigns. The FDA-approved ivermectin dosing regimen for onchocerciasis MDA is 150-200 mcg/kg (every 12 months), although the possibility of quarterly use in individual patients is also included in the label [27]. For moderate to severe crusted scabies, three doses of 200 mcg/kg within two weeks are recommended in the Australian label [28].

The main PK parameters of the dose and regimen proposed dose for SAINT are compared with actual or modelled parameters for the currently label-approved doses of ivermectin in **Table 2.**

See Appendix 1 for a table summarizing trials where ivermectin 400mcg/kg was used.

	**SAINT**<br>**400 mcg/kg single dose**	**Onchocerciasis**<br>**150-200 mcg/kg single [29]**	**Moderate to severe scabies**<br>**200 mcg/kg, 3 doses within 2 weeks [modelled]**
**Cmax**	**63.8**<br>[44- 88.5]	**38**<br>[35 - 41]	**38.3**<br>[27.8 - 52.1]
**AUC**	**2353**<br>[1313-4169]	**1032**<br>[874-1210]	**3532**<br>[1970 – 6266]
**Tmax**	**5.3**<br>[3.9 – 7]	**5.6**	**29**<br>[27.8 - 30.3]

Table 2. Main PK parameters of the ivermectin dose proposed for SAINT as compared with the same parameters for currently approved doses for other indications. PK model by Hammann. All parameters in median [range]. Cmax: ng/ml, AUC ng·h/ml, Tmax: hours

At least six published trials have administered ivermectin at doses above 400 mcg/kg. Table 3 summarizes the available data from those trials. These encompass 2,662 independent administrations of cumulative 800 mcg/kg within one week and 2,103 independent administration at 800 mcg/kg (single dose). Only Kamgno [30] and Smit et al [31] report side effects associated with the drug administered at this high doses. Side effects consisted of mild, transient (hours) visual disturbances without eye structural changes.

Ref	Highest single dose	Max. freq.	Max. number of doses	Maximum total dose (period)	Population	Sample by dose	AEs
Awadzi et al 1995 [32]	800 mcg/kg	Single	Single	800 mcg/kg (once)	Males with moderate to heavy Onchocerca infection	600 mcg/kg x 1: 24 patients<br>800 mcg/kg x 1: 17 patients	No difference with controls taking the 150 mcg/kg dose
Awadzi et al 1999 [33]	800 mcg/kg	Days 1 and 4	2	1.600 mcg/kg (4 days)	Adult males infected with Onchocerca	400-550 mcg/kg x 2: 25 patients<br>600-750 mcg/kg x 2: 23 patients<br>800-950 mcg/kg x 2: 24 patients<br>1600 mcg/kg x 2: 12 patients	No difference with controls taking the 150 mcg/kg dose
Guzzo et al 2002 [34]	2.000 mcg/kg	Days 1, 4 and 7	3	3.273 mcg/kg (one week)	68 male and female healthy, adult volunteers	347-594 mcg/kg x 3: 15 volunteers<br>713-1091 mcg/kg x 3: 12 volunteers<br>1031-1466 mcg/kg x 1: 12 volunteers<br>1404-2000 mcg/kg x 1: 12 volunteers<br>347-541 mcg/kg x 1 (fed): 11 volunteers	No difference with controls
Kamgno et al 2002 [30]	800 mcg/kg	3-monthly	13	8.950 mcg/kg (3 years)	657 adult males infected with Onchocerca	150 mcg/kg yearly: 166 participants<br>400 then 800 mcg-kg yearly: 161 participants<br>150 mcg/kg 3-monthly: 161 participants<br>400 then 800 mcg-kg 3-monthly: 158 participants	The high dose group reported a statistically higher rate of transitory mild and subjective visual side effects.<br>(No structural explanation).<br>For all other AE comparable rates reported in all groups.
Munoz et al 2018 [35]	705 mcg/kg (single 36 mg dose)	Single	Single	705 mcg/kg (once)	54 male and female healthy adult volunteers	276-352 mcg/kg x 1: 18 volunteers<br>227-272 mcg/kg x 1: 19 volunteers<br>157-225 mcg/kg x 1: 20 volunteers<br>553-705 mcg/kg x 1: 18 volunteers<br>455-545 mcg/kg x 1: 19 volunteers<br>315-450 mcg/kg x 1: 20 volunteers	No significant difference compared to weight-adjusted dose of 200 mcg/kg
Smit et al 2018 [31]	600 mcg/kg	Days 1, 2 and 3	3	1.800 mcg/kg (3 days)	141 male and female adults with uncomplicated malaria	300 mcg/kg x 3: 48 participants<br>600 mcg/kg x 3: 47 participants	No difference in SAE. Ivermectin 600 x 3 had 15% more AEs predominantly reflecting transient minor visual disturbances. One case of anaphylaxis was reported in the trial

Table 3. Ivermectin studies assessing the safety of doses above 400 mcg/kg

Adverse reactions

All data in this section is taken from the FDA-approved Merck Stromectrol® 2009 label [27].

When ivermectin is used **in patients with *strongyloidiasis*** (dose of 200 mcg/kg) the following side effects classified by body system have been described:

- Body as a Whole: Asthenia/fatigue (0.9%), abdominal pain (0.9%)
- Gastrointestinal: anorexia (0.9%), constipation (0.9%), diarrhea (1.8%), nausea (1.8%), vomiting (0.9%)
- Nervous System/Psychiatric: dizziness (2.8%), somnolence (0.9%), vertigo (0.9%), tremor (0.9%)
- Skin: pruritus (2.8%), rash (0.9%), and urticaria (0.9%).

When ivermectin is used **in patients with *onchocerciasis*** (dose 150-200 mcg/kg) the following side effects have been described (note this disease is not endemic in Spain):

- Mazzoti reactions during the first 4 days after treatment including arthralgia/synovitis (9.3%), lymph node enlargement or tenderness (up to 13.9%), pruritus (27.5%), urticarial rash (22.7%) and fever (22.6)
- Worsening of pre-existing ophthalmological conditions: limbitis (5.5%), punctate opacity (1.8%).
- Additionally, the following clinical adverse reactions were reported as possibly, probably, or definitely related to the drug in ≥1% of the patients: facial edema (1.2%), peripheral edema (3.2%), orthostatic hypotension (1.1%), and tachycardia (3.5%). Drug-related headache and myalgia occurred in <1% of patients (0.2% and 0.4%, respectively).

Note these side effects have been associated with parasite lysis rather with ivermectin itself.

In patients receiving cumulative doses above 800 mcg/kg in a week, unspecific, mild (not impeding daily activities), transient (lasting less than 24 hours), visual disturbances (blurred vision, tunnel vision) have been described. When ivermectin is used in patients with concomitant *Loa* with high parasite burden (> 15.000 mf/ml) the following side effects have been described in relationship with rapid parasite lysis:

- Encephalopathy in 0.01-0.11% of the treated population [36]
- The syndrome includes confusion, lethargy and coma

This disease is not endemic in Spain. Note that travel to *Loa loa* endemic areas is an exclusion criterion for SAINT.

Laboratory test findings reported as possibly, probably or definitely associated with ivermectin:

- Elevation in liver enzymes (ALT and/or AST) (2%)
- Decrease in leukocyte count (3%).
- Leukopenia and anemia (<1%).

The following adverse reactions have been reported since FDA marketing authorization:

- Hypotension (mainly orthostatic hypotension)
- Worsening of bronchial asthma
- Toxic epidermal necrolysis
- Stevens-Johnson syndrome
- Seizures
- Hepatitis
- Elevation of liver enzymes
- Elevation of bilirubin

Pregnancy

Information from the Stromectol® label [27].

At high doses, well above (ranging from 10-80 fold human therapeutic doses and regimens), ivermectin is a known teratogen in mammals and is classified by FDA as a category C drug (these categories are no longer in use, as FDA now recommends considering risk/benefit on an individual basis). During onchocerciasis MDA campaigns with lower dose within the EMA label, visually pregnant women are excluded but no formal testing is routinely performed on women of childbearing age. There are several reports of inadvertent treatment during pregnancy, a recent metanalysis by Nicolas et al [37] analyzed data from 893 inadvertently exposed pregnancies (97 in the first trimester) and concluded there is insufficient evidence to understand the safety of ivermectin during pregnancy.

Note that pregnancy is an exclusion criterion for SAINT.

Dose justification

SAINT is proposed as a proof of concept pilot trial. Ivermectin has proven antiviral and immunomodulatory activity. There is uncertainty as whether the immunomodulatory effect could increase symptoms. The study has been designed to maximize safety.

The 400 mcg/kg single dose scheme will result in full excretion in 5-7 days. This manages risks related to immunomodulation. The participants will only include those with non-severe disease and no risk factors for severity.

By treating patients within 72 hours of symptoms we aim at reducing early viral replication and improving the potential efficacy of ivermectin. The primary outcome measure is microbiological which makes assessment objective. Secondary objectives including surrogates of viral load and assessment of the immune response will reduce the risk type II error and ensure appropriate evaluation of a drug with potential beneficial effects.

Placebo

The placebo will not be visibly identical, but will be administered by a researcher not involved in the clinical care or participant follow up, helping secure the blind. Placebo is an acceptable control for this trial as there is currently no approved drug for the treatment of SARS-CoV-2, and

the efficacy of ivermectin is to be determined. Placebo tablets will be supplied by Idifarma, and manufactered under GMP.

Other drugs allowed
Supportive measures such as use of antipyretics and hydration as long as they do not interfere with exclusion criteria. Participants may continue to take any drugs and non-pharmacological therapies that are not on the excluded list for the trial

2.2. Disease under study

COVID-19 is the disease caused by infection with SARS-CoV-2, currently classified as a pandemic that can cause severe hyperinflamatory state associated with pneumonia, respiratory and multi-organ failure, particularly in patients over 60 years of age and with comorbidities. There is currently no specific treatment for COVID-19.

The population for the study will be patients with a positive nasopharyngeal swab PCR test for SARS-CoV-2, with non-severe COVID-19 disease and no risk factors for progression to severity (see exclusion criteria). Vulnerable populations such as pregnant women, minors (i.e.; under 18 years old), and seniors (i.e.; over 60 years old) will be excluded.

The control group will receive placebo. There is no current data on the efficacy of ivermectin against the virus *in vivo*, so the use of placebo in the control group is ethically justified. Participants of the study will have non-complicated COVID-19 disease and will not have risk factors to develop severe disease so they would not be receiving any alternative treatment for the disease.

2.3. Evaluation of benefit/risk ratio for the participants in the clinical trial

Ivermectin has a well-documented safety profile at the proposed dose and the potential immunomodulatory and antiviral activity of ivermectin against SARS-CoV-2, outweighs the risk of drug-related adverse events. It is possible, though, that participants will not obtain a direct benefit from taking ivermectin.

2.4. References from literature

See section 14 for all references

3. OBJECTIVES OF TRIAL

3.1 Main objective

To determine the efficacy of a single dose of ivermectin, administered to low risk, non-severe COVID-19 patients in the first 72 hours after symptoms onset to reduce the proportion of patients with detectable SARS-CoV-2 RNA by PCR from nasopharyngeal swab at day seven post-treatment.

Primary outcome measure

Proportion of patients with a positive SARS-CoV-2 PCR from a nasopharyngeal swab at day 7 post-treatment

3.2 Secondary objectives

1. To assess the efficacy of ivermectin to reduce the SARS-CoV-2 viral load in the nasopharyngeal swab at day seven post treatment
2. To assess the efficacy of ivermectin to improve symptom progression in treated patients
3. To assess the proportion of seroconversions at day 21 post-treatment in treated patients
4. To assess the safety of ivermectin at the proposed dose
5. To determine the magnitude of immune response against SARS-CoV-2
6. To assess the early kinetics of immunity against SARS-CoV-2

Secondary outcome measures

1. Mean viral load as determined by PCR cycle threshold (Ct) at baseline and on days 4, 7, 14 and 21 post-treatment
2. Proportion of patients with fever and cough at days 4, 7, 14 and 21 post-treatment as well as proportion of patients progressing to severe disease or death during the trial
3. Proportion of patients with seroconversion at day 21 post-treatment
4. Proportion of drug-related adverse events
5. Levels of IgG, IgM, IgA measured by Luminex, frequencies of innate and SARS-CoV-2-specific T cells assessed by flow cytometry, levels of inflammatory and activation markers measured by Luminex and transcriptomics.
6. Kinetics of IgG, IgM, IgA levels till day 28 post-treatment

4. DESIGN OF TRIAL

The SAINT trial is a double-blind, randomized controlled trial with two parallel groups that evaluates the efficacy of ivermectin in reducing nasal viral carriage at seven days after treatment in SARS-CoV-2 infected patients who are at low risk of progression to severe disease. The trial is currently planned at a single center in Navarra.

4.1. Global design

Participants will be randomized to receive a single dose of 400 mcg/kg ivermectin or a placebo equivalent. The randomization code will be generated by the trial statistician using blocks that ensure balance between the groups.

The allocation will be made by the investigator using opaque envelopes, after obtaining informed consent (note that this might be done verbally during the screening and paper forms

will only be signed after a negative PCR), and confirmation of fulfillment of all inclusion and none of the exclusion criteria. The investigational product will be administered by a researcher not involved in patient care or participant follow up.

Participants will remain in the trial for a period of 28 days.

Trial visits will be conducted either at the COVID-19 department of the Clínica Universidad de Navarra or in the participant's home.

Subsequent visits will be to assess clinical and laboratory parameters.

A final study visit will be made for participants who withdraw prematurely from the study or are withdrawn by the investigator. A summary of the study visits and procedures is provided in Table 4 below.

	**Recruitment** Day 0	**Visit 2** Day 4 post treatment	**Visit 3** Day 7 post treatment	**Visit 4** Day 14 post treatm.	**Visit 5** Day 21 post treatm.	**Final visit** Day 28 post treatm.
Informed consent[#]	X					
Randomization	X					
Clinical data collection	X	X	X	X	X	X
Physical examination	X	X	X	X	X	X
Laboratory test*	X		X	X		
Urine pregnancy test	X					
Nasopharyngeal swab (PCR for SARS-CoV-2)	X	X	X	X	X	
Serology for SARS-CoV-2	X	X	X	X	X	X
Proinflammatory and activation markers	X	X	X	X	X	X
Cell responses (PBMC)	X		X			X
Chest x-ray	X					
Administration of IP	X					
Daily self-assessment of symptoms	X--X					
Evaluation of adverse events	X--X					
[#] verbal consent initially, in writing when isolation is concluded<br>* C reactive protein, Procalcitonin, Ferritin, IL-6<br>, Renal function: urea, creatinine, electrolytes, FBC,<br>Troponin T, CPK, DD, LDH						

Table 4. Visit procedures

Serious adverse events related to ivermectin will be followed until resolved or 30 days after the participant's final visit, whichever occurs first. All other serious adverse events will be followed through to the participant's final visit or for a specified period at the investigator's discretion.

The study shall end when the final randomized patient has completed the study, all the planned visits have been carried out and any inconsistencies have been resolved.

A patient (or their family or legal representative) may interrupt their participation in the study at any time and for whatever reason. The principal investigator may also withdraw a patient from the study if he/she considers that it is in the best interest of the patient.

5. SELECTION OF STUDY SUBJECTS

5.1. Inclusion criteria

1. Patients diagnosed with COVID-19 in the emergency room of the Clínica Universidad de Navarra with a positive SARS-CoV-2 PCR.
2. Residents of the Pamplona basin ("Cuenca de Pamplona")
3. The patient should be between the ages of 18 and 60 years of age
4. Negative pregnancy test for women of child bearing age*
5. The patient or his/her representative, have given consent to participate in the study.
6. The patient should, in the investigator's opinion, be able to comply with all the requirements of the clinical trial.

5.2. Exclusion criteria

1. Known history of Ivermectin allergy
2. Hypersensitivity to any component of Stromectol®
3. COVID-19 Pneumonia
    - Diagnosed by the attending physician
    - Identified in a chest X-ray
4. Fever or cough present for more than 72 hours
5. Positive IgG against SARS-CoV-2 by rapid test
6. Age under 18 or over 60 years
7. The following co-morbidities (or any other disease that might interfere with the study in the eyes of the investigator):
    - Immunosuppression
    - Chronic Obstructive Pulmonary Disease

- Diabetes
- Hypertension
- Obesity
- Acute or chronic renal failure
- History of coronary disease
- History of cerebrovascular disease
- Current neoplasm

8. Recent travel history to countries that are endemic for *Loa loa* (Angola, Cameroon, Central African Republic, Chad, Democratic Republic of Congo, Ethiopia, Equatorial, Guinea, Gabon, Republic of Congo, Nigeria and Sudan)
9. Current use of CYP 3A4 or P-gp inhibitor drugs such as quinidine, amiodarone, diltiazem, spironolactone, verapamil, clarithromycin, erythromycin, itraconazole, ketoconazole, cyclosporine, tacrolimus, indinavir, ritonavir or cobicistat. Use of critical CYP3A4 substrate drugs such as warfarin.

*Women of child bearing age may participate if they use a safe contraceptive method for the entire period of the study and at least one month afterwards. A woman is considered to not have childbearing capacity if she is post-menopausal (minimum of 2 years without menstruation) or has undergone surgical sterilization (at least one month before the study).

5.3. Criteria for withdrawal

Patients will be informed that they may be withdrawn from the study at any time without providing any explanation. Withdrawal may also take place for any of the following reasons:

- Adverse reaction that puts the patient's life at risk, according the principal investigator's judgment.
- Decision made by the patient or the legally authorized person.
- Documented progression of the disease requiring hospitalization in a different center.
- The patient cannot complete the trial visits due to unforeseen circumstances.
- Development of other disorders that, in the opinion of the investigator and in the patient's best interest, make it advisable for him/her to leave the study.
- Administration of treatment with a non-permitted drug.
- The investigator believes that the patient may benefit from a different treatment.
- Loss of monitoring

The investigator shall strive to ensure that the patient agrees to the final trial visit. The data of the withdrawn patients shall be collected up to this final visit. If the withdrawal is the outcome of a Serious adverse event related to ivermectin or SARS-CoV-2, the data shall be collected until the patient recovers or stabilizes.

The subjects shall be replaced if the withdrawal is for a reason different from lack of efficacy of the treatment or appearance of adverse events. All efforts will be made to maintain the total number of assessable patients as per the protocol.
The withdrawal shall be documented in the patient's clinical history, giving the reasons for the decision. A final trial visit should be conducted by the clinical team if possible.

6. DESCRIPTION OF TRIAL TREATMENT

6.1. Method of treatment assignment

This is a single center, randomized, double-blind clinical trial with two parallel groups that evaluates the efficacy of ivermectin in reducing nasal viral carriage at seven days after treatment in SARS-CoV-2 infected patients who are at low risk of progression to severe disease.

Eligible patients shall be enrolled in the study under the supervision of the investigator or designated sub-investigators.

Participants will be randomized to receive a single 400 mcg/kg oral dose of ivermectin or a placebo equivalent. The person administering the drug will observe the participant take it under directly observed treatment (DOT). The placebo will not match ivermectin in appearance. The clinical team will remain blinded to the treatment received by all participants.

6.2 Pharmaceutical information

The investigator shall take responsibility for and take all steps to maintain appropriate records and ensure appropriate supply, handling, storage, distribution, and usage of these materials in accordance with the protocol and any applicable laws and regulations. Please refer to Table 5 for ivermectin pharmaceutical information.

	Ivermectin (STROMECTOL®)
Dose formulation	Tablets<br>White, round, flat tablet with a beveled edge, engraved with MSD on one side and 32 on the other side.
Unit dose strength(s)	400mcg /kg
Route of administration	Oral. Ivermectin will administered as a single oral dose.
Storage	Below 30°C
Packaging and labeling	As required per country requirement according to Annex 13 of GMP.
Dosing instructions[(a)]:	The IP will be administered in accordance with the labeling instructions of a single dose of 400mcg /kg following the discrete dosing described in table 1.

List of excipients	Microcrystalline cellulose<br>Pregelatinized starch<br>Magnesium stearate<br>Butylated hydroxyanisole<br>Citric acid powder (anhydrous).
Incompatibilities	Very rarely, post-marketing reports of increased INR (International Normalised Ratio) have been reported when ivermectin was co-administered with warfarin. Please refer to exclusion criteria and table 6.
Label example	**IVERMECTINA/PLACEBO 3 mg**<br>**___COMPRIMIDOS**<br>**C. Estudio: SAINT**<br>**Caducidad:__________ Lote:_______**<br>SUJETO xxxxxxxx Vía oral<br>Promotor: CUN Conservar 15 a 25ºC<br>**Investigador:** Dr. Chaccour<br>**USO EXCLUSIVO ENSAYO CLÍNICO**
Manufacturer/ Source of Procurement:	Manufacturer : Merck Sharp & Dohme BV Waarderweg 39 2031 BN Haarlem Netherlands<br>Source of Procurement: MSD France<br>The tablets are packaged in aluminum foil blister packs in cardboard cartons. Pack size: 4 tablets.

Table 5 Ivermectin (STROMECTOL®) pharmaceutical information

(a) The Pharmacist or Delegate will prepare the treatment dose required for each patient, according to body weight (400 µg/kg). The Pharmacist or qualified designee completes on site's electronic system. Individual treatment dose is prepared in the dedicated facilities of the pharmacy department by the Pharmacist or designee at a workstation free from unrelated items and in aseptic conditions, as per local rules. Safety measures for preparation and handling include protective clothing, gloves, and the use of safety cabinets.

6.3. Drug Identity

The following information has been taken from the Stromectrol® package insert.

Ivermectin is a semisynthetic, antihelmintic agent derived from the soil bacterium *Streptomyces avermitilis*. Ivermectin is the mixture of 22,23-dihydro-avermectin B1a (at least 90%) and 22,23-dihydro-avermectin B1b (less than 10%).

Ivermectin is a member of the avermectin class of broad-spectrum antiparasitic agents which have a unique mode of action. Compounds of the class bind selectively and with high affinity to glutamate-gated chloride ion channels which occur in invertebrate nerve and muscle cells. This

leads to an increase in the permeability of the cell membrane to chloride ions with hyperpolarization of the nerve or muscle cell, resulting in paralysis and death of the parasite. Compounds of this class may also interact with other ligand-gated chloride channels, such as those gated by the neurotransmitter gamma-aminobutyric acid (GABA).

The selective activity of compounds of this class is attributable to the facts that some mammals do not have glutamate-gated chloride channels and that the avermectins have a low affinity for mammalian GABA-gated chloride channels. In addition, ivermectin does not readily cross the intact blood-brain barrier in humans.

6.4. Potential Risks

Please refer to section 2.1

6.5. Administration

Treatment will be administered in the designated area for COVID-19 patients. 6.6. Packaging and Labeling

Experimental drugs will be supplied by the Department of Pharmacy of the Clínica Universidad de Navarra packaged and labeled according to the standard requirements for clinical trials. Investigational products will be packaged according to the participants' weight (table1). They will be manually introduced in opaque flasks by the study pharmacist. The study medication will be labelled in accordance with annex 13 of the European Good Manufacturing Practices.
Each flask will be clearly marked with the following information:

- Investigational site study code / Sponsor study code:
- Product code:
- Route of administration: For oral administration
- Treatment No: XXXXXXX
- Patient number:
- Theoretical day and time of administration:
- Preparation Date and Time:
- Dose: XXX unit (g, mg, ml…)
- Number of units: XXX tablets
- For clinical trial use only
- Storage conditions: below 30 °C

6.7. Handling and Storage

Ivermectin and placebo will be received at the Pharmacy department following local regulation, with receipt acknowledged by signing the receipt confirmation list that will be filed at site pharmacy file. Lot, batch and manufacturer information of the investigational product will be recorded in the pharmacy records.

All study treatment supplies must be stored in accordance with the manufacturer's instructions and package labeling. Until dispensed to the participants, the study treatment will be stored in a securely locked area, accessible to authorized personnel only. All study interventions must be stored in a secure, environmentally controlled, and monitored (manual or automated) area in accordance with the labeled storage conditions with access limited to the investigator and authorized site staff.

Upon receipt, ivermectin as well as placebo tablets will be stored under 30°C.

The investigator or designee must confirm appropriate temperature conditions have been maintained during transit for all study intervention received and any discrepancies are reported and resolved before use of the study intervention.
If a temperature excursion occurs:
• The affected products will be placed in quarantine immediately with a clear sign of "quarantined" and clearly separated from the rest of the supplies. In addition, the affected products will be stored under proper conditions as per storage requirements.
• The Site Monitor will be informed about the temperature excursion within the same business day.

6.8. Dispensing

Only participants enrolled in the study may receive study intervention and only authorized site staff may supply or administer study intervention. The investigator agrees that study drug will be dispensed by the investigator or sub-investigator(s) named on the Investigator Agreement or their qualified designees. The investigator, sub-investigators, or qualified designees also agree that the study drug(s) will be dispensed only to study subjects who have provided informed consent and have met all entry criteria and in accordance with the instructions provided in the storage and handling manual.

The investigational product dispensation is not managed by IVRS/IWRS. The treatment allocation for ivermectin/placebo will be recorded on site using an electronic system.

6.9. Accountability

The investigator, institution, or the head of the medical institution (where applicable) is responsible for study intervention accountability, reconciliation, and record maintenance (i.e., receipt, reconciliation, and final disposition records).

The Investigator or designee is responsible for maintaining accurate dispensing records of the study treatment throughout the clinical study. The study treatment accountability log includes information including investigational product, number of assigned vials, of IMP used, batch and expiration date of such tablets, dose, patient's identification, quantity of tablets dispensed, person responsible for the accountability register.

Site accountability electronic logs will be available for monitoring visits. All dispensing and accountability records should be stored in accordance to the Sponsor institution regulations.

An accurate accountability record of products received and dispensed, or destroyed by the pharmacy at the investigator site will be maintained on pharmacy file and electronic system.

6.10. Study Intervention Compliance

Participants will receive study intervention directly from a designated nurse. The date and time of each dose administered in the clinic will be recorded in the source documents and reported in the case report form (CRF).

Ivermectin or placebo tablets will be taken orally at the site.

6.11. Disposal and Destruction

Any unused medicinal product or waste material should be disposed of in accordance with local requirements. Approvals for drug destruction at site will be filed in the pharmacy folder in addition to the Certificate of Destruction according to the destruction policy that is in place

6.12. Concomitant Therapy/Devices

Any important concomitant medication that the participant is receiving at the time of enrollment or receives during the study must be recorded along with:

- Reason for use
- Dates of administration including start and end dates
- Dosage information including dose and frequency

The Medical Monitor should be contacted if there are any questions regarding concomitant therapy.

Drugs or treatments that are permitted (including rescue medication) and not permitted before and/or during the trial

Participants may continue to take any drugs and non-pharmacological therapies that are not on the excluded drug list for the trial.

Treatment with inhibitors of CYP3A or the P-gp or other drugs that can interfere with the study drug is an exclusion criterion. See table 6 for details.

Class	Drug	Rationale
Antiarrhytmic/ Antihypertensive	Quinidine	May increase ivermectin exposure by inhibiting its metabolism and excretion or competing with CYPs or the P-gp.
	Amiodarone	
	Diltiazem	
	Spironolactone	
	Verapamil	
Antibiotic- Macrolides	Clarithromycin	
	Erythromycin	
Antifungal agents	Itraconazole	
	Ketoconazole	
Inmunosupressants	Cyclosporine	
	Tacrolimus	
Anti HIV therapy	Indinavir	
	Ritonavir	
	Cobicistat	
Anticoagulant	Warfarin	
Steroids	Dexamethasone	

Table 6 Concurrent treatment used as exclusion criteria

6.13. Dose Escalation

Not applicable. One single dose will be administered.

6.14. Dose Modification and Delay

Not applicable. One single dose will be administered.

6.15. Dose-Limiting Toxicity

Not applicable. One single dose will be administered.

6.16. Overdose

In the event of an overdose, the patient should be closely monitored, treated symptomatically, and supportive measures instituted as required.

6.17. Treatment Discontinuation

Not applicable. One single dose will be administered.

6.18. Maintenance of the randomization codes and the procedures for opening

On occasion, and where an SAE is deemed to be unexpected, related and life threatening at the time, the investigator may ask the Sponsor for unblinding of that participant to identify the treatment received in relation to the SUSAR experienced by the participant. Unblinded participants will be withdrawn from the study and followed through to study end.

Regulatory Authorities and institutional ethics committees will receive unblinded Council for International Organizations of Medical Sciences (CIOMS 1) reports from the trial.

Before the trial begins, the sponsor will assign named independent individuals to code break activity to avoid introducing any bias for the designated safety physician, clinical team, sponsor and statistician teams.

Procedures for code breaking at the end of study

a. Unblinding of the study can only occur after all participants have completed the final visit and the database has been cleaned and locked.

b. The coordinating investigator documents the date of the last trial visit and the data lock.

c. The coordinating investigator gives approval for the statistician to break the code and documents the breaking of the code in the study folder

d. The coordinating investigator informs the PI of the treatment status of each group only after all the data analysis is complete.

7. EVALUATION OF RESPONSE

7.1. Study plan and procedures

7.1.1. Obtaining consent

During this SARS-CoV-2 pandemic period, documented verbal consent in lieu of patient signature should be obtained. Once the entire study is explained, a verbally informed consent shall be obtained from the patient before their participation in the study may be made effective. Investigator must fully inform the patient of what they are consenting to, and must document consent was verbally received.

Subsequent efforts will be made to ratify the written consent or to obtain a written informed consent from their relatives or representative.

The method used to obtain and document the informed consent and the contents of same should comply with the EMA recommendations: “Guidance on the Management of Clinical Trials during the COVID-19 (Coronavirus) pandemic Version 2 (27/03/2020)” [38].

Part of the process of the informed consent consists of clearly explaining the purposes, methods, objectives and risks of the study to the patient, their family or legal representative. A copy of the informed consent, also signed by the investigator, will be kept with the clinical documentation. Another copy will be given to the patient.

The attending physician will record the patient's consent to participate in the study in his/her clinical documentation.

7.1.2. Screening, enrollment and first visit

Once the informed consent process has been completed, the information required for the trial may be obtained, and the evaluations to confirm trial inclusion and exclusion criteria conducted.

At this visit the following procedures will take place:

- Appropriately trained and delegated trial staff will explain the study objectives, methods and procedures to patients who will be invited to participate in the study. The trial staff will explain the inclusion and exclusion criteria and offer to answer questions as part of the informed consent process.
- During the consent procedure, women of child-bearing age will be informed of the hazards to an unborn child and will be asked about the current contraceptive method they are using. They will be informed that if their pregnancy test is positive, they cannot participate in the trial.
- A site visit template containing visit related questions will be completed by the trial staff as it is answered by each participant
- Trial staff will document the participant’s medical history and all concomitant medication to confirm eligibility to participate in the trial
- A urine-based pregnancy test will be administered to all women of child bearing age
- Participants will be given a chest x-ray to rule out pneumonia.
- Eligible participants will be randomized to the trial and allocated a Participant Identification Number (PIN).
- The trial staff will perform a physical examination including anthropometric assessment.

- Approximately 20ml of blood will be drawn to record baseline laboratory values for C reactive protein, Procalcitonin, Renal function: urea, creatinine, electrolytes, FBC, Troponin T, CPK, DD, LDH.
- Approximately 4mL of blood will be drawn for serological assays and assess proinflammatory and activation markers.
- Approximately 10 mL of blood will be drawn for cellular assays.
- A nasopharyngeal swab will be taken to confirm the presence of SARS-CoV-2 RNA.
- A rapid serology test for SARS-CoV-2will be conducted to assess the current immune status.
- The investigational product will be administered following a weight-band table. Immediate tolerance will be monitored.
- A link to the trial website with a self-assessment questionnaire will be given to each participant with brief instructions for daily completion and an explanation of the review process with the clinical team at all subsequent trial visits.

7.1.3. Subsequent trial visits

Subsequent visits will be conducted at the COVID-19 department of the Clínica Universidad de Navarra or at the participant´s home until obtaining a negative PCR in the nasopharyngeal swab after which the patient may attend te conventional ambulatory clinic.

In SAINT, patients will self-monitor symptoms daily. A study physician will visit the participants five times during the trial to assess symptoms and potential side effects as well as to perform a physical examination.

At the trial visits on post-treatment days 4, 7, 14 and 21:

- The clinical trial team will confirm that the participant is still willing to participate in the trial.
- A member of the clinical trial team will ask the participant about symptoms progression and document the information in the visit template.
- A member of the clinical trial team will review the information that the participant has recorded in the daily online self-assessment with the participant and ask whether the participant has taken any medication or sought healthcare since the last trial visit.
- The trial staff will perform a physical examination.
- A nasopharyngeal swab will be taken to confirm the presence of SARS-CoV-2 RNA.
- A serology test for SARS-CoV-2 will be conducted to assess the current immune status.

- 4mL of blood will be drawn for serological assays and assess proinflammatory and activation markers.

Additional procedure at the trial visit on post-treatment days 7 and 14:

- A blood sample will be drawn to assess any changes in laboratory parameters since the recruitment visit

Additional procedure at the trial visit on post-treatment days 7 and 28:

- 10 mL of blood will be drawn for cellular assays.

Final trial visit on post-treatment day 28:

- A member of the clinical trial team will ask the participant about symptoms progression and document the information in the visit template
- A member of the clinical trial team will review the information that the participant has recorded in the daily online self-assessment with the participant and ask whether the participant has taken any medication or sought healthcare since the last trial visit
- The trial staff will perform a physical examination
- A serology test for SARS-CoV-2 will be conducted to assess the current immune status
- 4mL of blood will be drawn for serological assays and assess proinflammatory and activation markers.
- 10 mL of blood will be drawn for cellular assays.

7.2. Efficacy variables

Primary

Proportion of patients with a positive SARS-CoV-2 PCR from a nasopharyngeal swab at day 7 post-treatment

Secondary

1. Mean viral load as determined by PCR cycle threshold (Ct) at enrollment and days 4, 7, 14 and 21 post-treatment
2. Proportion of patients with fever and cough at days 4, 7, 14 and 21 post-treatment
3. Proportion of patients with seroconversion at day 21 post treatment
4. Levels of IgG, IgM, IgA measured by Luminex, frequencies of innate and T cell responses assessed by flow cytometry, levels of inflammatory and activation markers measured by Luminex and transcriptomics.

5. Kinetics of IgG, IgM, IgA levels till day 28 post-treatment.

8. SAFETY APPRAISAL

All the adverse events reported by patients or observed by the investigating team shall be evaluated, including any clinically relevant analytical alterations.

Any AE spontaneously referred by a patient or that appears as a result of the investigator's anamnesis shall be noted in the patient's clinical history, specifying the time it appeared, evolution, duration, intensity, therapy required and relation to the investigational drug. Common Terminology Criteria for Adverse Events (CTCAE 5.0) will be used.

8.1. Definitions

Adverse event: defined as any incident that is hazardous to the health of a patient or subject of a clinical trial treated with a drug, even when there is not necessarily a causal relationship with said therapy.

An AE can therefore be any unfavorable and unintended sign (including an abnormal laboratory finding), symptom or disease temporarily associated with the use of an investigational drug, regardless of whether or not it is related to the investigational drug.

Serious adverse event: defined as any adverse event or adverse reaction that, at any dose:

- Causes the death of the patient.
- Threatens the patient's life
- Requires the patient to be hospitalized or his/her hospitalization to be extended
- Causes permanent or major invalidity or incapacity
- Gives rise to a congenital abnormality or deformation

For the purposes of notification, any suspicions of an adverse event or reaction regarded as potentially important from a medical point of view shall also be considered to be serious, even when they do not comply with the above criteria, including important medical events that require an intervention to prevent any of the above-mentioned consequences from taking place. Likewise, all suspicions of transmission of an infectious agent via a drug shall also be reported as serious events.

Adverse reaction (AR)

Any toxic and unintended reaction to an investigational drug is considered to be an adverse reaction, independently of the dose administered.

Unlike an AE, there is a suspected causal relationship between the investigational drug and the adverse event.

The possible relationship with the therapy of the study shall be established in accordance with the following definitions:

**Relation**	**Definition**
UNRELATED	There is no evidence of any causal relationship.
UNLIKELY	There is little evidence to suggest a causal relationship (e.g. the event did not present in a reasonable time frame after the administration of the drug/procedure under study). There is another explanation for the event (e.g. the patient's clinical conditions, other concomitant therapies).
POSSIBLE	There is evidence that suggests a possible causal relationship (e.g. because the event took place in a reasonable time frame after administration of the investigational drug). However, the influence of other factors may have contributed to the event (e.g. the patient's clinical condition, other concomitant therapies).
PROBABLE	There is evidence that suggests a causal relationship and the influence of other factors is unlikely.
CERTAIN	There is clear evidence to suggest a causal relationship and a possible contribution from other factors can be dismissed.

An adverse reaction should be considered as any adverse event that is possibly, probably or certainly linked to the therapy under study.

The principal investigator of the research center or person appointed by same is responsible for establishing the possible relationship with the therapy under study.

Unexpected and Serious Adverse Reaction (USAR)

Any serious adverse reaction whose nature, intensity or consequences does not match the reference information for the drug (e.g. the Investigator's Brochure in the case of an unauthorized investigational drug or the Summary of Product Characteristics in the case of an authorized drug).

The unexpected nature of an adverse reaction is based on the fact that it was not previously observed and shall not be based on what could be anticipated in view of the pharmacological properties of the drug.

8.2. Recording and communicating adverse events

All the reported adverse events shall be recorded, either spontaneously by the patients or during the interviews held with them in the study visits. The AE from when the patient received the first dose of the investigational drug until the end of his/her participation shall be collected.

The patient should be requested to give all the information about the AEs that took place in the time period between the previous visit and the current one.

All the AE should be documented in the patient's clinical history and in the CRF.

When the investigator considers an adverse event to be a serious one, he/she should immediately notify the sponsor as soon as he/she is aware of it.

8.3. SAEs that do not require communication to the person responsible for pharmacovigilance

The investigator should immediately report all the SAEs, as per the above-mentioned definitions, with the exception of the following serious adverse events:

- SAEs occurring after 5 days of treatment (as the half-life of ivermectin is 17 hours)
- Hospitalization or death due to progression of the disease.

All SAEs shall be recorded in the patient's clinical history and the CRF.

The other SAEs should be reported as per the procedure described in point 8.4.

8.4. Procedure for reporting SAEs

If there is an SAE that should be reported to the person responsible for pharmacovigilance, a member of the investigating team shall complete and sign an SAE, which shall be sent scanned by email, immediately and always within the 24 hours following the moment when the event was known of.

The person responsible for pharmacovigilance shall check the form received and, if necessary, shall ask the investigator for additional information.

When additional information about the SAE is obtained, or the situation is resolved or is unlikely to change, a monitoring report should be completed and likewise sent scanned by email to the appointed person responsible for pharmacovigilance.

If it is suspected that the SAE may be a SUSAR, the investigator should provide any monitoring information that might be requested.

Any SAE that is discovered and that takes place in the month following the end of the trial should be reported (with no time limit), if the investigator considers that the SAE is related to the investigational therapy (i.e. if it is a serious adverse reaction) or is medically important.

Pregnancy

All women of child bearing potential will have a urine pregnancy test to confirm inclusion/exclusion criteria. As the IP is only administered at the first visit there will be no further pregnancy tests during the trial.

During onchocerciasis MDA campaigns with lower doses within the EMA label, visually pregnant women are excluded but no formal testing is routinely performed on women of childbearing age. There are several reports of inadvertent treatment during pregnancy, a recent metanalysis by Nicolas et al [37]. analyzed data from 893 inadvertently exposed pregnancies (97 in the first trimester) and concluded there is insufficient evidence to understand the safety of ivermectin during pregnancy

8.5. Expedited reports of an USAR to the medical authorities

The person responsible for pharmacovigilance is responsible for reporting to the AEMPS (Clinical testing department of the General Sub-directorate for Medicines for Human Use) and to the Spanish autonomous communities, about all the USARs contained in the study, following the procedures indicated by legislation currently in force.

8.5.1. Report deadlines

The maximum period for reporting an individual case of a suspected USAR shall be 15 calendar days dating from when the sponsor was aware of same. When the suspected USAR has caused a patient's death, or put his/her life in danger, the sponsor shall send the information within 7 calendar days dating from when the sponsor was made aware of same. He shall complete the report whenever possible in the following 8 days.

8.6. Expedited report of other relevant safety information

Expedited reports shall also be issued of any information that might change the risk/benefit balance of the investigational drug, or determine changes in its administration schedule or in carrying out the test, such as:

- A qualitative change or increase in the percentage of expected serious ADRs considered to be clinically important.
- New developments related to the trial and that may affect the patients' safety, such as:
    - SAEs that may be associated with the trial procedures and that might modify how it is carried out.
    - A significant risk to subjects, such a lack of efficacy of a drug used to treat a life-threatening disease.
    - New important findings about safety that come from new animal trials (such as carcinogenicity).
    - Any premature termination or temporary halt of a clinical trial with the same investigational drug for safety reasons, carried out in another country by the same sponsor

This relevant information shall be reported as soon as possible and no later than the 15 days from when the sponsor was made aware of same.

The additional information shall also be reported as soon as possible.

8.7. Annual safety reports

The annual reports that include the SUSARs and SAEs in the study shall be sent to the AEMPS (Clinical testing department of the General Subdirectorate for Medicines for Human Use), the autonomous communities and the CEIm, within the deadlines established by legislation currently in force. This will be included as an appendix of the study report as the study is expected to be completed within two months.

8.8. Reports to investigators

The investigators shall be given any safety information that might affect the safety of the trial subjects as soon as possible.

Information shall also be issued throughout the study of any aspect of safety that might have an impact on the clinical trial, including modifications to the protocol that are linked to safety.

8.9. Discontinuation of investigational drugs due to adverse events

The investigational will be administered as a single dose.

9. STATISTICS

9.1. Description of the statistical methods to be used

Baseline data from patients enrolled in both arms will be described. This descriptive analysis will use frequency, median and interquartile range for qualitative and quantitative variables respectively.

All analyses will be done using the STATA and R program.

EVALUATION OF EFFICACY

The primary outcome measure will be assessed using Fisher´s exact test. This will allow for comparing the proportion of participants with positive PCR at day seven post treatment.

The analysis will be done per protocol, including all randomized participants for whom there is a PCR result at day seven. Randomized patients without day seven results Will be described to verify there is no common pattern.

SAFETY EVALUATION

The analysis of safety will be done using a modified intention to treat approach considering all randomized and treated participants. AEs experienced by treated participants in each study arm will be described separately.

All the adverse events that occur during the study will be included in the data lists and organized according to each patient. Events that are considered to be related to the treatment (possibly, probably or definitely related to it) will also be included in a table. A table will with the list the

adverse events according to maximum intensity will be provided. The deaths and serious adverse events will also be classified in a separate table.

9.2. Planned number of subjects to be included and study power

The study will enroll 24 participants, 12 per arm. The sample size has been calculated for comparing two proportions. This sample provides 80% power at 5% significance level using fisher´s unilateral test to determine a difference of 45% (100% vs 55%) in the proportion of participants with positive PCR at day 7 post-treatment. This includes up to one participant lost to follow up (LFU) per arm. We estimated this low LFU level given that the patients will be isolated at home and will have a low risk of progression to severe disease. The 100% positivity rate at day seven is based on the clinical experience at the Clínica Universidad de Navarra. Figure 1 provides the relationship power-outcome measure for the full sample and one scenario considering LFU of up to two patients per arm.

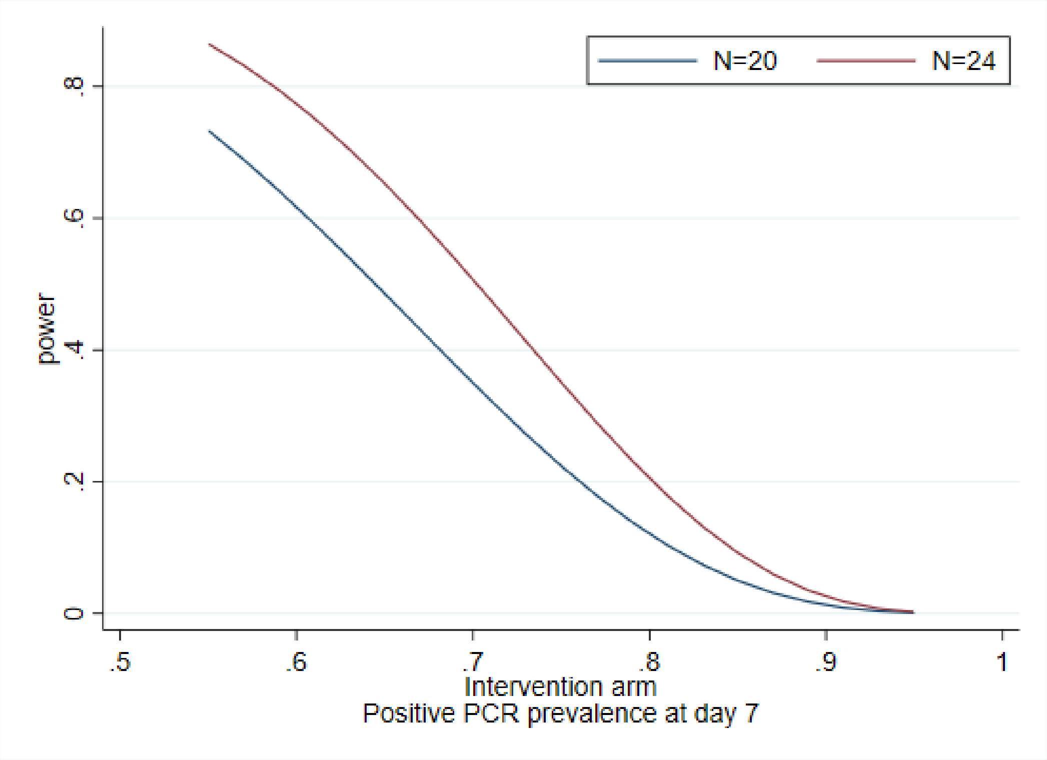

Figure 1. Trial power scenarios for different effect size and loss to follow up levels.

9.3. The level of significance to be used

The significance level has been established at 5%

9.4. Criteria for terminating the trial

The study will normally end when the planned number of patients has been recruited, the last patient has completed the study and all the inconsistencies and adverse events have been

resolved. The sponsor is entitled to terminate the study and withdraw all the study materials at any time.

The reason for which it may be necessary to terminate the study:

- The incidence and/or severity of adverse events in this or other studies indicates a possible risk to health from treatment with the experimental or reference drug.

In all these cases a final examination should be carried out on each patient who remains in the study at the time it is closed or prematurely terminated. The investigator shall complete the Case Report Forms in as much detail as possible. The completed Case Report Forms and the study materials shall be collected by the sponsor.

9.5. Procedure used to account for lost, unused and incorrect data

All the available data on safety and efficacy shall be included in the data lists and tables. No values shall be allocated for unavailable data.

Any confusing or incorrect data shall be examined in accordance with the standardized data control procedures.

In the analysis of the records reported by patients (RRP) the omitted data shall be allocated by using the last observation carried over. The omitted standard shall be examined before any allocation. If the omitted standard is clearly informative, the repercussion of the non-random data shall be evaluated using sensitivity analysis.

For the RRP analysis, if a section of subscale from several is missing, the average of the remaining sections shall be used as a scoring scale, as long as at least half of the sections of the scale are available.

9.6. Procedure for reporting all the deviations from the original statistical plan

All the deviations from the original statistical analysis plan will be included in the final report of the clinical trial.

9.7. Selection of the subjects to be included in each analysis

The efficacy analysis will be done per protocol, including all randomized participants for whom there is a PCR result at day seven.

The analysis of safety will be done using a modified intention to treat approach considering all randomized and treated participants.

10. ETHICAL ASPECTS

10.1. Good Clinical Practice

The study shall be carried out in accordance with the International Conference on Harmonization (ICH) regarding good clinical practice and the corresponding regulatory requirements. The investigator shall be completely familiar with the correct use of the investigational drug as described in the protocol. The essential clinical documents shall be kept to demonstrate the validity of the study and the integrity of the collected data. The main files should be established when the study starts, shall be kept over the course of the study and stored in accordance with the relevant legislation.

10.2. Ethical considerations

The study will be carried out in accordance with the ethical principles of the latest revision of the Helsinki Declaration and with legislation currently in force. The CEIm shall examine all the documentation relating to the study in order to protect the patients' rights, safety and wellbeing. The study shall only be carried out at the centers for which approval has been obtained from the CEIm. The protocol shall be submitted to the CEIm, along with the informed consent, advertising, the written information given to patients, updates linked to safety, annual progress reports and any changes made to the above documents.

This study may only start after a favorable decision in writing is received from the CEIm and approval is given by the AEMPS.

10.3. Information for the patient and informed consent

During this SARS-CoV-2 pandemic period, documented verbal consent in lieu of patient signature should be obtained. Once the entire study is explained, a verbal informed consent shall be obtained from the patient before their participation in the study may be made effective. Investigator must fully inform the patient of what they are consenting to, and must document consent was verbally received.

Subsequent efforts will be made to ratify the written consent or to obtain a written informed consent from their relatives or representative.

The method used to obtain and document the informed consent and the contents of same should comply with the EMA recommendations: "Guidance on the Management of Clinical Trials during the COVID-19 (Coronavirus) pandemic Version 2 (27/03/2020)"[38].

The investigator (or person delegated by same) shall sign and date the Consent Form. The investigator shall file the original consent form in the Investigator's File in the center.

The patient shall receive the Informed Consent Form and shall be informed that participation in the study is voluntary and may be withdrawn at any time without prejudice to the prior medical care. Neither the Patient Information Sheet or the Informed Consent can be modified without agreement from the CEIm and the sponsor.

A copy of the Informed Consent Form will be kept by the patient.

The Consent Form includes information about the need to inspect the Clinical Histories and to enable basic data to be obtained in the event that the patient decides to suspend participation for reasons different from the withdrawal of consent.

10.4. Confidentiality of patients

With a view to respecting patients' privacy, the patients will be identified with an assigned patient number in all the case report forms, investigational drug accountability records, reports and communiqués of the study. The investigator shall provide inspectors and any possible auditors or collaborators appointed by the sponsor and the regulatory authorities with access to original records of the patients so that they can verify the data in the case report forms and audit the data collection process. Confidentiality shall be maintained and the patient's identity shall not be made public, to the extent permitted by relevant legislation and regulations.

Confidentiality shall be maintained and the patient's identity shall not be made public, to the extent permitted by relevant legislation and regulations (Law 03/2018).

10.5. Compensation for the patient

The investigational drug and the tests deriving from the study will be financed by the sponsor. The sponsor will not bear the costs of the habitual care of the patient, in other words, any procedures that would be practiced or treatments that would be received independently of their participation in the study.

10.6. Insurance policy

The sponsor has taken out an insurance policy that covers, in its terms and conditions, the legal liability for damages caused to participants and deriving from the investigation, carried out strictly in accordance with the scientific protocol and legislation currently in force (RD 1090/2015).

10.7. Reports to medical authorities

This trial requires an application to be sent to the Director of the Spanish Agency of Medicines and Health Care Products (AEMPS) for its approval. The study must be approved by the AEMPS before it can start.

10.8. Compliance with protocol

The investigator will carry out the study in accordance with the protocol provided by the sponsor and once approval or a favorable decision is obtained from the CEIm and the relevant regulatory authorities. The protocol should not be changed without the consent of the investigator and the sponsor. Any relevant changes to the protocol require approval or a favorable decision in writing from the CEIm prior to its implementation unless the modification is necessary to prevent immediate risks to patients. The sponsor shall present all the changes made in the protocol to the regulatory authorities in accordance with legislation currently in force.

When an immediate deviation of the protocol is required to prevent immediate risks to patients, the investigator shall contact the sponsor, if the circumstances so permit, to consider the measures to be adopted. Any deviation from the protocol should be documented in detail in the CRF and the original documentation.

10.9. Premature termination of study

This study may be prematurely interrupted if the sponsor or the regulatory authorities consider that there is sufficient cause to do so. The investigator shall receive written notification in which the transferring party documents the reason for suspending the study.

The circumstances that can justify the suspension of the study include but are not limited to:

- Establishment of unexpected risks that are considerable or unacceptable for patients
- Insufficient number of patients registered
- Insufficient compliance with protocol requirements.

The reason for which it may be necessary to terminate the study:

- The incidence and/or severity of adverse events in this or other studies indicates a possible risk to health from treatment with the experimental or reference drug.

In all these cases a final examination should be carried out on each patient who remains in the study at the time it is closed or prematurely terminated. The investigator shall complete the Case Report Forms in as much detail as possible. The completed Case Report Forms and the study materials shall be collected by the sponsor.

11. PRACTICAL CONSIDERATIONS

11.1. Direct access to source data/documents

The sponsor shall guarantee in the protocol or other written agreement that the investigator or the institution shall permit direct access to the source data or documents for monitoring, auditing, revision by the CEIm, and for inspection of the trial by the medical authorities.

11.2. Responsibilities of all the participants in the trial

Investigator

The investigator should agree with this protocol and have an in-depth knowledge of the properties of the products used in the clinical trial.

The investigator should give the information sheet to the patient and collaborate with him/her to help them understand the explanation provided in the document. It is important for him/her to inform patients that their participation in the study is totally voluntary and that it does not affect the doctor/patient relationship, and to assure them that all the persons involved in the study shall respect the confidential nature of any information relating to the patient.

The Principal Investigator or one of his/her collaborators shall be responsible for correctly collecting, recording and reporting the data and shall ensure that any serious or unexpected adverse events shall be reported within 24 hours.

It is the duty of the investigator to regularly inform the CEIm of the progress of the study and he/she shall be jointly responsible with the sponsor in preparing the final report.

Monitor

The monitor is responsible for directly monitoring the trial process in line with the directive of Good Clinical Practices (GCP) and legislation currently in force. He/she will carry out a kick-off visit to the study centers to check the qualifications of each investigator, inspect the facilities of the study center and inform the investigator about the responsibilities and procedures to guarantee adequate and correct documentation of the study. This meeting shall include an inventory of the materials and a detailed revision of the protocol and the CRF.

The investigator is obliged to prepare and maintain adequate and exact records of the patients, designed to log all the observations and other relevant data to the study of each participant in the study. All the information recorded in the CRFs for this study should match the patients' original documentation.

All the study data recorded in the original documents shall be transcribed in the CRFs. The study data shall be entered in a data processing system that is validated and secure and backup copies shall be kept. Any changes to the study data shall be documented.

Over the course of the study the monitor shall check for compliance with the protocol, compare the CRFs and the medical histories of individual patients, evaluate the accountability of the drugs and ensure that the study is being developed in accordance with the relevant legal requirements. The CRFs shall be verified by comparing to the original documentation. The medical histories shall be checked in such a way as to ensure the patients' confidentiality. A copy of the final CRF shall be left in the investigator's study file and the original shall be collected by the study monitor.

The investigator shall permit monitoring and audits of the study by personnel authorized by the sponsor, along with revision by the CEIm and medical authorities, and shall provide them with direct access to the source data/documents.

Sponsor

The sponsor of the clinical trial is the natural person or legal entity that has an interest in completing it, signs the applications for authorization sent to the CEIm and/or the Spanish Agency of Medicines and Health Care Products (AEMPS) and is responsible for same, including its execution, commencement and completion. The sponsor shall also be responsible for ensuring compliance with the relevant legal standards.

The sponsor takes on the obligations of a sponsor contained in legislation currently in force, providing all the resources and collaborators required to fulfill said responsibility with full guarantees.

The sponsor shall provide the investigator with an Investigator's File. This file shall be used for all the relevant documents related to the study. The investigator shall be responsible for updating the Investigator's File, checking that all the required documents are included during and after the study. The file shall be inspected during the monitoring visits and shall be kept by the investigator after the study.

11.3. Quality control and assurance

The study shall be regularly monitored. A representative of the sponsor shall analyze the protocol with the investigators and their staff during the kick-off visit at the study center or in a meeting with the investigators.

The investigator should provide the monitor with access to the appropriate hospital record or the clinical histories to confirm the accuracy of the data entered in the CRF. None of the information contained in said records about the identity of the patients shall leave the study center. The monitor shall check that all the patients have duly given their consent and that the signed informed consent is available after the last study visit. He/she shall also review the inclusion and exclusion criteria to ensure that all the patients are duly included in the study.

The monitor shall ensure that all the AEs, SAEs and deviations from the protocol are appropriately noted and documented. Other verifications shall be carried out on the match between the original data and the CRFs in accordance with the specific monitoring plant of the study.

11.4. Audit/Inspections

The regulatory authorities, the CEIm and the sponsor or an appointed representative may ask for access to all the original documents, case report forms of the patients and other documentation of the study in order to carry out an audit or inspection at the center. The investigator should guarantee direct access to these documents and collaborate at all times in said activities.

12. DATA MANAGEMENT

12.1. Case Report Forms (CRF)

CRFs shall be completed by the investigation team, transcribing the data from the original documents that form the patient's clinical history. The investigator shall sign the CRF to guarantee its authenticity and accuracy. The CRF may be completed by any authorized person, whose signature is recognized. The completed CRF shall be checked during the monitoring visit.

Daily self-assessment of symptoms will be written in XLSform, entered via web the enketo web application, transferred securely via https, and stored in an encrypted PostgreSQL database. Back-ups of the database will be generated daily in the form of a SQL “dump” file, and a paper back-up will be generated weekly. Only the PI and his/her designated deputies will have access to the database. Any changes to the database will be logged in a change ledger, along with information pertaining to why the change was made, who it was made by, and a timestamp. Upon completion of the study, an encrypted CD-ROM storing the digital CRFs will be generated for archiving.

Any outcome outside the range of normality shall be duly commented.

12.2. Documents to be kept by the investigator

The investigator shall keep all the records of the study which includes all the original documents of the clinical history and a copy of all the CRFs and the identification list of the subjects in accordance with the good clinical practices of the International Conference on Harmonization (ICH) and the corresponding regulatory requirements.

12.3. Study archive

The Clínica Universidad de Navarra shall keep a Principal Archive of the Study for the lifetime of the product.

12.4. Monitoring

The sponsor shall assign monitoring staff to the clinical trial. Their functions shall include assisting the investigator and the sponsor in keeping the data completely legible, well organized and easy to recover. They shall also explain and make sure that the investigator understands and interprets all the sponsor's applicable SOPs and the relevant provisions regarding the clinical evaluation of an investigational drug. They shall also ensure that the protocol is correctly understood, and they shall give information about responsibilities and the validity of the data.

The initial notes (raw data) about the key points of their status, such as the AEs and laboratory trials, shall be kept in the Clinical History.

12.5. Final report

The investigation team shall draw up a report suitable for presentation to the relevant authorities.

13. PUBLICATION OF RESULTS OF TRIAL AND USE OF INFORMATION

The results of this clinical trial shall be published in scientific journals and shall mentioned the CEIm, which approved the study.

Basic regulations of the trial

The Principal Investigator is obliged not to use, transmit to third parties, divulge and/or publish the results obtained in this trial without prior written consent from the Clínica Universidad de Navarra, sole sponsor of the trial. They should in any case respect the following conditions:

The results of this study may not be published until the end of the trial or before if both parties agree to do so.

a) The results of the trial and any proposal for publication shall be sent to the sponsor, at least 30 days before it is sent for publication. The sponsor should reply in writing within said period, granting said authorization or giving reasons for denying it. If no reply is received in this period, it shall be assumed that publication has been authorized.

b) The sponsor shall not cite the names of the investigators without their authorization, except when referring to work that is already published.

c) The sponsor shall permit publication of the data obtained in this trial in journals of recognized scientific prestige and the divulgation of its contents in seminars and conferences in the professional medical sphere, as long as the conditions in paragraphs a) and b) of this section are respected and if the final draft of the article may be may be reviewed within a maximum of thirty days. In any case, the legitimate interests of the trial sponsor shall be protected, such as in obtaining optimal protection of patients, coordination in the presentation of documents to medical authorities or other studies in progress in the same field, protection of confidential data and information, etc.

d) These regulations are understood to be applicable to information obtained in uncompleted trials or studies that were suspended before termination.

14. REFERENCES

1. Richards FO, Jr.: Upon entering an age of global ivermectin-based integrated mass drug administration for neglected tropical diseases and malaria. Malar J 2017, 16:168.
2. Frieman M, Yount B, Heise M, Kopecky-Bromberg SA, Palese P, Baric RS: Severe Acute Respiratory Syndrome Coronavirus ORF6 Antagonizes STAT1 Function by Sequestering Nuclear Import Factors on the Rough Endoplasmic Reticulum/Golgi Membrane. Journal of Virology 2007, 81:9812-24.
3. Zhou Y, Hou Y, Shen J, Huang Y, Martin W, Cheng F: Network-based drug repurposing for novel coronavirus 2019-nCoV/SARS-CoV-2. Cell Discovery 2020, 6:14.
4. Jang K-J, Jeong S, Kang DY, Sp N, Yang YM, Kim D-E: A high ATP concentration enhances the cooperative translocation of the SARS coronavirus helicase nsP13 in the unwinding of duplex RNA. Scientific Reports 2020, 10:4481.
5. Adedeji AO, Marchand B, Te Velthuis AJ, Snijder EJ, Weiss S, Eoff RL et al: Mechanism of nucleic acid unwinding by SARS-CoV helicase. PLoS One 2012, 7:e36521.
6. Mehta P, McAuley DF, Brown M, Sanchez E, Tattersall RS, Manson JJ et al: COVID-19: consider cytokine storm syndromes and immunosuppression. Lancet 2020.

7. Huang C, Wang Y, Li X, Ren L, Zhao J, Hu Y et al: Clinical features of patients infected with 2019 novel coronavirus in Wuhan, China. Lancet 2020, 395:497-506.
8. Omura S, Crump A: Ivermectin: panacea for resource-poor communities? Trends Parasitol 2014, 30:445-55.
9. Tay MY, Fraser JE, Chan WK, Moreland NJ, Rathore AP, Wang C et al: Nuclear localization of dengue virus (DENV) 1-4 non-structural protein 5; protection against all 4 DENV serotypes by the inhibitor Ivermectin. Antiviral Res 2013, 99:301-6.
10. Yang SNY, Atkinson SC, Wang C, Lee A, Bogoyevitch MA, Borg NA et al: The broad spectrum antiviral ivermectin targets the host nuclear transport importin alpha/beta1 heterodimer. Antiviral Res 2020:104760.
11. Wagstaff KM, Sivakumaran H, Heaton SM, Harrich D, Jans DA: Ivermectin is a specific inhibitor of importin alpha/beta-mediated nuclear import able to inhibit replication of HIV-1 and dengue virus. Biochem J 2012, 443:851-6.
12. Barrows NJ, Campos RK, Powell ST, Prasanth KR, Schott-Lerner G, Soto-Acosta R et al: A Screen of FDA-Approved Drugs for Inhibitors of Zika Virus Infection. Cell Host Microbe 2016, 20:259-70.
13. Varghese FS, Kaukinen P, Glasker S, Bespalov M, Hanski L, Wennerberg K et al: Discovery of berberine, abamectin and ivermectin as antivirals against chikungunya and other alphaviruses. Antiviral Res 2016, 126:117-24.
14. Mastrangelo E, Pezzullo M, De Burghgraeve T, Kaptein S, Pastorino B, Dallmeier K et al: Ivermectin is a potent inhibitor of flavivirus replication specifically targeting NS3 helicase activity: new prospects for an old drug. J Antimicrob Chemother 2012, 67:1884-94.
15. Lundberg L, Pinkham C, Baer A, Amaya M, Narayanan A, Wagstaff KM et al: Nuclear import and export inhibitors alter capsid protein distribution in mammalian cells and reduce Venezuelan Equine Encephalitis Virus replication. Antiviral Res 2013, 100:662-72.
16. Lee YJ, Lee C: Ivermectin inhibits porcine reproductive and respiratory syndrome virus in cultured porcine alveolar macrophages. Arch Virol 2016, 161:257-68.
17. Caly L, Druce J, Catton M, Jans D, KM W: The FDA-approved Drug Ivermectin inhibits the replication of SARS-CoV-2 in vitro. Antiviral Research 2020, In Press, Journal Pre-proof.
18. Yamasmith, E; et al. Efficacy and Safety of Ivermectin against Dengue Infection: A Phase III, Randomized, Double-blind, Placebo-controlled Trial, in he 34th Annual Meeting The Royal College of Physicians of Thailand- 'Internal Medicine and One Health'. 2018: Chonburi, Thailand. Registry available at: https://clinicaltrials.gov/ct2/show/NCT02045069 (accessed Aplri 5, 2020).
19. Lespine A, Alvinerie M, Sutra JF, Pors I, Chartier C: Influence of the route of administration on efficacy and tissue distribution of ivermectin in goat. Vet Parasitol 2005, 128:251-60.
20. Chiu SH, Lu AY: Metabolism and Tissue Residues. In: WC Campbell (ed),Ivermectin and Abamectin. New York: Springer-Verlag; 1989: 131–43.
21. Zhang X, Song Y, Ci X, An N, Ju Y, Li H et al: Ivermectin inhibits LPS-induced production of inflammatory cytokines and improves LPS-induced survival in mice. Inflamm Res 2008, 57:524-9.
22. Ci X, Li H, Yu Q, Zhang X, Yu L, Chen N et al: Avermectin exerts anti-inflammatory effect by downregulating the nuclear transcription factor kappa-B and mitogen-activated protein kinase activation pathway. Fundam Clin Pharmacol 2009, 23:449-55.
23. Blakley BR, Rousseaux CG: Effect of ivermectin on the immune response in mice. Am J Vet Res 1991, 52:593-5.

24. MSD: Stromectol label approved in The Netherlands. Available at https://www.geneesmiddeleninformatiebank.nl/smpc/h28341_smpc.pdf. 2015.
25. Infectopharm: Scabioral 3mg package insert available here: https://www.infectopharm.com/images/newsletter/1216/fi-scabioral.pdf (accessed JUly 2019).
26. Gonzalez Canga A, Sahagun Prieto AM, Diez Liebana MJ, Fernandez Martinez N, Sierra Vega M, Garcia Vieitez JJ: The pharmacokinetics and interactions of ivermectin in humans--a mini-review. AAPS J 2008, 10:42-6.
27. Merck_&_Co.: Stromectrol. FDA approved Package insert 2009. http://www.accessdata.fda.gov/drugsatfda_docs/label/2009/050742s026lbl.pdf (Accessed February 2018).
28. Merck_&_Co.: Stromectrol. TGA-Australia approved Package insert 2014. https://www.ebs.tga.gov.au/ebs/picmi/picmirepository.nsf/pdf?OpenAgent&id=CP-2011-PI-02659-3&d=2016071016114622483 (Accessed February 2018).
29. Elkassaby MH: Ivermectin uptake and distribution in the plasma and tissue of Sudanese and Mexican patients infected with Onchocerca volvulus. Trop Med Parasitol 1991, 42:79-81.
30. Kamgno J, Gardon J, Gardon-Wendel N, Demanga N, Duke BO, Boussinesq M: Adverse systemic reactions to treatment of onchocerciasis with ivermectin at normal and high doses given annually or three-monthly. Trans R Soc Trop Med Hyg 2004, 98:496-504.
31. Smit MR, Ochomo EO, Aljayyoussi G, Kwambai TK, Abong'o BO, Chen T et al: Safety and mosquitocidal efficacy of high-dose ivermectin when co-administered with dihydroartemisinin-piperaquine in Kenyan adults with uncomplicated malaria (IVERMAL): a randomised, double-blind, placebo-controlled trial. Lancet Infect Dis 2018, 18:615-26.
32. Awadzi K, Opoku NO, Addy ET, Quartey BT: The chemotherapy of onchocerciasis. XIX: The clinical and laboratory tolerance of high dose ivermectin. Trop Med Parasitol 1995, 46:131-7.
33. Awadzi K, Attah SK, Addy ET, Opoku NO, Quartey BT: The effects of high-dose ivermectin regimens on Onchocerca volvulus in onchocerciasis patients. Trans R Soc Trop Med Hyg 1999, 93:189-94.
34. Guzzo CA, Furtek CI, Porras AG, Chen C, Tipping R, Clineschmidt CM et al: Safety, tolerability, and pharmacokinetics of escalating high doses of ivermectin in healthy adult subjects. J Clin Pharmacol 2002, 42:1122-33.
35. Munoz J, Ballester MR, Antonijoan RM, Gich I, Rodriguez M, Colli E et al: Safety and pharmacokinetic profile of fixed-dose ivermectin with an innovative 18mg tablet in healthy adult volunteers. PLoS Negl Trop Dis 2018, 12:e0006020.
36. Gardon J, Gardon-Wendel N, Demanga N, Kamgno J, Chippaux JP, Boussinesq M: Serious reactions after mass treatment of onchocerciasis with ivermectin in an area endemic for Loa loa infection. Lancet 1997, 350:18-22.
37. Nicolas P, Maia MF, Bassat Q, Kobylinski KC, Monteiro W, Rabinovich NR et al: Safety of oral ivermectin during pregnancy: a systematic review and meta-analysis. Lancet Glob Health 2020, 8:e92-e100.
38. European Medicines Agency. Guidance on the Management of Clinical Trials during the COVID-19 (Coronavirus) pandemic Version 2. Available at: https://ec.europa.eu/health/sites/health/files/files/eudralex/vol-10/guidanceclinicaltrials_covid19_en.pdf (Accessed April 11, 2020).

ANNEX 1: SCHEDULE OF VISITS AND EVALUATIONS

	**Recruitment** Day 0	**Visit 2** Day 4 post treatment	**Visit 3** Day 7 post treatment	**Visit 4** Day 14 post treatm.	**Visit 5** Day 21 post treatm.	**Final visit** Day 28 post treatm.
Informed consent#	X					
Randomization	X					
Clinical data collection	X	X	X	X	X	X
Physical examination	X	X	X	X	X	X
Laboratory test*	X		X	X		
Urine pregnancy test	X					
Nasopharyngeal swab (PCR for SARS-CoV-2)	X	X	X	X	X	
Serology for SARS-CoV-2	X	X	X	X	X	X
Proinflammatory and activation markers	X	X	X	X	X	X
Cell responses (PBMC)	X		X			X
Chest x-ray	X					
Administration of IP	X					
Daily self-assessment of symptoms	X--X					
Evaluation of adverse events	X--X					

# verbal consent initially, in writing when isolation is concluded
* C reactive protein, Procalcitonin, Ferritin, IL-6
, Renal function: urea, creatinine, electrolytes, FBC,
Troponin T, CPK, DD, LDH

ANNEX 2: DISCRETE DOSING TABLE

**# of 3mg tablets-->**	**6**	**7**	**8**	**9**	**10**	**11**	**12**	**13**	**14**	**15**
**Dose in mg -->**	**18**	**21**	**24**	**27**	**30**	**33**	**36**	**39**	**42**	**45**
**45**	400	467	533	600	667	733	800	867	933	1000
**46**	391	457	522	587	652	717	783	848	913	978
**47**	383	447	511	574	638	702	766	830	894	957
**48**	375	438	500	563	625	688	750	813	875	938
**49**	367	429	490	551	612	673	735	796	857	918
**50**	360	420	480	540	600	660	720	780	840	900
**51**	353	412	471	529	588	647	706	765	824	882
**52**	346	404	462	519	577	635	692	750	808	865
**53**	340	396	453	509	566	623	679	736	792	849
**54**	333	389	444	500	556	611	667	722	778	833
**55**	327	382	436	491	545	600	655	709	764	818
**56**	321	375	429	482	536	589	643	696	750	804
**57**	316	368	421	474	526	579	632	684	737	789
**58**	310	362	414	466	517	569	621	672	724	776
**59**	305	356	407	458	508	559	610	661	712	763
**60**	300	350	400	450	500	550	600	650	700	750
**61**	295	344	393	443	492	541	590	639	689	738
**62**	290	339	387	435	484	532	581	629	677	726
**63**	286	333	381	429	476	524	571	619	667	714
**64**	281	328	375	422	469	516	563	609	656	703
**65**	277	323	369	415	462	508	554	600	646	692
**66**	273	318	364	409	455	500	545	591	636	682
**67**	269	313	358	403	448	493	537	582	627	672
**68**	265	309	353	397	441	485	529	574	618	662
**69**	261	304	348	391	435	478	522	565	609	652
**70**	257	300	343	386	429	471	514	557	600	643
**71**	254	296	338	380	423	465	507	549	592	634
**72**	250	292	333	375	417	458	500	542	583	625
**73**	247	288	329	370	411	452	493	534	575	616
**74**	243	284	324	365	405	446	486	527	568	608
**75**	240	280	320	360	400	440	480	520	560	600
**76**	237	276	316	355	395	434	474	513	553	592
**77**	234	273	312	351	390	429	468	506	545	584
**78**	231	269	308	346	385	423	462	500	538	577
**79**	228	266	304	342	380	418	456	494	532	570
**80**	225	263	300	338	375	413	450	488	525	563
**81**	222	259	296	333	370	407	444	481	519	556
**82**	220	256	293	329	366	402	439	476	512	549
**83**	217	253	289	325	361	398	434	470	506	542
**84**	214	250	286	321	357	393	429	464	500	536
**85**	212	247	282	318	353	388	424	459	494	529
**86**	209	244	279	314	349	384	419	453	488	523
**87**	207	241	276	310	345	379	414	448	483	517
**88**	205	239	273	307	341	375	409	443	477	511
**89**	202	236	270	303	337	371	404	438	472	506
**90**	200	233	267	300	333	367	400	433	467	500
**91**	198	231	264	297	330	363	396	429	462	495
**92**	196	228	261	293	326	359	391	424	457	489
**93**	194	226	258	290	323	355	387	419	452	484
**94**	191	223	255	287	319	351	383	415	447	479
**95**	189	221	253	284	316	347	379	411	442	474
**96**	188	219	250	281	313	344	375	406	438	469
**97**	186	216	247	278	309	340	371	402	433	464
**98**	184	214	245	276	306	337	367	398	429	459
**99**	182	212	242	273	303	333	364	394	424	455
**100**	180	210	240	270	300	330	360	390	420	450
