# Supplementary material for: The effect of early treatment with ivermectin on viral load, symptoms and humoral response in patients with non-severe COVID-19: A pilot, double-blind, placebo-controlled, randomized clinical trial
Source: eClinicalMedicine. 2021 Jan 19;32:100720. doi: 10.1016/j.eclinm.2020.100720 (PMC7816625; doi:10.1016/j.eclinm.2020.100720)
Supplement: Supplementary file 4 [file mmc4.pdf]

CLINICAL TRIAL STATISTICAL ANALYSIS PLAN

Code: SAINT

Title of trial: Pilot study to evaluate the potential of ivermectin to reduce COVID-19 transmission

Phase: IIa

Date of statistical analysis plan: 5 of October 2020 - Version 1.0

EudraCT number: 2020-001474-29

Sponsor: Clínica Universidad de Navarra

Written by: Aina Casellas, ISGlobal

Principal Investigator: Dr. Carlos Chaccour

Clínica Universidad de Navarra

CONFIDENTIAL MAY NOT BE USED, DIVULGED OR PUBLISHED WITHOUT TRIAL SPONSOR'S CONSENT.

CONFIDENTIAL

Approval Page

The signatures below confirm that the signatories have reviewed and approved this document which shall govern the stipulated statistical analysis tasks of the specified research study.

Aina Casellas	Head of Statistics
**Name**	**Designation**
	5 of October 2020
**Signature**	**Date**
Sergi Sanz	Reviewer
**Name**	**Designation**
	5 of October 2020
**Signature**	**Date**
Carlos Chaccour	Approver
**Name**	**Designation**
	5 of October 2020
**Signature**	**Date**

CONFIDENTIAL

1 Table of Contents

1 Table of Contents 3
2 Abbreviations and Definitions 5
3 Introduction 5
3.1 Preface 5
3.2 Purpose of the analyses 6
4 Study Objectives and Endpoints 6
4.1 Main Study Objective 6
4.2 Secondary Study Objectives 6
4.3 Endpoints 6
4.3.1 Primary 6
4.3.2 Secondary 6
5 Study Methods 7
5.1 General Study Design and Plan 7
5.2 Equivalence or Non-Inferiority Studies 7
5.3 Inclusion-Exclusion Criteria and General Study Population 8
5.3.1 Inclusion criteria 8
5.3.2 Exclusion criteria 8
5.3.3 Criteria for withdrawal 9
5.4 Randomisation and Blinding 9
5.5 Study Variables 9
5.5.1 Baseline information (day 1) 10
5.5.2 Previous medical history (day 1) 10
5.5.3 Visit information 11
5.5.4 Concomitant medication (during follow up) 15
5.5.5 Adverse Events (during follow up) 15
6 Sample Size 18
7 General Considerations 19
7.1 Timing of Analyses 19
7.1.1 Full Analysis Population 19
7.1.2 Safety Population 19
7.2 Covariates and Subgroups 19
7.3 Missing Data 19

CONFIDENTIAL

7.4 Multiple Testing 19
8 Summary of Study Data..... 19
8.1 Subject Disposition 20
8.2 Derived variables..... 21
8.3 Concurrent Illnesses and Medical Conditions 21
8.4 Prior and Concurrent Medications..... 21
8.5 Treatment Compliance..... 21
9 Efficacy Analyses 21
9.1 Primary Efficacy Analysis..... 21
9.2 Secondary Efficacy Analyses..... 22
10 Safety Analyses..... 22
10.1 Extent of Exposure 22
10.2 Adverse Events 22
10.3 Deaths, Serious Adverse Events and other Significant Adverse Events..... 22
10.4 Pregnancies 22
10.5 Clinical Laboratory Evaluations 22
11 Pharmacokinetics 23
12 Reporting Conventions..... 23
13 Technical Details..... 23
14 References..... 24
15 Listing of Tables, Listings and Figures 25
15.1 Tables 25
15.2 Listings..... 29
15.3 Figures 30

CONFIDENTIAL

2 Abbreviations and Definitions

AE	Adverse Event
BMI	Body Mass Index
CI	Confidence interval
CRF	Case Report Form
Ct	Cycle threshold
CUN	Clínica Universidad de Navarra
ENT	Ear, Nose, Throat
FU	Follow-up
LFU	Lost to follow-up
mITT	Modified Intention to treat
NTDs	Neglected tropical diseases
PP	Per Protocol
RR	Relative risk ratio
RRP	Reports reported by patients
SAE	Severe Adverse Event
SAP	Statistical Analysis Plan

3 Introduction

3.1 Preface

As of May 8, 2020, there were a more than 4 million cases of COVID-19 and more than 270,000 deaths worldwide. There is currently no proven treatment for this disease, so drug-based strategies to control COVID-19 are urgently needed. Among the potential approaches to be considered for COVID-19 are early treatment of patients to reduce progression to severe disease, treatment to block or reduce transmission with a drug that reduces viral excretion in the airway, prophylaxis or combinations of either. These strategies could significantly relieve pressure on the health system and decrease indirect deaths due to other diseases requiring intensive care.

Ivermectin is a widely used anti-parasitic drug for the treatment and control of neglected tropical diseases (NTDs) which has shown an excellent safety profile, with more than 2.5 billion doses distributed in the last 30 years. Based on previous evidence, ivermectin could be active against COVID-19 by inhibiting the replication of the virus or by having immunomodulatory effects.

CONFIDENTIAL

3.2 Purpose of the analyses

These analyses will assess the efficacy and safety of ivermectin as a treatment for COVID-19 patients to reduce virus spread.

4 Study Objectives and Endpoints

4.1 Main Study Objective

To determine the efficacy of a single dose of ivermectin, administered to low risk, non-severe COVID-19 patients in the first 48 hours after symptoms onset to reduce the proportion of patients with detectable SARS-CoV-2 RNA by PCR from nasopharyngeal swab at day seven post-treatment.

4.2 Secondary Study Objectives

1. To assess the efficacy of ivermectin to reduce the SARS-CoV-2 viral load in the nasopharyngeal swab at day seven post treatment
2. To assess the efficacy of ivermectin to improve symptom progression in treated patients
3. To assess the proportion of seroconversions at day 21 in treated patients
4. To assess the safety of ivermectin at the proposed dose
5. To determine the magnitude of immune response against SARS-CoV-2
6. To assess the early kinetics of immunity against SARS-CoV-2

4.3 Endpoints

4.3.1 Primary

Proportion of patients with a positive SARS-CoV-2 PCR from a nasopharyngeal swab at day 7 post-treatment.

4.3.2 Secondary

1. Mean viral load as determined by PCR cycle threshold (Ct) at baseline and on days 4, 7, 14 and 21.
2. Proportion of patients with fever and cough at days 4, 7, 14 and 21 as well as proportion of patients progressing to severe disease or death during the trial.
3. Proportion of patients with seroconversion at day 21.
4. Proportion of drug-related adverse events during the trial.
5. Median levels of IgG, IgM, IgA measured by Luminex, frequencies of innate and SARS-CoV-2-specific T cells assessed by flow cytometry, median levels of inflammatory and activation markers measured by Luminex and transcriptomics.
6. Median kinetics of IgG, IgM, IgA levels till day 28.

The analysis of objectives 5 and 6 will be addressed in a supplementary SAP.

CONFIDENTIAL

5 Study Methods

5.1 General Study Design and Plan

The SAINT trial is a single center, randomized, double-blind, placebo-controlled, parallel arm, superiority clinical trial.

Participants will be randomized to receive a single dose of 400 mcg/kg ivermectin or a placebo equivalent and followed up for 28 days.

A final study visit will be made for participants who withdraw prematurely from the study or are withdrawn by the investigator. A summary of the study visits and procedures is provided in **Table 1Table 1.** Visit Procedures below.

	**Recruitment** Day 1	**Visit 2** Day 4	**Visit 3** Day 7	**Visit 4** Day 14	**Visit 5** Day 21	**Final visit** Day 28
Informed consent#	X					
Randomization	X					
Clinical data collection	X	X	X	X	X	X
Physical examination	X	X	X	X	X	X
Laboratory test*	X		X	X		
Urine pregnancy test	X					
Nasopharyngeal swab (PCR for SARS-CoV-2)	X	X	X	X	X	
Serology for SARS-CoV-2	X	X	X	X	X	X
Proinflammatory and activation markers	X	X	X	X	X	X
Cell responses (PBMC)	X		X			X
Chest x-ray	X					
Administration of IP	X					
Daily self-assessment of symptoms	X---X					
Evaluation of adverse events	X---X					

# verbal consent initially, in writing when isolation is concluded

* C reactive protein, Procalcitonin, Renal function: urea, creatinine, electrolytes, FBC, Troponin T, CPK, DD, LDH

***Table 1**. Visit Procedures*

5.2 Equivalence or Non-Inferiority Studies

Not Applicable

CONFIDENTIAL

5.3 Inclusion-Exclusion Criteria and General Study Population

5.3.1 Inclusion criteria

- Patients diagnosed with COVID-19 in the emergency room of the Clínica Universidad de Navarra with a positive SARS-CoV-2 PCR.
- Residents of the Pamplona basin ("Cuenca de Pamplona")
- The patient should be between the ages of 18 and 60 years
- Negative pregnancy test for women of child bearing age*
- The patient or his/her representative have given consent to participate in the study.
- The patient should, in the investigator's opinion, be able to comply with all the requirements of the clinical trial (including home follow up during isolation)

*Women of child bearing age may participate if they use a safe contraceptive method for the entire period of the study and at least one month afterwards. A woman is considered to not have childbearing capacity if she is post-menopausal (minimum of 2 years without menstruation) or has undergone surgical sterilization (at least one month before the study).

5.3.2 Exclusion criteria

- Known history of Ivermectin allergy
- Hypersensitivity to any component of Stromectol®
- COVID-19 Pneumonia
    - Diagnosed by the attending physician
    - Identified in a chest X-ray
- Fever or cough present for more than 72 hours
- Positive IgG against SARS-CoV-2 by rapid test
- Age under 18 or over 60 years
- The following co-morbidities (or any other disease that might interfere with the study in the eyes of the investigator):
    - Immunosuppression
    - Chronic Obstructive Pulmonary Disease
    - Diabetes
    - Hypertension
    - Obesity
    - Acute or chronic renal failure
    - History of coronary disease
    - History of cerebrovascular disease
    - Current neoplasm
- Recent travel history to countries that are endemic for Loa loa (Angola, Cameroon, Central African Republic, Chad, Democratic Republic of Congo, Ethiopia, Equatorial, Guinea, Gabon, Republic of Congo, Nigeria and Sudan)
- Current use of CYP 3A4 or P-gp inhibitor drugs such as quinidine, amiodarone, diltiazem, spironolactone, verapamil, clarithromycin, erythromycin, itraconazole, ketoconazole, cyclosporine, tacrolimus, indinavir, ritonavir or cobicistat. Use of critical CYP3A4 substrate drugs such as warfarin.

CONFIDENTIAL

5.3.3 Criteria for withdrawal

Patients will be informed that they may be withdrawn from the study at any time without providing any explanation. Withdrawal may also take place for any of the following reasons:

- Adverse reaction that puts the patient's life at risk, according the principal investigator's judgment.
- Decision made by the patient or the legally authorized person.
- Documented progression of the disease requiring hospitalization in a different center.
- The patient cannot complete the trial visits due to unforeseen circumstances.
- Development of other disorders that, in the opinion of the investigator and in the patient's best interest, make it advisable for him/her to leave the study.
- Administration of treatment with a non-permitted drug.
- The investigator believes that the patient may benefit from a different treatment.
- Loss of monitoring

The investigator shall strive to ensure that the patient agrees to the final trial visit. The data of the withdrawn patients shall be collected up to this final visit. If the withdrawal is the outcome of a Serious adverse event related to ivermectin or SARS-CoV-2, the data shall be collected until the patient recovers or stabilizes.

The subjects shall be replaced if the withdrawal is for a reason different from lack of efficacy of the treatment or appearance of adverse events. All efforts will be made to maintain the total number of assessable patients as per the protocol.

The withdrawal shall be documented in the patient's clinical history, giving the reasons for the decision. A final trial visit should be conducted by the clinical team if possible.

5.4 Randomisation and Blinding

Participants will be randomized to receive a single dose of 400 mcg/kg ivermectin or a placebo equivalent. The randomization code will be generated by the trial statistician using blocks of four individuals that ensure balance between the groups. This randomization will be performed with the module *ralloc* [1] for Stata software [2]. A study identification code with the format “SAINT-##” (##: from 01 to 24) will be generated using a sequence of random numbers so that the randomization number does not match the subject identifier.

The sequence and code used will be kept in an encrypted file accessible only to the trial statistician. A physical copy will be kept on a locked cabinet at the CUN, accessible only to the staff administering the drug who will not enroll or attend to patient care.

This is a double-blind study; the participants and the clinical team will be blinded. The placebo will not be visibly identical, but it will be administered by a researcher not involved in the clinical care or participant follow up.

5.5 Study Variables

The list of variables collected at each study timepoint is provided in the following subsections.

CONFIDENTIAL

All study visits must be on the planned days, so no visits out of the specified visit days (1, 4, 7, 14, 21 and 28) will be taken into account in the analysis.

5.5.1 Baseline information (day 1)

The variables listed below will be assessed on the first visit.

Variable name	Description	Data type	Coding or range
**Demographics**			
Age	Age (years)	Integer	18-60
Sex	Gender	Integer	1: Female<br>2: Male
V1_VSHeight	Height (in m)	Real	1.2-2.2
V1_VSWeight	Weight (in kg)	Real	40-100
V1_VSBMI	BMI (in kg/m^2)	Real	18-30
**Smoking habits and alcohol consumption**			
Smoke	Smoker or tobacco products user	Integer	0: No<br>1: Yes
Cigarettes	Number of cigarettes per day	Integer	0-40
SmokeYears	Smoking duration (in years)	Integer	??
Alcohol	Alcohol consumption	Integer	0: No<br>1: Yes
AlcoholUnits	Weekly alcohol consumption (in units)	Integer	0-20
**Medication**			
MedicationTaken	Taking medication	Integer	0: No<br>1: Yes

5.5.2 Previous medical history (day 1)

For each preexisting medical condition, the list of variables given below will be collected.

Variable name	Description	Data type	Coding or range
RELEVANT_HISTORY_MEDICAL	Relevant History Medical	Integer	0: Other<br>1: Cardiovascular<br>2: Respiratory<br>3: Hepato-biliary<br>4: Gastro-intestinal<br>5: Genito-urinary<br>6: Endocrine<br>7: Hematological<br>8: Musculo-skeletal<br>9: Neoplasia<br>10: Neurological<br>11: Psychological

CONFIDENTIAL

			12: Immunological<br>13: Dermatological<br>14: Allergies<br>15: Eyes, ear, nose, throat
Details	Details (Diagnosis / Procedure)	String	
StartDate	Start Date	Date	
Ongoing	Is the medical condition ongoing?	Integer	0: No<br>1: Yes
EndDate	End Date	Date	

5.5.3 Visit information

The list below contains those variables assessed recurrently during follow up. Note that not all variables are collected every visit. A superscript indicating the days when the information is collected is provided for those variables not collected at every study visit.

Variable name	Description	Data type	Coding or range
VIVisitDate	Visit Date	Date	
ASAnySymptoms	Does the patient have any of the symptoms suggestive of COVID-19?	Integer	0: No<br>1: Yes
ASAnyNewSymptoms	Does the patient have any new symptom suggestive of COVID-19?	Integer	0: No<br>1: Yes
SYMPTOM	Symptom	Integer	1: General<br>2: ENT<br>3: Respiratory<br>4: Digestive<br>5: Other
VSHeartRate	Heart rate (beats per minute)	Integer	50-120
VSBloodPressSystolic	Systolic blood pressure (mmHg)	Integer	90-160
VSBloodPressDiastolic	Diastolic blood pressure (mmHg)	Integer	50-100
VSTemperature	Temperature (ºC)	Real	35-41
VSOxygenSaturation	Oxygen saturation (%)	Real	85-
VSRespiratoryRate	Respiratory rate (breaths per minute)	Integer	8-24
VSHeight	Height (m)	Real	1.2-2.2
VSWeight	Weight (kg)	Real	40-100
VSBMI	BMI (kg/m^2)	Real	18-30

CONFIDENTIAL

PHEAnyAbnormalPhyExam	Does the patient show any abnormal signs on physical examination?	Integer	0: No<br>1: Yes
PHEAnyNewAbnormalPhyExam	Does the patient show any new abnormal signs on physical examination?	Integer	0: No<br>1: Yes
Xray [1]	X-RAY Result	Integer	0: Normal<br>1: Abnormal
ChestXrayDescription [1]	X-RAY Description:	String	
PregnancyTestConducted [1]	Was a pregnancy test conducted?	Integer	0: No<br>1: Yes<br>2: NA
PregnancyTestResult [1]	Pregnancy test result	Integer	0: Negative<br>1: Positive
PCRSwabResults [1,4,7,14,21]	Swab result	Integer	0: Negative<br>1: Positive
RSCompleteSelfAssessment [4,7,14,21,28]	Did the participant complete the self-assessment daily since the last visit?	Integer	0: No<br>1: Yes
RSParticipantReportAE [4,7,14,21,28]	Did the participant report any negative changes?	Integer	0: No<br>1: Yes
RSParticipantReportCM [4,7,14,21,28]	Did the participant report any new medications?	Integer	0: No<br>1: Yes
**Nasopharyngeal swab (PCR for SARS-CoV-2)** [1,4,7,14,21]			
PCRPCR_gen_N	Gene N (PCR sample) – Result	Integer	1: Detected<br>2: Not detected<br>3: Not done
PCRPCR_muestra_CT_N	Gene N (PCR sample) – Ct	Real	12-40 (41 if not detected)
PCRPCR_muestra_Q_N	Gene N (PCR sample) – Quantity (Copies/ml)	Integer	$0\text{-}10^{12}$
PCRPCR_gen_E	Gene E (PCR sample) – Result	Integer	1: Detected<br>2: Not detected<br>3: Not done
PCRPCR_muestra_CT_E	Gene E (PCR sample) – Ct	Real	12-40 (41 if not detected)
PCRPCR_muestra_Q_E	Gene E (PCR sample) – Quantity (Copies/ml)	Integer	$0\text{-}10^{12}$
PCRPCR_gen_ab1	Gene ab1 (PCR sample) – Result	Integer	1: Detected<br>2: Not detected<br>3: Not done
PCRPCR_muestra_CT_ab1	Gene ab1 (PCR sample) – Ct	Real	12-40 (41 if not

CONFIDENTIAL

			detected)
PCRPCR_muestra_Q_ab1	Gene ab1 (PCR sample) – Quantity (Copies/ml)	Integer	$0\text{-}10^{12}$
PCRPCR_cul_N	Gene N (PCR culture) – Result	Integer	1: Detected<br>2: Not detected<br>3: Not done
PCRPCR_cultivo_CT_N	Gene N (PCR culture) – Ct	Real	12-40 (41 if not detected)
PCRPCR_cultivo_Q_N	Gene N (PCR culture) – Quantity (Copies/ml)	Integer	$0\text{-}10^{12}$
PCRPCR_cul_E	Gene E (PCR culture) – Result	Integer	1: Detected<br>2: Not detected<br>3: Not done
PCRPCR_cultivo_CT_E	Gene E (PCR culture) – Ct	Real	12-40 (41 if not detected)
PCRPCR_cultivo_Q_E	Gene E (PCR culture) – Quantity (Copies/ml)	Integer	$0\text{-}10^{12}$
PCRPCR_cul_ab1	Gene ab1 (PCR culture) – Result	Integer	1: Detected<br>2: Not detected<br>3: Not done
PCRPCR_cultivo_CT_ab1	Gene ab1 (PCR culture) – Ct	Real	12-40 (41 if not detected)
PCRPCR_cultivo_Q_ab1	Gene ab1 (PCR culture) – Quantity (Copies/ml)	Integer	$0\text{-}10^{12}$
**Laboratory test** [1,7,14]			
BIOCHEMCreatinine	Blood creatinine (mg/dL)	Real	Male: 0.7-1.2<br>Female: 0.5-0.9
BIOCHEMUreaBlood	Urea blood (mg/dL)	Integer	16.6-48.5
BIOCHEMMDRD	MDRD (mL/min/1.73m^2)	Real	60-
BIOCHEMLDH	LDH (Ul/L)	Real	135-225
BIOCHEMCRP	CRP (mg/dL)	Real	0-0.5
BIOCHEMTroponin	Troponin T (ng/L)	Real	0-13
BIOCHEMProcalcitonin	Procalcitonin (ng/mL)	Real	0-0.5
BIOCHEMSodium	Sodium (mEq/L)	Real	136-145
BIOCHEMPotassium	Potassium (mEq/L)	Real	3.5-5
BIOCHEMChlorine	Chlorine (mEq/L)	Real	98-107
BIOCHEMBicarbonate	Bicarbonate (mEq/L)	Real	22-29
BIOCHEMRemAnion	Remaining anion (mEq/L)	Real	8-16
BIOCHEMFerritin	Ferritin (ng/mL)	Real	Male: 30-400<br>Female: 15-150
BIOCHEMCPK	CPK (Ul/L)	Real	Male: 0-190

CONFIDENTIAL

			Female: 0-170
BIOCHEMIL6	IL-6 (pg/mL)	Real	0-7
COAGDimero	D-Dimer (ng/mL)	Real	150-500
BCRBC	Red blood cells (10^{12}/L)	Real	Male: 4.7-5.1<br>Female: 4.2-5.4
BCHb	Haemoglobin (g/dL)	Real	Male: 14-17<br>Female: 12-16
BCHto	Hto (%)	Real	Male: 42-49<br>Female: 37-48
BCVCM	VCM (fL)	Real	80-95
BCHCM	HCM (pg)	Real	27-31
BCCMHC	CMHC (g/dL)	Real	33-35
BCRDW	RDW (%)	Real	11.5-14.5
BCPlaquetas	Platelets (10^9/L)	Integer	150-450
BCVPM	VPM (f/L)	Real	7.2-11.1
BCPDW	PDW (%)	Real	25-65
BCPTC	PTC (%)	Real	0.12-0.36
BCWBC	White blood cells (10^9/L)	Real	4.8-10.8
BCNeutrophils	Neutrophils (%)	Real	45-75
BCTotalNeutrophils	Total Neutrophils (10^9/L)	Real	1.5-6.5
BCLymphocytes	Lymphocytes (%)	Real	20-50
BCTotalLymphocytes	Total Lymphocytes (10^9/L)	Real	1.2-4
BCMonocytes	Monocytes (%)	Real	2-9
BCTotalMonocytes	Total Monocytes (10^9/L)	Real	0.4-0.8
BCEosinophils	Eosinophils (%)	Real	0-6
BCTotalEosinophils	Total Eosinophils (10^9/L)	Real	0-0.4
BCBasophils	Basophils (%)	Real	0-4
BCTotalBasophils	Total Basophils (10^9/L)	Real	0-0.2
**Serology**			
SEROLOGYSerSarsCoV2 [1,21]	SARS-CoV-2 (COVID-19) Serology result	Integer	0: Negative<br>1: Positive
BIOBANKEDTA5	EDTA 5 Biobank collected	Integer	0: No<br>1: Yes
BIOBANKEDTA5_PBMC	EDTA 5 Biobank (PBMC) collected	Integer	0: No<br>1: Yes
BIOBANKSerumBiobank	Serum 10 Biobank collected	Integer	0: No<br>1: Yes
BIOBANKHeparinBiobank	Heparin 10 Biobank collected	Integer	0: No<br>1: Yes

CONFIDENTIAL

Superscripts indicate the days in which each variable is collected. Variables with no superscripts are collected at all visits.
ENT: Ear, Nose, Throat.

5.5.4 Concomitant medication (during follow up)

Details on concomitant treatments will be collected during the study.

Variable name	Description	Data type	Coding or range
Medication	Medication	String	
Units	Units	String	
FREQ	Frequency	String	
TotalDailyDose	Todal daily dose	Integer	
Reason	Reason	String	
StartDate	Start Date	Date	
EndDate	End Date	Date	
CMContinuing	Continuing at end of trial?	Integer	0: No<br>1: Yes

5.5.5 Adverse Events (during follow up)

For each Adverse Event occurred, the following details will be recorded.

Variable name	Description	Data type	Coding or range
AE	Adverse Event name	String	
StartDate	Start Date	Date	
TimeStart	Start Time	Date	
Ongoing	Is the Adverse Event ongoing?	Integer	0: No<br>1: Yes
EndDate	End Date	Date	
TimeEnd	End Time	Date	
Outcome	Outcome	Integer	1: Recovered<br>2: Recovered with sequelae<br>3: Continuing<br>4: Patient Died<br>5: Change in AE<br>6: unknown
Severity	Severity	Integer	1: Mild<br>2: Moderate<br>3: Severe
RelationshipStudyDrug[1]	Plausible relationship to Study Drug	Integer	0: No<br>1: Yes
ActionTaken	Action taken with Study Drug	Integer	1: None

CONFIDENTIAL

			2: Dose Reduction Temporarily<br>3: Dose Reduced<br>4: Discontinued Temporarily<br>5: Discontinued
WithdrawnDueAE	Withdrawn due to an adverse event?	Integer	0: No<br>1: Yes
SAE	Serious adverse event (SAE)?	Integer	0: No<br>1: Yes
SAEReporting	If SAE does it require immediate reporting?	Integer	0: No<br>1: Yes

1: An event will be considered as related to the study drug if it appears within 5 days after its administration.

5.5.6 Daily self-assessment of symptoms

Each patient will give information on the variables below each day during follow up.

Variable name	Description	Data type	Coding or range
day	Follow up day	Integer	1-28
como_se_encuentra	How do you fell today?	Integer	1: Better than yesterday<br>2: Same as yesterday<br>3: Worse than yesterday
malestar_si_no	Are you feeling generally unwell or tired?	Integer	0: No<br>1: Yes
malestar_mejor	If feeling generally unwell or tired, is it:	Integer	1: Better than yesterday<br>2: Same as yesterday<br>3: Worse than yesterday
temp_si_no	Have you taken your body temperature?	Integer	0: No<br>1: Yes
temp	What has been your highest temperature since yesterday? (in ºC)	Real	35-41
fiebre	Have you had a fever since yesterday?	Integer	0: No<br>1: Yes
tos_si_no	Do you have a cough?	Integer	0: No<br>1: Yes
tos_es	Your cough is:	Integer	1: Dry<br>2: Productive
tos_expectoracion	Your expectoration:	Integer	1: Is whitish<br>2: Is yellowish<br>3: Is greenish<br>4: Has blood

CONFIDENTIAL

tos_esta	Your cough is:	Integer	1: Better than yesterday<br>2: Same as yesterday<br>3: Worse than yesterday
tos_esta	Does your cough make it difficult for you to eat, drink or talk?	Integer	0: No<br>1: Yes
olores	Can you perceive odours well?	Integer	0: No<br>1: Yes
mal_sabor	Do you have a bad taste in your mouth?	Integer	0: No<br>1: Yes
cefalea	Cephalea	Integer	0: No<br>1: Yes
dolor_cabeza	Do you have a headache?	Integer	0: No<br>1: Yes
dolor_garganta	Do you have a sore throat?	Integer	0: No<br>1: Yes
congestion_si_no	Do you have a stuffy nose?	Integer	0: No<br>1: Yes
fatiga_si_no	Do you have fatigue? (Shortness of breath?)	Integer	0: No<br>1: Yes
fatiga	You are fatigated: (multiple choice)	Integer	1: Resting<br>2: When talking<br>3: When walking<br>4: When climbing stairs
vomitos_si_no	Do you have vomiting?	Integer	0: No<br>1: Yes
casados_por_tos	Is it caused by coughing?	Integer	0: No<br>1: Yes
veces_vomitado	How many times have you vomited since yesterday?	Integer	1-
diarrea_si_no	Do you have diarrhea?	Integer	0: No<br>1: Yes
deposiciones	How many bowel movements have you had since yesterday?	Integer	1-
medicacion_si_no	Have you taken any medication for these symptoms in the last 24 hours?	Integer	0: No<br>1: Yes
medicacion_cual	If yes, which one?	String	
sintomas_1	Since yesterday, have you experienced any of these symptoms? (multiple choice)	Integer	1: Confusion / difficulty concentrating on something<br>2: Dizziness<br>3: Drowsiness<br>4: Vertigo

CONFIDENTIAL

			5: Tremors
sintomas_2	Since yesterday, have you experienced any of these symptoms? (multiple choice)	Integer	1: Blurry vision<br>2: Difficulty focusing on objects or reading<br>3: Tunnel vision<br>4: Abnormal colors or shapes<br>5: Blind spots<br>6: Floating points
sintomas_3	Since yesterday, have you experienced any of these symptoms? (multiple choice)	Integer	1: Pruritus (itching)<br>2: Rash

6 Sample Size

The study will enroll 24 participants, 12 per arm. The sample size has been calculated for comparing two proportions. This sample provides 80% power at 5% significance level using Fisher´s unilateral test to determine a difference of 45% (100% vs 55%) in the proportion of participants with positive PCR at day 7. This includes up to one participant lost to follow up (LFU) per arm. We estimated this low LFU level given that the patients will be isolated at home and will have a low risk of progression to severe disease. The 100% positivity rate at day seven is based on the clinical experience at the Clínica Universidad de Navarra. **Figure 1** provides the relationship power-outcome measure for the full sample and one scenario considering LFU of up to two patients per arm.

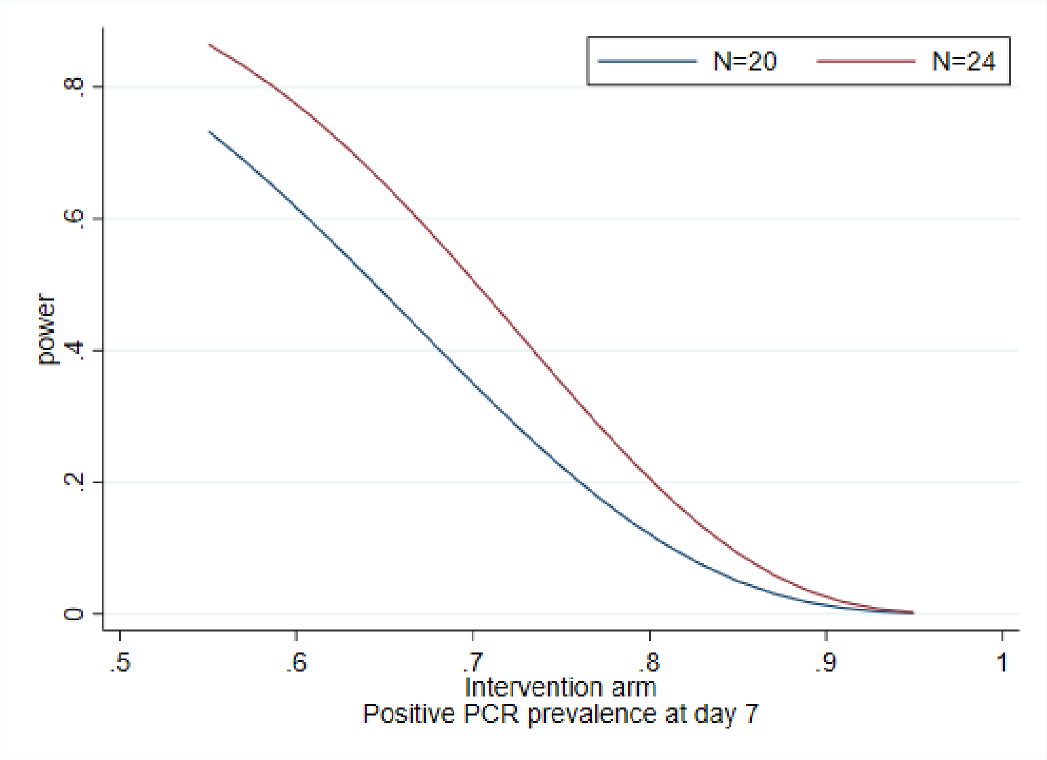

***Figure 1** Trial power scenarios for different effect size and loss to follow up levels.*

7 General Considerations

7.1 Timing of Analyses

Data will be collected using the ShareCRF platform [3], which uses automatic rules to provide high quality data. No additional cleaning process will be needed. Daily self-assessment of symptoms will be recorded by each participant in an ODK platform. The final analysis will take place after all subjects have completed the final visit or dropped out and after the finalization and approval of this SAP document.

7.1.1 Full Analysis Population

The efficacy analysis will be done per protocol (PP), including all randomized participants for whom there is a PCR result at day seven.

7.1.2 Safety Population

The safety analysis will be done using a modified intention to treat (mITT) approach considering all randomized and treated participants.

7.2 Covariates and Subgroups

No covariate nor subgroup analyses planned.

7.3 Missing Data

All the available data on safety and efficacy shall be included in the data lists and tables. No values shall be allocated for unavailable data.

Any confusing or incorrect data shall be examined in accordance with the standardized data control procedures.

In the analysis of the records reported by patients (RRP), the omitted data shall be allocated by using the last observation carried forward. The omitted standard shall be examined before any allocation. If the omitted standard is clearly informative, the repercussion of the non-random data shall be evaluated using sensitivity analysis.

For the RRP analysis, if a section of subscale from several is missing, the average of the remaining sections shall be used as a scoring scale, as long as at least half of the sections of the scale is available.

7.4 Multiple Testing

Adjustment for multiple testing will not be necessary as there is only one primary outcome.

8 Summary of Study Data

Descriptive analyses will use frequency and percentage (based on the non-missing sample size) for qualitative variables and median, interquartile range and n (non-missing sample size) for quantitative variables. Summary tables will be structured using one column per study arm and overall in the order: Placebo, Ivermectin, All subjects.

CONFIDENTIAL

8.1 Subject Disposition

The number of subjects reaching each study stage will be defined as:

- Assessed for eligibility if any information available on section “screening failures” in the baseline visit CRF.
- Randomized if study identifier given and recorded in the baseline visit CRF.
- Reached visit 2, 3, 4, 5 or final if any information available on the CRF corresponding to that visit.
- Analyzed if PCR result at day 7.

A skeleton CONSORT flow diagram is provided below.

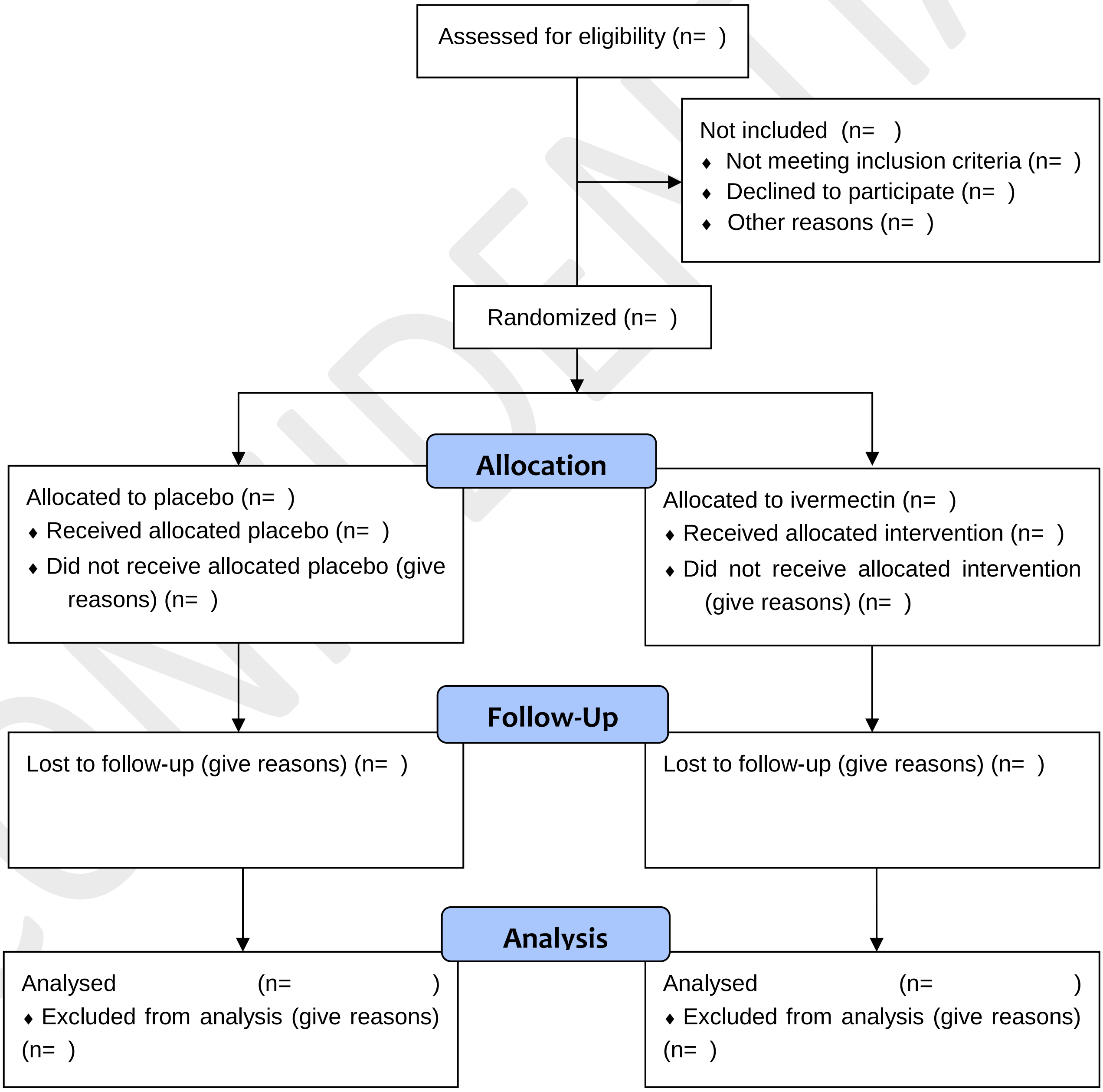

8.2 Derived variables

The following table contains the list of derived variables that will be used in the analysis.

Variable name	Description	Calculation	Data type	Coding or range
any_symptom	Any symptom reported	General OR ENT OR Respiratory OR Digestive OR Others	Integer	0: No<br>1: Yes
platelet_lymphocyte	Platelet-Lymphocyte index	Platelet / Lymphocyte	Real	0-
neutrophil_lymphocyte	Neutrophil-Lymphocyte index	Neutrophil / Lymphocyte	Real	0-
severity_pred	Severity predictor	N/L*CRP*D-dimer	Real	0-
vload_change	Viral load change	(viral load [n] – viral load [1]) / viral load [1]	Real	-
A list of variables with prefix “vk”	Primary parameters of viral kinetic	Individual curve fits and non-linear mixed effects models	Real	-

8.3 Concurrent Illnesses and Medical Conditions

No standard coding system will be used for concurrent illnesses and medical conditions. Each condition will be classified according to the affected system (Cardiovascular, Respiratory, Hepato-biliary, Gastro-intestinal, Genito-urinary, Endocrine, Hematological, Musculo-skeletal, Neoplasia, Neurological, Psychological, Immunological, Dermatological, Allergies, “Eyes, ear, nose, throat” or Other). The summary statistics will be produced in accordance to section 7.

8.4 Prior and Concurrent Medications

No standard coding system will be used for medications. The summary statistics will be produced in accordance to section 7.

8.5 Treatment Compliance

Study treatment is administered using the directly observed therapy (DOT) method. With this method, treatment adherence is ensured as a health care professional watches the study participants while they take their medication.

No treatment compliance measures will be calculated.

9 Efficacy Analyses

9.1 Primary Efficacy Analysis

The proportion of participants with positive PCR at day seven post treatment will be calculated. Proportions will be compared between study arms using Fisher’s exact test and presented as a relative risk ratio (RR), or reduction of the RR (1- RR) if RR lower than 1, and 95% Confidence

CONFIDENTIAL

Interval (CI). Differences will be considered statistically significant if the p-value obtained is lower than 0.05.

9.2 Secondary Efficacy Analyses

Secondary endpoints will be analyzed using descriptive methods as explained in section 8. Graphics such as alluvial diagrams and boxplots will be also employed to assess evolution along follow up for qualitative and quantitative variables, respectively. For each symptom and for all as a composite outcome, Kaplan-Meyer survival curves will be used to show time to improvement. The effect of study treatment on the presence of symptoms will be estimated using mixed effect logistic regression models with subject as a random intercept. These models, will be adjusted by day of follow up as symptoms are expected to disappear over time.

We will attempt to fit viral load profiles as quantified by rt-PCR to standard viral kinetics models (e.g. target cell limited models or eclipse models). Fitting strategies will comprise individual curve fits and non-linear mixed effects models. For successful fits, primary parameters of viral kinetic can be derived and compared between treatment arms. Examples include virion clearance rate, cell death rate, cellular infectivity, and acquired immune effects. Given sufficient data, secondary parameters such as peak viral load, time to peak viral load, and exposure as quantified by area under the curve can be calculated.

10 Safety Analyses

All the adverse events that occur during the study will be included in the data lists and sorted by patient and order of occurrence. Events that are considered to be related to the treatment (possibly, probably or definitely related to it) will also be included in a table. A table will with the list the adverse events according to maximum intensity will be provided. The deaths and serious adverse events will also be classified in a separate table.

Summary statistics on the number of observed events and their relatedness with the study drug will be presented by study arm and overall.

10.1 Extent of Exposure

The summary statistics will be produced in accordance to section 7.

10.2 Adverse Events

The summary statistics will be produced in accordance to section 7.

10.3 Deaths, Serious Adverse Events and other Significant Adverse Events

The summary statistics will be produced in accordance to section 7.

10.4 Pregnancies

Pregnant women will not be included in the trial. Pregnancy tests will be administered before recruitment to all women of child bearing age.

10.5 Clinical Laboratory Evaluations

CONFIDENTIAL

The summary statistics will be produced in accordance to section 7.

11 Pharmacokinetics

Not applicable.

12 Reporting Conventions

P-values greater or equal to 0.001 will be reported to 3 decimal places. P-values less than 0.001 will be reported as '<0.001'. Quantiles, such as median, or minimum and maximum will use the same number of decimal places as the original data.

13 Technical Details

The analyses will be conducted using Stata version 16 [2]. Graphs will be produced using the packages *ggplot2* [4] and *ggalluvial* [5] for R version 4.0.2. [6]. The operating system of the computer will be Windows 10.

CONFIDENTIAL

14 References

[1] P. Ryan, *RALLOC: Stata module to design randomized controlled trials,* revised 28 Jan 2018 ed., Statistical Software Components S319901, Boston College Department of Economics, 1997.

[2] StataCorp, *Stata Statistical Software: Release 16,* College Station, TX: StataCorp LLC, 2019.

[3] "ShareCRF," [Online]. Available: www.ShareCRF.com.

[4] H. Wickham, ggplot2: Elegant Graphics for Data Analysis, Springer-Verlag New York, 2016.

[5] J. C. Brunson, ggalluvial: Alluvial Plots in 'ggplot2', 2020.

[6] R Core Team, *R: A Language and Environment for Statistical Computing,* Vienna, Austria: R Foundation for Statistical Computing, 2020.

CONFIDENTIAL

15 Listing of Tables, Listings and Figures

15.1 Tables

Table 1. Baseline description of study participants

Variable	Study arm		All subjects
	Placebo	Ivermectin	
**Demographics**			
Gender: female [1]			
Age [2]			
Height (in m) [2]			
Weight (in kg) [2]			
BMI (in kg/m^2) [2]			
**Smoking habits and alcohol consumption**			
Smoker or tobacco products user [1]			
Number of cigarettes per day [2]			
Smoking duration (in years) [2]			
Alcohol consumption [1]			
Weekly alcohol consumption (in units) [2]			
**Medication**			
Taking medication [1, 3]			
**Previous medical history**			
Cardiovascular [1]			
Respiratory [1]			
Hepato-biliary [1]			
Gastro-intestinal [1]			
Genito-urinary [1]			
Endocrine [1]			
Hematological [1]			
Musculo-skeletal [1]			
Neoplasia [1]			
Neurological [1]			
Psychological [1]			
Immunological [1]			
Dermatological [1]			
Allergies [1]			
Eyes, ear, nose, throat [1]			
Others [1]			

1: n/N (Percentage)
2: Median (IQR) [n]
3: includes OTC, vitamins and supplements

CONFIDENTIAL

Table 2. Baseline clinical description of study participants

Variable		Study arm		All subjects
		Placebo	Ivermectin	
**Symptoms**				
General [1, 3]				
ENT [1,4]				
Respiratory [1]				
Digestive [1]				
Others [1]				
**Physical examination results, system affected**				
General appearance [1]				
Heart [1]				
Lungs [1]				
Abdomen [1]				
Extremities [1]				
Others [1]				
**Vital signs**				
Heart rate (in bmp) [2]				
Blood pressure (in mmHg)	Systolic [2]			
	Diastolic [2]			
Temperature (in ºC) [2]				
Oxygen saturation (in %) [2]				
Respiratory rate (in breaths per minute) [2]				
**Chest X-Ray**				
Abnormal result [1]				
**Pregnancy test**				
Test conducted [1, 5]				
Positive test result [1]				
**Laboratory results**				
Positive nasopharyngeal swab for SARS-CoV-2 [1]				
Positive for IgG antibodies [1]				
IgG antibodies (in mg/dL) [2]				
Positive for IgM antibodies [1]				
IgM antibodies (in mg/dL) [2]				
Positive for IgA antibodies [1]				
IgA antibodies (in mg/dL) [2]				
Blood creatinine (in mg/dL) [2]				
Urea blood [2]				
MDRD [2]				
LDH (in U/L) [2]				
CRP (in mg/L) [2]				

CONFIDENTIAL

Troponin T (in ng/mL) [2]			
Procalcitonin (in ng/mL) [2]			
Sodium (in mEq/L) [2]			
Potassium (in mEq/L) [2]			
Chlorine (in mEq/L) [2]			
Bicarbonate (in mEq/L) [2]			
Remaining anion (in mEq/L) [2]			
Ferritin (in ng/mL) [2]			
CPK (in Ul/L) [2]			
D-Dimer (in ng/mL) [2]			
IL-6 (in pg/mL) [2]			
**Hemogram**			
Red blood cells (in 10^{12}/L)			
Haemoglobin (in g/dL)			
Hto (in %)			
VCM (in f/L)			
HCM (in pg)			
CMHC (in g/dL)			
RDW (in %)			
Platelets (in 10^9/L)			
VPM (in f/L)			
PWD (in %)			
White blood cells (in 10^9/L)			
Neutrophil (in %)			
Neutrophil absolute value (in 10^9/L)			
Lymphocyte (in %)			
Lymphocyte absolute value (in 10^9/L)			
Monocytes (in %)			
Monocytes absolute value (in 10^9/L)			
Eosinophil (in %)			
Eosinophil absolute value (in 10^9/L)			
Basophiles (in %)			
Basophiles absolute value (in 10^9/L)			
Platelet-Lymphocyte index			
Neutrophil-Lymphocyte index			
Severity predictor?? (N/L*CRP*D-dimer)			

1: n/N (Percentage)
2: Median (IQR) [n]
3: includes General malaise, Myalgia, Appetite, Headache
4: ENT: Ear, Nose, Throat (includes anosmia and dysgeusia)
5: only women considered in this section

CONFIDENTIAL

Table 3. PCR positivity

Variable	Study arm		RR (95% CI)	p-value [2]
	Placebo	Ivermectin		
Positive PCR at day 7 [1]				
Positive PCR culture at day 4 [1]				
Positive PCR culture at day 7 [1]				

1: n/N (Column percentage)
2: Fisher's exact test
RR: risk ratio, CI: confidence interval

Table 4. Seroconversion at day 21

Variable	Study arm		All subjects
	Placebo	Ivermectin	
Seroconversion at day 21 [1]			

1: n/N (Column percentage)

Table 5. Seroconversion at day 21 in patients taking ivermectin

Variable	PCR at day 7	
	Negative	Positive
Seroconversion at day 21 [1]		

1: n/N (Column percentage)

Table 6. AEs description

Variable	Study arm		All subjects
	Placebo	Ivermectin	
Number of AEs observed			
Number of SAEs observed			
Drug-related SAEs [2]			
**Observed serious adverse events (per subject)**			
Number of SAEs during FU [1]			
Death [2]			
Body as a whole [2]			
Gastrointestinal [2]			
Nervous System/Psychiatric [2]			
Skin [2]			
…………………………………………..			

1: Median (IQR) [n]
2: n/N (Percentage)

CONFIDENTIAL

Table 7. Treatment effect on symptom presence

Variable	OR (95% CI)	p-value [1]
Treatment		
Day		

1: Mixed effect logistic regression model with subject as a random intercept

15.2 Listings

List 1. Patient baseline information

Patient	Study arm	Ct	Vital signs	Symptoms	Lab results + immuno
1	Placebo	value			
2	Placebo				
3	Placebo				
4	Placebo				
5	Placebo				
6	Placebo				
7	Placebo				
8	Placebo				
9	Placebo				
10	Placebo				
11	Placebo				
12	Placebo				
13	Ivermectin				
14	Ivermectin				
15	Ivermectin				
16	Ivermectin				
17	Ivermectin				
18	Ivermectin				
19	Ivermectin				
20	Ivermectin				
21	Ivermectin				
22	Ivermectin				
23	Ivermectin				
24	Ivermectin				

CONFIDENTIAL

List 2. List of all AEs occurred

Study arm	AE diagnosis	Drug relatedness	Severity	Action taken	Withdrawal due to AE

List 3. List of all SAEs occurred

Study arm	AE diagnosis	Drug relatedness	Severity	Action taken	Withdrawal due to AE

15.3 Figures

Figure 1. Viral load evolution during FU (days 1, 4, 7, 14 and 21) by study arm

This figure will contain a boxplot for each day and study arm.

[boxplots]

Figure 2. Viral load change during FU (days 1, 4, 7, 14 and 21) by study arm

A boxplot for each day and study arm.

[boxplots]

Figure 3. Viral load evolution during FU (days 1, 4, 7, 14 and 21) by study participant

Separate line plots that will show each subject's evolution during FU.

[line plots]

Figure 4. Symptom presence during FU (days 1, 4, 7, 14 and 21) by study arm

One diagram for each symptom considered: General (General malaise, Myalgia, Appetite, Headache), ENT (includes anosmia and dysgeusia), Respiratory and Digestive.

[Alluvial diagram]

CONFIDENTIAL

Figure 5. System abnormalities identified during FU (days 1, 4, 7, 14 and 21) by study arm

One diagram for each system considered: General Appearance, Heart, Lungs, Abdomen and Extremities.

[Alluvial diagram]

Figure 6. Time to symptom improvement by study arm

One graph for the composite outcome "any symptom" and one for each symptom considered: General (General malaise, Myalgia, Appetite, Headache), ENT (includes anosmia and dysgeusia), Respiratory and Digestive.

[Kaplan-Meier curve]

Figure 7. Time to severe disease or death by study arm

[Kaplan-Meier curve]

CONFIDENTIAL
